# DISTILLER: a data integration framework to reveal condition dependency of complex regulons in *Escherichia coli*

**DOI:** 10.1186/gb-2009-10-3-r27

**Published:** 2009-03-06

**Authors:** Karen Lemmens, Tijl De Bie, Thomas Dhollander, Sigrid C De Keersmaecker, Inge M Thijs, Geert Schoofs, Ami De Weerdt, Bart De Moor, Jos Vanderleyden, Julio Collado-Vides, Kristof Engelen, Kathleen Marchal

**Affiliations:** 1 Department of Electrical engineering, Katholieke Universiteit Leuven, Kasteelpark Arenberg 10, 3001 Leuven, Belgium; 2 Department of Engineering Mathematics, University of Bristol, Bristol BS8 1TR, UK; 3 OKP Research Group, Katholieke Universiteit Leuven, Leuven 3000, Belgium; 4 Department of Microbial and Molecular systems, Katholieke Universiteit Leuven, Kasteelpark Arenberg 20, 3001 Leuven, Belgium; 5 Centro de Ciencias Genómicas, Universidad Nacional Autónoma de México, Cuernavaca AP 565-A, México

## Abstract

We present DISTILLER, a data integration framework for the inference of transcriptional module networks. Experimental validation of predicted targets for the well-studied fumarate nitrate reductase regulator showed the effectiveness of our approach in *Escherichia coli*. In addition, the condition dependency and modularity of the inferred transcriptional network was studied. Surprisingly, the level of regulatory complexity seemed lower than that which would be expected from RegulonDB, indicating that complex regulatory programs tend to decrease the degree of modularity.

## Abstract 2

DISTILLER, a data integration framework for the inference of transcriptional module networks, is presented and used to investigate the condition dependency and modularity in Escherichia coli networks.

## Background

The transcriptional network of *Escherichia coli *is among the best characterized transcriptional networks [1]. Based on our current knowledge of this network it is clear that complex regulons [2] are prevalent: more than 50% of the genes are regulated by more than one transcriptional regulator [2,3]. However, most of these complex regulons were inferred by curating experimental evidence for a regulator-target interaction from independent studies, each of which focuses on an individual interaction [3]. Evidence from these independent studies is obtained from measurements in different environmental conditions. Current network representations do not take into account this condition dependency of the regulatory interactions [4,5]. As a consequence, it is not clear from these static networks whether regulators controlling the same gene are indeed needed together in the same conditions or act independently of each other in different conditions [6,7].

Bicluster strategies are well suited to map both the condition dependency and the modularity of the transcriptional network from microarray compendia [8-11], but do not give any information on the transcriptional program of the modules. Methods have been developed to infer transcriptional interactions from microarrays only, by assuming that the transcription profile of the regulator is related to that of its target genes [12-14]. Integrative approaches can avoid this assumption by exploiting data sources that are complementary to microarrays. These methods have been successfully used to infer simple regulons [15-17] or to directly infer complex regulons, that is, the set of genes regulated by several regulators [18-20]. Most of the previously mentioned integrative approaches use the level to which the target genes of a particular regulator share a similar expression pattern as a feature for inferring regulator-target interactions, but do not include an explicit condition selection strategy as is the case with bicluster strategies [11]. A few exceptions exist, including the graph-based data integration tool SAMBA [20] and the sequential approach described by Bonneau *et al*. [21]. The latter approach searches simultaneously for biclusters and *de novo *motifs in the promoter region of the bicluster genes by using cMonkey [22], and subsequently applies a regression strategy [21], to associate a regulatory program with the inferred biclusters.

To study the yet unknown relation between modularity, combinatorial regulation and condition dependency of bacterial networks, we developed the data integration framework 'DISTILLER' (Data Integration System To Identify Links in Expression Regulation). DISTILLER simultaneously identifies condition-dependent modularity and complex regulatory programs by integrating expression data and interaction data.

## Results

DISTILLER is a data integration framework that searches for condition-dependent transcriptional modules by combining expression data with information on the direct interaction between a regulator and its corresponding target genes. The framework builds upon advanced itemset mining approaches that are efficient and intuitive to use and, therefore, well suited for solving combinatorially complex problems like the one proposed here. The drawback of the itemset mining approaches compared to more commonly used graph-based or probabilistic methods is that by being exhaustive, they enumerate all possible ('valid') solutions in a deterministic way without explicitly assessing their statistical significance. Hence, predicted interactions are not statistically prioritized, making it harder to interpret the reliability of the results. For the purpose of this study we developed a method that combines the advantages associated with the efficiency of an itemset mining search strategy with those related to statistical scoring measures. DISTILLER allows an efficient simultaneous search for genes that are co-expressed, the conditions in which the genes are co-expressed and the regulators that are responsible for the observed co-expression. In other words, it simultaneously identifies biclusters and their complex regulatory programs. Obtained modules are prioritized by assigning a score based on their statistical significance and overlap with previously identified modules (Materials and methods).

In this study, we applied DISTILLER to simultaneously analyze two complementary data sources: a novel cross-platform expression compendium consisting of 870 *E. coli *microarrays and a regulatory motif compendium consisting of both predicted and experimentally verified motif instances (see Materials and methods).

### Inferring regulator-target interactions by exploiting the network's modularity

By integrating motif data and a large scale expression compendium, DISTILLER detects condition-dependent regulatory modules. From each module, regulator-target interactions that are functionally active under the experimental conditions included in the module can be extracted by linking each gene in the module with the regulator(s) corresponding to the shared motif instance(s). The 150 statistically most significant modules recovered by DISTILLER represent a total of 732 interactions. Of these, 454 interactions correspond to 62% of 736 interactions for 67 regulators with known binding sites described in RegulonDB [3] (see Additional data file 1 and our supplementary website [23] for a detailed description of the modules). Most modules are enriched for functions in which the regulator was known to be involved. For 37 of the 67 regulators at least part of their regulon could be confirmed. For the remaining 30 regulators no interaction was found; most likely either the number of genes in the corresponding modules falls below the gene content threshold, or the conditions needed to trigger these interactions are not present in our compendium, for example, MelR, triggered by melibiose.

In addition to identifying 454 previously described interactions, we predict 278 novel interactions that have not yet been documented in RegulonDB (Additional data file 2). For many well studied regulators, the known part of their regulon could be considerably extended. As for most of the newly predicted interactions, no additional confirmation existed in the literature, and we assigned them a level of confidence based on the gene composition of the module from which the target was retrieved. If the module contained many previously confirmed targets, tightly co-expressed with the novel target, we attached larger confidence to its prediction(s).

To demonstrate the reliability of our approach, we used chromatin immunoprecipitation followed by quantitative PCR (ChIP-qPCR) to validate predicted interactions for the fumarate and nitrate reductase regulator (FNR), one of the most extensively studied regulators in *E. coli *(see Materials and methods). DISTILLER recovered 48 of the 57 FNR targets described in RegulonDB, and predicted 25 novel FNR targets, four of which (*ung, ompW*, *ydfZ *and *ynfK*) were confirmed by a recent ChIP on chip (ChIP-chip) analysis [24]. We tested 11 additional targets that were selected based on their difference in prediction confidence. These 11 predictions, consisting of four high confidence predictions (*ydhY*, *yfgG*, *hscC *and *treF*) and seven medium confidence predictions (*yjhB*, *ydjX*, *yjtD*, *ydaT*, *yehD*, *yhjA *and *ftnB*), were all shown to bind FNR *in vivo*. In the course of this study, two of these validated FNR targets, *yhjA *and *ydhY*, have also been confirmed by two independent experimental studies [25,26].

### Conditional dependency of the regulatory network

**Figure 1:**
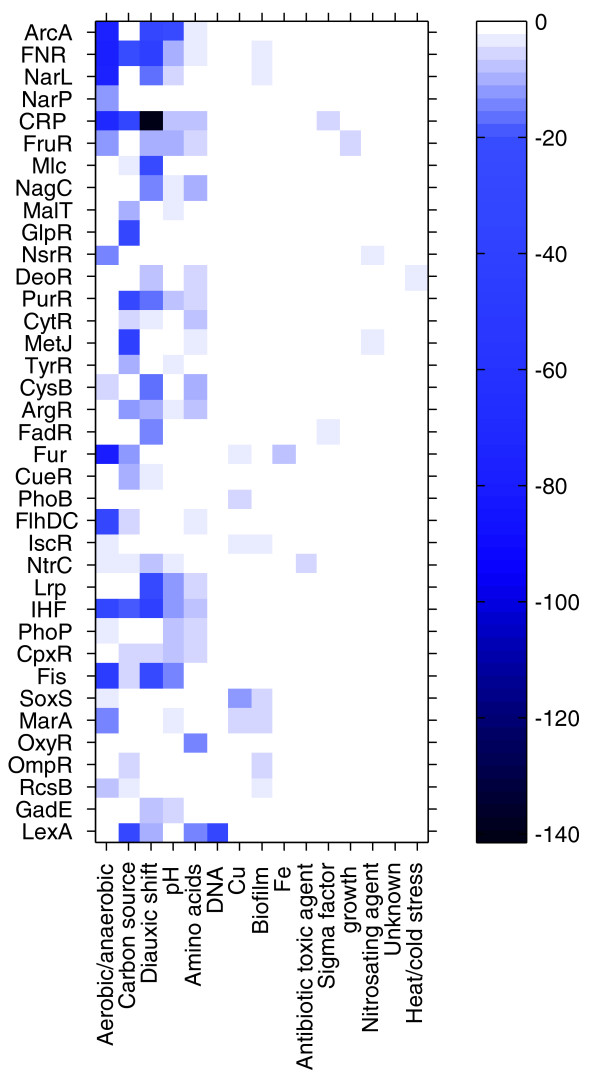
Condition dependency of the regulatory modules. Columns are conditional categories, and rows are regulators for which modules were detected by DISTILLER. Each entry indicates to what extent the conditions of the modules of a particular regulator are enriched (log *P*-value) for a specific category. Dark blue entries correspond to the most significant enrichments.

Our method not only extends the existing network by predicting regulator-target interactions but also extracts information on the condition dependency of these interactions. Arrays were grouped into conditional categories depending on the major cue that was changed in the experiments (Additional data file 3). For instance, the category 'aerobic-anaerobic' groups all arrays in which the effect of changing the oxygen level on gene expression was measured. Figure 1 shows to what extent the conditions of the modules of a particular regulator are enriched for a specific category. (For a full description of Figure 1, see Additional data file 3.) Enrichment of a conditional category implies that the target genes of a particular regulator are mainly co-expressed in conditions belonging to the enriched category; this indirectly gives information on the conditions where a particular regulator is active. Most regulators were found to be active in conditions that are in agreement with their annotation, illustrating the effectiveness of our condition selection (bicluster) strategy.

Environmental conditions that trigger major changes in the energy status of a cell seem to have the most pronounced effect on transcriptional regulation: changes in oxygen concentration, diauxic shift, pH and carbon source trigger a whole range of transcriptional regulators that mediate the transition to a novel metabolic state. Other conditions seem to trigger very specific pathways. Changes in the Fe^2+ ^concentration or application of DNA damage, for instance, induce the Fur and LexA pathways, respectively.

The role of global regulators such as ArcA, Fis, FNR, Lrp, cAMP-receptor protein (CRP) or integration host factor (IHF) that tune the overall cellular response towards the simultaneous interplay of energy, carbon source and amino acid availability is clearly visible. The more conditional categories a regulator is involved in, the more global its role. For instance, CRP and the nucleoid associated proteins Fis and IHF are the most pleiotropic regulators [27], but FruR, Fur, and LexA also seem to have a considerable impact on gene expression in a variety of conditions. In contrast to the global regulators, more specific regulators are important for fine-tuning the response. For instance, modules of GlpR, involved in the regulation of glycerol catabolism, are mainly expressed in the conditional category 'carbon source', but only upon addition of glycerol to the medium. Also, there are subtle differences between the paralogs Mlc and NagC: Mlc is mainly active during diauxic shift (conditional category 'diauxic shift'), while NagC modules are also linked to 'amino acids' conditions [28].

OmpR, a known major regulator of membrane remodeling during growth on biofilms [29], and RscB, another known regulator of growth during biofilm formation [30], are indeed overrepresented in 'biofilm' conditions but may also play a role in alterations of the carbon source (both OmpR and RscB) or in oxygen changes, that is, 'aerobic-anaerobic' (only RscB). Although CpxR has recently been described as a biofilm related regulator [31], it does not seem to be overrepresented in the biofilm related conditions present in our compendium, but mainly during pH shifts [32].

### Regulation of modules by multiple transcription factors

DISTILLER also identifies the level of combinatorial regulation of the target genes within each module. With combinatorial control we refer here to the fact that a set of genes is regulated by at least two different regulators, irrespective of whether these regulators effectively undergo complex interactions or act independently of each other. According to RegulonDB, 42 transcription units (operons) are regulated by one regulator, 66 by two regulators and 70 by three or more regulators (with a maximum in-degree of eight regulators for a single transcription unit). Our inferred modules do not seem to exhibit the same amount of regulatory complexity: in our data set, only 25 modules out of 150 were found to be regulated by at least two regulators and the maximum level of multiple regulation at the module level was restricted to three regulators. Out of 25 modules regulated by at least two regulators, 24 modules involve at least one global regulator such as CRP, FNR or ArcA, confirming the role of global regulators as hubs in the 'co-regulatory network' [6]. To test whether this low number of modules that are regulated by multiple regulators is not due only to the fact that the number of complex regulons annotated in RegulonDB is lower than the number of simple regulons, we calculated the number of complex regulons containing at least four genes (four operons) in RegulonDB: 283 interactions belong to complex regulons of at least two regulators. Only 83 of these 283 interactions (29%) were actually found co-expressed in our modules. In contrast, of the total of 663 interactions in RegulonDB that belong to simple regulons of at least four genes, 398 interactions (60%) were present in our transcriptional modules. Thus, the fraction of genes that share a single transcription factor and are co-expressed is significantly larger than the fraction of genes that share at least two transcription factors and are co-expressed.

**Figure 2:**
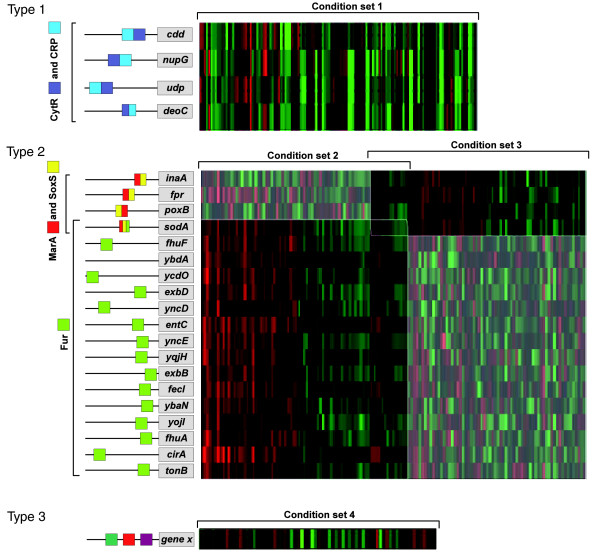
Types of combinatorial regulation. Type 1 shows combinatorial regulation at the module level. The genes *cdd*, *nupG*, *udp *and *deoC *have two motifs in common (corresponding to the regulators CytR and CRP) and are co-expressed in condition set 1. This kind of control often seems to occur as a combination of a global regulator and a more specific one. Type 2 shows combinatorial regulation at the level of a connector gene. All genes of module 1 share two motifs, MarA and SoxS, and are co-expressed in a subset of conditions. For module 2 all genes are regulated by Fur. *SodA*, a connector gene, is shared by both modules and is thus regulated by the regulators of module 1 and module 2, but under a different set of conditions (as shown by the heatmap image), indicating that the corresponding regulators of both modules act independently of each other. Both types of interactions mentioned above can be identified by DISTILLER. Cases where condition-specific complex interactions between regulators result in such highly gene-specific expression patterns that genes are no longer found co-expressed in modules (type 3) cannot be detected by DISTILLER.

However, this low level of control by more regulators at the module level does not exclude that the expression of individual genes is often influenced by more than one regulator. We identified 85 'connector genes' in our modules (Figure 2). These are individual genes that are shared by distinct modules, each of which is controlled by different regulators. Modules sharing the same connector gene often show little overlap in their conditions, suggesting that one regulator may, in many cases, be sufficient to alter the expression of a connector gene upon a specific environmental cue. One example of such a connector gene is the SodA gene product, manganese superoxide dismutase [33-35]. The gene *sodA *is present in a module regulated by MarA and SoxS and in a module regulated by Fur, coupling its expression to multiple antibiotic resistance (MarA), superoxide (SoxS) resistance and the intracellular iron pool (Fur). For other genes, the expression behavior may be highly specific and is therefore never shared with enough other genes to meet our gene content threshold (Figure 2). Those genes cannot be found in transcriptional modules.

### Comparison with other methods

**Figure 3:**
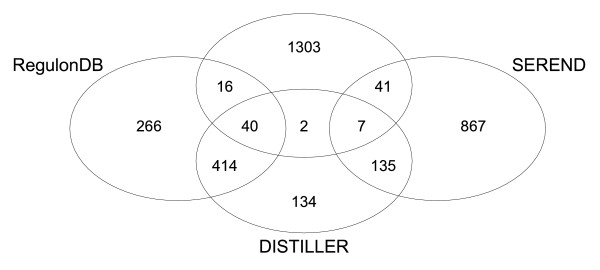
Venn Diagram showing the number of overlapping interactions between the networks of RegulonDB, CLR, SEREND and DISTILLER. CLR, SEREND and DISTILLER were applied to our data sets. As the overlap between SEREND and RegulonDB is algorithmically defined to be 100%, we show only the predictions of SEREND that were not reported in RegulonDB and do not explicitly visualize the overlap with RegulonDB for SEREND.

We compared our results with those of two recently published network reconstruction methods in order to assess the reliability of our predictions and the complementarity between the approaches. We selected the context of likelihood relatedness (CLR) method by Faith *et al*. [14], which relies only on microarray data to infer interactions between regulators and target genes and the semi-supervised regulatory network discoverer (SEREND) by Ernst *et al*. [17]. Both methods have initially been applied to *E. coli *data and their software was available. Moreover, the goal of SEREND [17] best resembles our aim: the optimal use of complementary available data sources to extend the known regulatory network in a reliable way. For comparison with CLR [14] and SEREND [17], we only compared the interactions inferred for those 67 regulators for which a binding site was described in RegulonDB. Note that CLR and SEREND can, in theory, also predict interactions for regulators without known binding sites. The results of the comparisons are summarized in Figure 3.

Faith *et al*. [14] developed CLR to infer regulator-target interactions from an *E. coli *Affymetrix compendium [14]. Their method is an extension of the 'relevance networks approach' where an interaction between a regulator and a target gene is predicted if the mutual information between the expression profiles of the target and the regulator exceeds a certain threshold. CLR was applied to our expression compendium to evaluate the results obtained by CLR and DISTILLER (see Materials and methods). The threshold z-score, a parameter of CLR, was chosen so as to maximize the overlap between the CLR inferred network and the known RegulonDB [3] network (Additional data file 4). The interactions reported by CLR and DISTILLER show a low overlap: only 40 known and 9 novel interactions were identified by both methods (Figure 3). Only 56 of all the interactions recovered by CLR were reported in RegulonDB. Additional comparisons of CLR and DISTILLER for different choices of the CLR z-score threshold were performed (Additional data file 4). In general, changing the z-score thresholds does not influence the conclusions mentioned above. The observed low overlap between DISTILLER and CLR reflects the fundamental differences in the underlying assumptions and working principles of both methods: while DISTILLER focuses on data integration, condition dependency, modularity and regulation by combined sets of transcription factors, CLR was designed to deal with gene-specific expression profiles.

In contrast to CLR and similar to DISTILLER, SEREND [17] does not rely on the assumption that a transcription factor has an expression profile that is directly related to the profile of its target genes. SEREND [17] applies an iterative classification scheme that exploits existing knowledge on regulator-target interactions in a semi-supervised way in order to predict novel interactions for these regulators. Ernst *et al*. [17] train a model using expression and regulatory motif data for confirmed regulator-target interactions. Subsequently, novel interactions can be inferred using their model on expression and regulatory motif data. Unknown interactions are classified using a co-expression score and a motif score. A prediction between a regulator and a target gene will be ranked as highly reliable if the predicted target gene contains a motif instance similar to the motif instances in the known target genes of that regulator and if the target gene is co-expressed with the previously described targets of that regulator. Using their model, Ernst *et al*. [17] could thus extend the known regulatory network.

By applying SEREND to our microarray and regulatory motif compendia, 1,049 novel interactions were obtained. These interactions were compared with the interactions identified by DISTILLER and CLR. Note that as SEREND uses the information of RegulonDB as training information, it will always recover interactions reported in RegulonDB as the highest scoring ones. The overlap between SEREND and RegulonDB is thus algorithmically enforced to be 100%. An explicit comparison between SEREND and RegulonDB is, therefore, not shown and we include only the 1,049 novel predictions made by SEREND in our comparison (Figure 3). Of these 1,049 novel interactions, DISTILLER and SEREND inferred 142 identical ones. In comparison, the observed overlap between CLR and SEREND was much lower and contained only 48 identical novel predictions. In total, the three methods have only seven interactions in common.

In general, the overlap between all three methods is thus rather low. DISTILLER agrees most with SEREND and the lowest overlap between the results was observed in the comparison between DISTILLER and CLR. This is to be expected as both DISTILLER and SEREND are integrative approaches designed to make less but more reliable predictions while CLR makes use of completely different underlying assumptions.

Although the previous comparison indicates that DISTILLER and SEREND resemble each other the most while CLR behaves quite differently, we can not judge the reliability of the novel interactions. As RegulonDB is used as input for SEREND and DISTILLER, we cannot fairly compare the ratio of novel/known interactions (or the precision versus recall). For this reason we also performed a benchmark using ChIP-chip data as a gold standard because they are the only currently available benchmark resource that is independent from RegulonDB. We therefore compared the interactions inferred by each of the methods with the interactions that were identified for five regulators (FNR, CRP, Fis, IHF, and heat-stable nucleoid-structuring protein (H-NS)) in a series of independent ChIP-chip experiments [24,36,37]. In general, SEREND [17] scored better than DISTILLER in terms of recall but at the expense of precision (Tables 1 and 2). For CLR both the recall and precision are, in general, lower than those observed for the other two methods. To compare the obtained recall and precision in detail, we adapted the score thresholds of both SEREND and CLR to work with the same precision-recall trade-off as DISTILLER for each individual regulator. From these results it appears that DISTILLER performs at least as equally well as SEREND [17] or CLR for most regulators when taking into account the precision-recall trade-off. In other words, for the same number of predictions that were confirmed in ChIP-chip experiments, DISTILLER outputs less false-positive predictions than SEREND or CLR. A detailed description of the analysis can be found in Additional data file 5. By aiming at a high precision, DISTILLER is an interesting method to support wet lab research.

**Table 1:** Comparison of interactions confirmed in RegulonDB and identified by ChIP-chip experiments, CLR, SEREND and DISTILLER for five global regulators

	Confirmed RegulonDB				
	
	Not ChIP-chip	ChIP-chip	Total	Recall	Precision
**FNR**					
ChIP-chip	-	21	21		
CLR	0	0	0	0	0
SEREND	-	-	-	-	-
DISTILLER	29	19	48	0.90	0.40
					
**CRP**					
ChIP-chip	-	31	31		
CLR	3	0	3	0	0
SEREND	-	-	-	-	-
DISTILLER	90	21	111	0.68	0.19
					
**Fis**					
ChIP-chip	-	5	5		
CLR	1	0	1	0	0
SEREND	-	-	-	-	-
DISTILLER	18	3	21	0.60	0.14
					
**H-NS**					
ChIP-chip	-	0	0		
CLR	0	0	0	0	0
SEREND	-	-	-	-	-
DISTILLER	0	0	0	0	0
					
**IHF**					
ChIP-chip	-	8	8		
CLR	4	0	4	0	0
SEREND	-	-	-	-	-
DISTILLER	32	7	39	0.88	0.18

For each method, the identified interactions that were known as compared to RegulonDB were selected. For all known interactions, we indicate whether (ChIP-chip) or not (Not ChIP-chip) the interactions were found in a corresponding ChIP-chip experiment. The recall (TP/TP + FN) and precision (TP/TP + FP) were calculated using the ChIP-chip data as a gold standard. Interactions identified by either CLR, SEREND or DISTILLER and confirmed by a ChIP-chip experiment were considered to be true positives (TP); interactions confirmed by a ChIP-chip experiment but not identified by either CLR, SEREND or DISTILLER were considered false negatives (FN); interactions identified by either CLR, SEREND or DISTILLER but not confirmed in a ChIP-chip experiment were considered false positives (FP). Note that since all interactions of RegulonDB are recovered by SEREND by definition (algorithmic consequence of using RegulonDB as a training set), a comparison with SEREND was not possible here.

**Table 2:** Comparison of novel interactions identified by ChIP-chip experiments, CLR, SEREND and DISTILLER for five global regulators

	Predictions				
	
	Not ChIP-chip	ChIP-chip	Total	Recall	Precision
**FNR**					
ChIP-chip	-	73	73		
CLR	14	0	14	0	0
SEREND	76	19	95	0.26	0.20
DISTILLER	21	4	25	0.055	0.16
					
**CRP**					
ChIP-chip	-	57	57		
CLR	23	0	23	0	0
SEREND	203	9	212	0.16	0.042
DISTILLER	57	6	63	0.11	0.095
					
**Fis**					
ChIP-chip	-	179	179		
CLR	59	4	63	0.022	0.06
SEREND	33	4	37	0.022	0.11
DISTILLER	17	3	20	0.017	0.15
					
**H-NS**					
ChIP-chip	-	82	82		
CLR	26	0	26	0	0
SEREND	4	0	4	0	0
DISTILLER	0	0	0	0	0
					
**IHF**					
ChIP-chip	-	110	110		
CLR	67	4	71	0.036	0.056
SEREND	79	10	89	0.091	0.11
DISTILLER	14	4	18	0.036	0.22

For each method, the identified interactions that were novel as compared to RegulonDB were selected. For all novel interactions, we indicate whether (ChIP-chip) or not (Not ChIP-chip) the interactions were found in a corresponding ChIP-chip experiment. The recall (TP/TP + FN) and precision (TP/TP + FP) were calculated using the ChIP-chip data as a gold standard. Interactions identified by either CLR, SEREND or DISTILLER and confirmed by a ChIP-chip experiment were considered to be true positives (TP); interactions confirmed by a ChIP-chip experiment but not identified by either CLR, SEREND or DISTILLER were considered false negatives (FN); interactions identified by either CLR, SEREND or DISTILLER but not confirmed in a ChIP-chip experiment were considered false positives (FP).

## Discussion

Data integration frameworks like DISTILLER can enhance gene annotation by exploiting publicly available data in combination with curated information. The main difference of our approach compared to most previously developed algorithms is its ability to explicitly derive both the conditions under which the interactions take place and the combination of regulators that are responsible for the observed expression. This more detailed level of annotation will become increasingly important with the inclusion of a growing number of experiments and conditions in available expression compendia. DISTILLER is a generic method and can thus be applied to any organism, including eukaryotes. Both for computational reasons and interpretability, it is advisable, however, to either apply filtering (such as using expression data sets related to one tissue or one process only) or use more stringent parameter settings and/or more different constraints (such as the combined use of motif and ChIP-chip data) for these more complex organisms.

In this work we applied DISTILLER to the bacterial model organism *E. coli *to study the condition dependency and combinatorial nature of its network. By applying DISTILLER to the binding site information and microarray compendium, we confirmed 62% of the known transcriptional interactions in *E. coli *and extended the regulons of 29 regulators with 278 putative novel targets. To demonstrate the effectiveness of our approach, we chose to validate predicted interactions for FNR. Because FNR is one of the best studied regulators in *E. coli *and genome-wide ChIP-chip experiments are available [24], finding new targets for this regulator is particularly challenging. In spite of this fact, we selected 11 predictions that have not been reported in previous studies and experimentally demonstrated a physical interaction with FNR for all of them using a ChIP-qPCR analysis.

Considering the condition dependency of transcriptional regulation opens a novel perspective on the transcriptional network. Although our results are preliminary and based only on a fraction of well characterized regulators, they reveal a first glimpse of real condition-dependent modularity in the *E. coli *transcriptional network. It seems that modularity in co-expression exists at the level of a single regulator, but that combinatorial regulatory programs seem to decrease the level of modularity and contribute to the network's evolvability [38]: the fraction of genes sharing a single transcription factor for which significant co-expression was detected was significantly larger than the fraction of genes sharing at least two transcription factors for which the co-expression constraint is satisfied. Combinatorial regulation inserts connections between different modules (through so-called connector genes) or generates novel gene specific expression behavior that is not shared with other genes. The apparently large tolerance of prokaryotes for disruption of modularity may at least partially be explained by the existence of polycistronic transcription: a minimal degree of modularity in expression is always guaranteed by the operon structure [6].

## Conclusions

In this study we have applied the data integration framework DISTILLER to a combination of publicly available microarray data and regulatory motif data. This allowed us to considerably extend the transcriptional network with novel interactions for regulators described in RegulonDB. The reliability of the predictions was assessed by experimental validation of novel FNR target genes. Our study also gives a first glimpse at the modularity and condition dependency of the interaction network in *E. coli*.

## Materials and methods

### Expression data

Our cross-platform compendium contains a collection of 870 publicly available microarrays, representing a plethora of diverse experimental conditions (data available upon request). The data were collected from the three major microarray databases: Stanford Microarray Database [39], Gene Expression Omnibus [40], and ArrayExpress [41]. Additionally, we added four microarray experiments described in the literature that were available as supplementary information. The microarray compendium and the required normalization procedures to allow for cross-experiment and cross-platform comparability are described in Additional data file 6. All experimental platforms contributed equally to our modules irrespective of the platform from which they originated, indicating that cross-platform biases were sufficiently removed by the appropriate preprocessing. Before applying DISTILLER, normalized data were converted to ranks (Additional data file 6).

### Regulatory motif data

The input interaction data were based on both experimentally verified and predicted regulatory binding sites. To predict novel binding site instances, motif weight matrices corresponding to the binding sites of 67 regulators were downloaded from the RegulonDB website (version 5.6) [3]. Upstream regions on the direct strand of all annotated *Escherichia coli *K12 [Genbank:NC_000913] genes were screened with these motif matrices in order to find novel motif instances. These upstream regions include the intergenic region between the gene of interest and its upstream gene and the first 50 nucleotides of the genes' coding region. If an upstream region was smaller than 150 nucleotides, it was extended with the region overlapping the coding region of the previous gene until a maximum of 150 nucleotides was reached. The average length of the intergenic region was 253 bp. For motif screening and *P*-value calculations of the identified motif instances, we used the method of Hertzberg *et al*. [42]. The *P*-values were used to construct the 'motif matrix', a binary matrix that assigns a motif instance to a gene whenever the gene's upstream sequence contains at least one instance of the motif, with a *P*-value below a threshold of 0.001.

Known binding sites in the motif matrix were derived from RegulonDB [3]. Whenever a motif instance in the promoter region of a gene was experimentally confirmed according to RegulonDB, its corresponding regulator-target interaction was set to '1' in the motif matrix, irrespective of its motif screening *P*-value. The 34 motif instances present in the upstream sequences of non-coding RNAs (tRNA or miscellaneous RNA) were omitted. The resulting motif matrix was used as input for DISTILLER and contains a total of 736 experimentally verified and 830 predicted motif instances.

Note that since only the first operon gene will contain the motif in its promoter region, the interactions presented in this motif matrix will not involve downstream operon genes. These additional operon genes are recovered in the seed module extension step (see below).

### Data integration

The core of our framework is a data integration strategy that relies on itemset mining. In our previous work [19] we already showed that approaches based on itemset mining are as equally suitable for reconstructing networks as the more frequently used graph-based [43] or probabilistic methodologies [12]. Although both our previous and our current approach are based on item set mining, the setup of DISTILLER is completely different from that used in ReMoDiscovery [19]. In contrast to ReMoDiscovery, DISTILLER not only searches for sets of highly co-expressed genes that share controlling regulators, but also selects the experimental conditions for which the selected genes are co-expressed. By including this 'bicluster strategy' genes are no longer required to be co-expressed over all conditions. This allows the algorithm to be applied to heterogeneous expression compendia in order to assess the condition dependency of the interaction network. Extending itemset mining approaches to biclustering is a non-trivial task since commonly used distance measures for assessing co-expression such as correlation no longer meet the basic subset relation constraints of an itemset mining framework. We therefore designed a novel distance measure (see below). Since the condition selection increases the combinatorial nature of the problem, DISTILLER relies on the closed itemset mining strategy CHARM [44] instead of Apriori [45]. This change in itemset mining algorithm not only made the search for modules more efficient, but also drastically reduced the number of user-defined parameters, thereby enhancing the interpretability of the results.

One of the main advantages of itemset mining approaches in comparison to 'optimization-based' methods is that they investigate all potentially interesting solutions (in this case, modules) and, thus, are not subject to problems associated with local optima. However, this also implies that the output of virtually all itemset mining algorithms is a long list of possibly interesting results without rigorous statistical significance scores. In order to make interpretation of such lists feasible, we introduced in this work an intelligent filtering step that is based on a statistically inspired interest score. The result is a concise list of statistically significant and biologically interesting modules. Although in this study we applied our method only to an expression compendium and motif data, other data sources related to transcriptional interactions, such as additional microarrays or ChIP-chip, can be integrated as well with our approach.

The DISTILLER software is available upon request. A more detailed explanation of DISTILLER and its running parameters is given in Additional data file 7.

Our methodology consists of three steps (Figure S1 in Additional data file 7): step 1, the identification of seed modules; step 2, the reduction of the set of all seed modules to a manageable set of non-redundant and statistically significant seed modules; step 3, the extension of the thus obtained seed modules with additional genes.

#### Identification of seed modules

Valid seed modules are seed modules that contain a minimal number of genes (that is, a gene content threshold) that are co-expressed in a sufficiently large number of conditions and share motif instances for the same regulator(s). A naïve exhaustive search for valid seed modules would require checking all possible combinations of genes, motif instances, and experimental conditions. This is unfeasible for data sets of any reasonable size. In addition, allowing modules to be co-expressed in only a subset of the conditions significantly increases the computational requirements. Relying on the Apriori algorithm [45], such as described in our previous approach [19], would no longer be computationally tractable. To find valid modules more efficiently, we developed an approach based on the itemset mining algorithm called CHARM [44] that drastically restricts the search space without running the risk of skipping valid modules. CHARM can be used to efficiently limit the number of combinations to be tested if different itemsets (or gene sets) are related to each other by a valid 'subset' relation, meaning an itemset can satisfy all constraints only if all of its subsets do. A consequence is that we can search for modules by starting with very small gene sets (containing just one gene), gradually expanding them, and stopping (or pruning) the search once a gene set is reached for which one of the module properties is violated. This pruning step results in a massive speed-up, making the method applicable to large data sets.

Implementing this subset relation for the integration of the motif data is straightforward as the motif matrix is a binary matrix: a target gene has a motif instance for a regulator if the corresponding gene-regulator entry in the motif matrix is equal to one. However, a more involved strategy, including a clever definition of 'sufficient co-expression', is needed to allow the use of a similar subset relation for condition selection in the expression matrix. To this end we used the concept of the bandwidth, which is defined as the difference between the largest and smallest expression levels in the gene set (Additional data file 7). Using a fixed bandwidth threshold for the condition selection would be suboptimal because randomly selected genes may also appear co-expressed in certain conditions. This could be thought of as a multiple testing effect: if there are many conditions, it is likely that some conditions will have a small bandwidth (that is, in which the genes appear co-expressed) for these random genes. To compensate for this effect, we introduce the notion of a bandwidth sequence, that is, the set of bandwidths for all conditions sorted in increasing order. This bandwidth sequence is compared with a threshold bandwidth sequence obtained by randomization: genes are said to be co-expressed in a set of conditions if their bandwidth sequence is completely within the threshold bandwidth sequence. The threshold bandwidth sequence is defined such that we are more restrictive in selecting the condition with the smallest bandwidth (as if applying a multiple testing correction), slightly less restrictive for the second smallest bandwidth (as if applying a step-down correction), and so on.

#### Selection of interesting non-redundant modules

Despite the massive reduction in the number of modules achieved by using the CHARM algorithm, the output may still be too large to explore. As no explicit score is assigned to the modules, it is not clear which modules are 'most interesting' to analyze. Also, the output might contain partially redundant modules: noise in the data may cause modules to appear as a number of separate, partially overlapping modules - for instance, differing from each other in a few conditions only. We further prioritized this unranked list of modules by iteratively assigning an interest score to each of the modules. The interest score takes into account the significance of the individual modules but, at the same time, penalizes overlap with modules that have already been reported. Thus, interesting modules are selected one by one depending on their statistical significance and the extent to which they contribute to the covering of the complete solution space and, thus, do not overlap with modules that had already been selected.

#### Seed module extension

In a subsequent extension step we recruit additional candidate module genes that did not pass the stringent seed discovery step but should be considered part of the module (for example, downstream operon genes that do not contain a motif instance in their promoter regions but are subject to its regulatory influence). The relaxed criteria for adding additional genes to the module are the following: the gene's expression profile should have a correlation with the module's mean expression profile of at least 0.9 of the module correlation (defined as the lowest correlation value between a seed gene's expression profile and the average expression profile for the modules conditions); and the genes should have a motif instance with a *P*-value below the threshold 0.05. Both requirements have to be fulfilled unless a gene is part of an operon for which the first gene is present in the seed module. In this case only the first criterion has to be satisfied.

### Running parameters

We choose our parameter settings (gene content threshold, condition content threshold, motif content threshold) such that the seed module consists of at least four genes (that is, four independent transcription units or non-operon genes) that share at least one motif and 50 conditions. We chose these thresholds as they were the best trade-off between sensitivity (coverage of known interactions in RegulonDB) and novelty (number of new predictions amongst the total number of predictions). For a more detailed description of the parameters and an analysis of the parameter sensitivity, see Additional data file 7. For more detailed biological analysis we selected the first 150 modules from our prioritization list. Modules further down in the list were mostly redundant with previously selected modules.

### Benchmarking with RegulonDB and novel interactions

For genes that are organized into operons, usually only the promoter region of the first operon gene contains a motif instance. Because in RegulonDB the direct interaction between a regulator and a target gene is derived from the presence of an experimentally verified motif instance, only the interaction between a regulator and the first operon gene is reported. RegulonDB contains information on 736 such interactions [3]. Therefore, when comparing the interactions inferred by DISTILLER with the direct interactions in RegulonDB, we only consider those genes inferred by DISTILLER that have the motif instance in their promoter region. All direct interactions inferred by DISTILLER that are not direct interactions according to RegulonDB are considered novel. Some of these interactions might have been reported in the recent literature, but are not yet covered by RegulonDB.

### Experimental validation

Predicted regulatory interactions were experimentally validated *in vivo *using ChIP-qPCR [46]. In total, 11 predicted targets for FNR were selected for experimental validation. As we wanted to test both reliable and less reliable predictions of DISTILLER, we choose the predicted target genes accordingly. In addition, positive controls were necessary: we chose two genes that are known FNR targets and that were identified both in our modules as well as in a recent ChIP-chip study [24].

The conditions that were chosen for the experimental validation were among the conditions selected by DISTILLER (conditions testing differences between aerobic and anaerobic conditions). From all the variants on aerobic-anaerobic shifts, we picked those conditions that were similar to the ones used by Grainger *et al*. [24] as our two positive controls also tested positive under these conditions in their original experiment (Additional data file 8).

### Static versus condition-dependent combinatorial regulation

To compare the level of static combinatorial regulation present in RegulonDB with the level of combinatorial regulation obtained by additionally taking into account expression data, we applied DISTILLER to one data set only, that is, an interaction matrix containing the known motif-gene interactions from RegulonDB. From this analysis, which does not take into account expression constraints, we counted the number of genes found in modules that were regulated by at least two regulators (a gene was counted more than once if it appeared in multiple modules). This number was compared with a similar figure obtained from the co-expression-constrained modules (see Results). The same procedure was followed for the analysis of non-combinatorial modules. The default gene content threshold was used for all analyses mentioned above (see 'Running parameters' above).

### Conditional dependency of the network

All arrays were grouped into 15 conditional categories assigned by manual curation. For each module, the enrichment of its conditions for each of the functional categories was calculated by means of the hypergeometric distribution. Subsequently, for each regulator we selected the corresponding modules, and their enrichments for the conditional categories were combined using Fisher's method [47]. This results in a *P*-value for each combination of a regulator and conditional category. Strong enrichment of one module for a particular category or enrichment of multiple modules belonging to one regulator for the same conditional category can yield significant *P*-values.

### Comparison with other methods

We compared our results on regulator-target interactions for *E. coli *with those identified by Ernst *et al*. [17] and Faith *et al*. [14] by using both methods on our data sources. Although SAMBA [20] could theoretically be used in a setup similar to the one used in this paper, we did not include it in our current work as we already exhaustively tested it in a previous study [19].

For comparison of the DISTILLER interactions with the interactions inferred by CLR and SEREND on the one hand and the interactions of RegulonDB on the other, only interactions between a regulator and the first operon gene were taken into account. For comparison with CLR and SEREND, we only compared the interactions inferred for those 67 regulators for which a binding site is described in RegulonDB. For composite regulators consisting of more subunits - for example, FlhDC [48] - Faith *et al*. [14] report in their initial study the results for each subunit of such composite regulators separately (all interactions for subunit 1 (FlhC) and for subunit 2 (FlhD)), while in our analysis we treated the composite regulators as single entities. Therefore, we corrected the counts reported by their software for these kinds of regulators to make results comparable. Prior to applying CLR, genes and conditions for which too many missing values were present in the expression data had to be deleted from the data set since CLR cannot handle missing values. An alternative comparison with the results obtained by applying CLR to our microarray compendium data set with different thresholds for their z scores was performed (Additional data file 4).

SEREND needs three data sources as input: a list of known regulator-target interactions, regulatory motif data and expression data. The list of known regulator-target interactions was derived from RegulonDB: if a motif instance in the promoter region of a gene was experimentally confirmed, this regulator-gene combination was added to the list of known interactions. This list corresponds to the list of known motif interactions that was also used as input for DISTILLER. Similar to what the authors of SEREND did in the original work, we added the remaining genes of the operons (operon genes are assumed to be regulated by the same regulator as the first gene of the operon) to this list for SEREND. The regulatory motif data used by DISTILLER were transformed to -log(*P*-value), as suggested by the authors of SEREND (personal communication). In addition, the score of the first gene of the operon was copied to the remaining genes of the operon.

SEREND [17] assigns a prioritization score for all the predictions per regulator (the highest scoring prediction is the best), but does not describe a statistical way to select the number of reliable interactions per regulator. For the comparison with DISTILLER, we chose an arbitrary threshold to end up with a defined number of predictions per regulator. We used the threshold defined in the original work of Ernst *et al*. [17]; that is, for each transcription factor we selected the same number of best-scoring predicted targets as there were targets already described for that regulator.

## Abbreviations

ChIP: chromatin immunoprecipitation; ChIP-chip: chromatin immunoprecipitation on chip; CLR: context of likelihood relatedness; CRP: cAMP-receptor protein; FNR: fumarate and nitrate reductase regulator; H-NS: heat-stable nucleoid-structuring protein; IHF: integration host factor; qPCR: quantitative PCR; SEREND: Semi-supervised Regulatory Network Discoverer.

## Authors' contributions

KL, TD, KE and KM designed the study, performed the analyses and wrote the paper. KL collected and KE normalized the microarrays. TDB developed the data integration framework. BDM and JC gave useful comments and critically read the manuscript. SDK, IT, GS, ADW and JV performed the ChIP followed by qPCR of the novel FNR targets. All authors read and approved the final manuscript.

## Additional data files

The following additional data are available with the online version of this paper: a table that provides information on the gene, condition and motif content of all 150 modules that were inferred by DISTILLER (Additional data file 1); a description of how the benchmarking with RegulonDB was performed and that also provides information on the number of interactions from RegulonDB and the number of novel interactions that were identified by DISTILLER (Additional data file 2); a more detailed description of Figure 1, that is, the condition dependency of the regulatory modules (Additional data file 3); a more detailed comparison of DISTILLER and CLR (Additional data file 4); a comparison of CLR, SEREND and DISTILLER with five available ChIP-chip experiments (Additional data file 5); a description of the content of the microarray compendium and the normalization of the microarrays in detail (Additional data file 6); an explanation of the DISTILLER algorithm and its parameter settings (Additional data file 7); extra information on how the predicted FNR targets were experimentally validated (Additional data file 8).

## Supplementary Material

Additional data file 1 Gene, condition and motif content of all 150 modules that were inferred by DISTILLER. Click here for file

Additional data file 2 Description of how the benchmarking with RegulonDB was performed and information on the number of interactions from RegulonDB and the number of novel interactions that were identified by DISTILLER. Click here for file

Additional data file 3 A more detailed description of Figure 1. Click here for file

Additional data file 4 Detailed comparison of DISTILLER and CLR. Click here for file

Additional data file 5 Comparison of CLR, SEREND and DISTILLER with five available ChIP-chip experiments. Click here for file

Additional data file 6 The microarray compendium and the normalization of the microarrays in detail. Click here for file

Additional data file 7 The DISTILLER algorithm and its parameter settings. Click here for file

Additional data file 8 How the predicted FNR targets were experimentally validated. Click here for file
