# Supplementary material for: DISTILLER: a data integration framework to reveal condition dependency of complex regulons in Escherichia coli
Source: Genome Biol. 2009 Mar 6;10(3):R27. doi: 10.1186/gb-2009-10-3-r27 (PMC2690998; doi:10.1186/gb-2009-10-3-r27)
Supplement: Additional data file 1 — Gene, condition and motif content of all 150 modules that were inferred by DISTILLER. [file gb-2009-10-3-r27-S1.pdf]

ADDITIONAL DATA FILE 1: Modules inferred by DISTILLER

DISTILLER is a data integration framework that searches for condition-dependent transcriptional modules by combining expression data with information on the direct interaction between a regulator and its corresponding target genes. In this study, we applied DISTILLER to simultaneously analyze two complementary data sources: a novel cross-platform expression compendium consisting of 870 *E. coli* microarrays and a regulatory motif compendium consisting of both predicted and experimentally verified motif instances. Table S1 describes the 150 statistically most significant modules recovered by DISTILLER.

The functional enrichment was calculated for the genes in each module. Figure S1 shows the distribution of the top 3 p-values for each module. From this figure it seems that most modules a good functional enrichment.

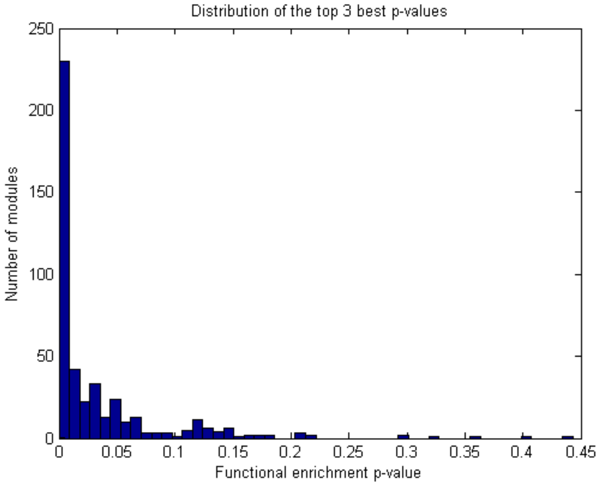

Figure S1: Distribution of the top 3 best p-value. The 3 best functional enrichment p-values for each module were selected. The distribution of these p-values shows that at a threshold of 0.05, 70% of all top 3 chosen categories were significantly enriched.

Table S1: Description of the different modules that were identified by DISTILLER. For each module following information is available: the motifs that are present in the promoter region of the genes (Motifs), the genes that were identified during the seed discovery step (Seed Genes), the genes after the seed extension step (Genes), the top 3 of Ecocyc functional categories in which the genes of the module are enriched and the corresponding p-value (Functional enrichment), the number of conditions that were identified for each condition category (Conditions).

**Module 1**	
Motifs	FNR IHF NarL
Seed Genes	b0904 b1223 b1224 b3365 b4070
Extended seed Genes	b0904 b1223 b1224 b1225 b1226 b3365 b4070
Functional enrichment	Metabolism(0), anaerobic respiration(7.52e-07), electron acceptors(1.1e-05),
Conditions	DNA(2), aerobic__anaerobic(38), amino_acids(2), antibiotic__toxic_agent(5), carbon_source(16), diauxic_shift(12), growth(3), pH(2), unknown(1),

**Module 10**	
Motifs	ArgR
Seed Genes	b0032 b0273 b0860 b3172 b3359 b3958 b4254
Extended seed Genes	b0032 b0033 b0273 b0860 b2818 b3172 b3359 b3958 b3959 b3960 b4254
Functional enrichment	Metabolism(0), amino acids(0), cytoplasm(0.000543),
Conditions	Cu(1), DNA(13), aerobic__anaerobic(29), amino_acids(15), antibiotic__toxic_agent(9), carbon_source(45), diauxic_shift(27), growth(2), heat_cold_stress(1), pH(4), sigma_factor(2), unknown(1),

**Module 100**	
Motifs	CRP Mlc
Seed Genes	b1387 b1817 b3417 b3418
Extended seed Genes	b1387 b1817 b3417 b3418
Functional enrichment	Metabolism(0), Carbon compounds(1.49e-05), Carbon Utilization(7.64e-05),
Conditions	DNA(7), Fe(2), aerobic__anaerobic(8), amino_acids(7), antibiotic__toxic_agent(10), carbon_source(9), diauxic_shift(30), growth(1), heat_cold_stress(1), nitrosating_agent(3), pH(2), sigma_factor(3), unknown(2),

**Module 101**	
Motifs	CpxR
Seed Genes	b3363 b3912 b4143 b4219
Extended seed Genes	b3363 b3912 b4143 b4219
Functional enrichment	Cell Processes(7.33e-05), protein related(0.00182), Information transfer(0.0345),

Conditions	DNA(8), aerobic__anaerobic(4), amino_acids(10), carbon_source(13), diauxic_shift(17), growth(6), heat_cold_stress(2), pH(1), sigma_factor(1),

**Module 102**	
Motifs	LexA
Seed Genes	b0685 b0779 b3813 b4058
Extended seed Genes	b0685 b0779 b3813 b4058
Functional enrichment	Cell Processes(0), DNA related(9.81e-07), Information transfer(0.00224),
Conditions	Biofilm(1), DNA(16), aerobic__anaerobic(4), amino_acids(9), carbon_source(26), diauxic_shift(8), growth(1), heat_cold_stress(1), pH(7), sigma_factor(2),

**Module 103**	
Motifs	LexA
Seed Genes	b0060 b0226 b0779 b2009
Extended seed Genes	b0019 b0060 b0226 b0779 b2009
Functional enrichment	Cell Processes(0), DNA related(3.03e-08), protection(0.000301),
Conditions	DNA(8), Fe(4), aerobic__anaerobic(14), amino_acids(2), antibiotic__toxic_agent(1), carbon_source(21), diauxic_shift(44), pH(1), sigma_factor(3), unknown(3),

**Module 104**	
Motifs	PhoP
Seed Genes	b0557 b1608 b1641 b4006
Extended seed Genes	b0557 b1608 b1641 b4006
Functional enrichment	type of regulation(0.0639), regulation(0.0646), cytoplasm(0.22),
Conditions	Biofilm(1), Cu(1), DNA(8), Fe(2), aerobic__anaerobic(14), amino_acids(8), antibiotic__toxic_agent(7), carbon_source(20), diauxic_shift(17), growth(1), heat_cold_stress(1), nitrosating_agent(1), pH(1), sigma_factor(4), unknown(1),

**Module 105**	
Motifs	PhoP
Seed Genes	b0677 b1608 b1826 b3207
Extended seed Genes	b0677 b1608 b1826 b3207
Functional enrichment	type of regulation(0.0124), regulation(0.0126), Central intermediary metabolism(0.143),
Conditions	Cu(1), DNA(4), Fe(2), aerobic__anaerobic(15), amino_acids(3), antibiotic__toxic_agent(6), carbon_source(10), diauxic_shift(9), growth(1), nitrosating_agent(1), pH(14), sigma_factor(2), unknown(2),

**Module 106**	
Motifs	FNR

Seed Genes	b1003 b1376 b1902 b3417
Extended seed Genes	b0643 b1002 b1003 b1004 b1376 b1902 b3417
Functional enrichment	Metabolism(0.017), amino acids(0.0608), unknown(0.0689),
Conditions	Biofilm(1), DNA(17), Fe(1), aerobic__anaerobic(17), amino_acids(1), antibiotic__toxic_agent(5), carbon_source(28), diauxic_shift(20), sigma_factor(1), unknown(2),

**Module 107**	
Motifs	Mlc
Seed Genes	b1594 b3862 b3884 b3900
Extended seed Genes	b1594 b3862 b3884 b3900
Functional enrichment	regulation(2.54e-12), genetic unit regulated(0.000237), RNA related(0.00636),
Conditions	DNA(3), aerobic__anaerobic(6), carbon_source(5), diauxic_shift(18), growth(1), heat_cold_stress(1), pH(6), sigma_factor(1),

**Module 108**	
Motifs	Mlc
Seed Genes	b0426 b0796 b1817 b3147
Extended seed Genes	b0426 b0796 b1817 b3147
Functional enrichment	group translocators(0.0234), cytoplasm(0.0505), Carbon compounds(0.121),
Conditions	Cu(1), DNA(8), aerobic__anaerobic(14), antibiotic__toxic_agent(1), carbon_source(8), diauxic_shift(12), growth(1), nitrosating_agent(2), pH(1),

**Module 109**	
Motifs	Lrp
Seed Genes	b2905 b3616 b3617 b4129
Extended seed Genes	b2905 b3616 b3617 b4129
Functional enrichment	Metabolism(0), amino acids(1.42e-05), Central intermediary metabolism(0.00155),
Conditions	Biofilm(5), DNA(17), aerobic__anaerobic(19), amino_acids(3), antibiotic__toxic_agent(7), carbon_source(12), diauxic_shift(10), growth(2), heat_cold_stress(1), nitrosating_agent(2), pH(4), sigma_factor(4), unknown(1),

**Module 11**	
Motifs	MetJ
Seed Genes	b0199 b2942 b3939 b3941 b4013
Extended seed Genes	b0199 b2942 b3939 b3941 b4013

Functional enrichment	Metabolism(0), amino acids(3.52e-05), Central intermediary metabolism(0.00365),
Conditions	Biofilm(2), Cu(2), DNA(12), Fe(1), aerobic__anaerobic(15), amino_acids(12), antibiotic__toxic_agent(11), carbon_source(56), diauxic_shift(31), nitrosating_agent(3), pH(3),

**Module 110**	
Motifs	Lrp
Seed Genes	b0077 b0907 b2215 b4376
Extended seed Genes	b0077 b0907 b2215 b4376
Functional enrichment	amino acids(0.0014), outer membrane(0.0635), periplasm(0.114),
Conditions	DNA(3), aerobic__anaerobic(15), amino_acids(14), antibiotic__toxic_agent(3), carbon_source(9), diauxic_shift(17), growth(5), heat_cold_stress(2), nitrosating_agent(1), pH(12), sigma_factor(2),

**Module 111**	
Motifs	Fis
Seed Genes	b1101 b1109 b2231 b2927
Extended seed Genes	b1101 b1109 b2231 b2927
Functional enrichment	Metabolism(0), primary active transporters(0.0159), electron donors(0.039),
Conditions	DNA(8), aerobic__anaerobic(9), amino_acids(6), antibiotic__toxic_agent(10), carbon_source(18), diauxic_shift(40), growth(1), heat_cold_stress(2), nitrosating_agent(1), pH(4), sigma_factor(2),

**Module 112**	
Motifs	Fis
Seed Genes	b0779 b2231 b2821 b3699
Extended seed Genes	b0779 b2231 b2821 b3699
Functional enrichment	Information transfer(2.48e-12), DNA related(0.000123), Cell Processes(0.0455),
Conditions	DNA(19), Fe(2), aerobic__anaerobic(6), antibiotic__toxic_agent(6), carbon_source(27), diauxic_shift(2), pH(20), sigma_factor(4), unknown(5),

**Module 113**	
Motifs	Fis
Seed Genes	b2296 b2398 b3365 b4070
Extended seed Genes	b2296 b2398 b3365 b4070 b4073
Functional enrichment	Metabolism(0), anaerobic respiration(0.000342), Central intermediary metabolism(0.00365),
Conditions	Biofilm(1), Cu(1), DNA(5), Fe(3), aerobic__anaerobic(41), antibiotic__toxic_agent(2), carbon_source(25), diauxic_shift(5), growth(2), nitrosating_agent(1),

**Module 114**	

Motifs	CRP
Seed Genes	b0598 b1805 b2151 b2659 b4035 b4139
Extended seed Genes	b0064 b0268 b0598 b0880 b1002 b1189 b1190 b1415 b1440 b1441 b1612 b1805 b1900 b1901 b1976 b2148 b2149 b2150 b2151 b2536 b2597 b2659 b2799 b2800 b2844 b2869 b2964 b2980 b3253 b3403 b3418 b3426 b3453 b3528 b3588 b3748 b3749 b3750 b3751 b3882 b3926 b3927 b4003 b4035 b4036 b4037 b4139 b4189 b4227 b4228 b4238 b4239 b4240 b4485
Functional enrichment	Metabolism(0), Carbon compounds(1.24e-12), Carbon Utilization(2.01e-11),
Conditions	DNA(2), carbon_source(4), diauxic_shift(34), growth(2),

**Module 115**	
Motifs	CRP
Seed Genes	b0064 b2150 b2535 b2844 b2980 b3588 b4069
Extended seed Genes	b0064 b0162 b0598 b1002 b1189 b1190 b1391 b1397 b1398 b1415 b1440 b1441 b1513 b1516 b1805 b2149 b2150 b2151 b2344 b2535 b2536 b2659 b2660 b2844 b2980 b3453 b3528 b3588 b3927 b4003 b4067 b4069 b4119 b4189 b4216 b4227 b4228 b4485
Functional enrichment	Metabolism(0), Carbon Utilization(2.15e-09), Carbon compounds(1.23e-08),
Conditions	DNA(3), aerobic__anaerobic(5), antibiotic__toxic_agent(1), carbon_source(1), diauxic_shift(41), growth(1),

**Module 116**	
Motifs	CRP
Seed Genes	b0953 b1493 b2597 b3049 b3517
Extended seed Genes	b0485 b0486 b0953 b1492 b1493 b2097 b2597 b3049 b3509 b3510 b3517
Functional enrichment	amino acids(0.000283), Metabolism(0.0135), ribosomes(0.0158),
Conditions	DNA(13), Fe(3), aerobic__anaerobic(28), amino_acids(4), antibiotic__toxic_agent(2), carbon_source(42), diauxic_shift(39), growth(1), pH(8), sigma_factor(3),

**Module 117**	
Motifs	CRP
Seed Genes	b2775 b2841 b2870 b3452 b3666
Extended seed Genes	b2715 b2775 b2841 b2870 b3452 b3666 b3683
Functional enrichment	transport(1.71e-05), group translocators(0.00198), Metabolism(0.00222),
Conditions	DNA(3), aerobic__anaerobic(6), amino_acids(3), antibiotic__toxic_agent(2), carbon_source(4), diauxic_shift(6), growth(2), nitrosating_agent(1), pH(11), sigma_factor(6),

**Module 118**	
Motifs	MetJ
Seed Genes	b2942 b3828 b3829 b3941

Extended seed Genes	b2942 b3828 b3829 b3941
Functional enrichment	regulation(0.000157), Central intermediary metabolism(0.00155), type of regulation(0.00505),
Conditions	Biofilm(3), Cu(2), DNA(16), Fe(1), aerobic__anaerobic(11), amino_acids(2), antibiotic__toxic_agent(4), carbon_source(41), diauxic_shift(13), growth(1), heat_cold_stress(3), nitrosating_agent(7), pH(6), unknown(2),

**Module 119**	
Motifs	NarL
Seed Genes	b0894 b1224 b2997 b4125
Extended seed Genes	b0894 b0895 b1224 b2997 b4125
Functional enrichment	Metabolism(0), anaerobic respiration(3.78e-08), electron acceptors(3.16e-06),
Conditions	Biofilm(7), DNA(6), Fe(1), aerobic__anaerobic(20), antibiotic__toxic_agent(4), carbon_source(21), diauxic_shift(6), growth(3), heat_cold_stress(1), nitrosating_agent(1), unknown(1),

**Module 12**	
Motifs	CRP CytR
Seed Genes	b2143 b2964 b3831 b4381
Extended seed Genes	b2143 b2964 b3831 b4381
Functional enrichment	Central intermediary metabolism(3.02e-05), Metabolism(0.017), inner membrane(0.443),
Conditions	Biofilm(4), DNA(9), aerobic__anaerobic(10), amino_acids(14), antibiotic__toxic_agent(10), carbon_source(30), diauxic_shift(33), growth(2), heat_cold_stress(1), nitrosating_agent(1), pH(9), sigma_factor(4),

**Module 120**	
Motifs	Fur
Seed Genes	b0432 b0721 b3005 b3006 b3908
Extended seed Genes	b0429 b0430 b0431 b0432 b0721 b0722 b0723 b3005 b3006 b3908
Functional enrichment	Metabolism(0), aerobic respiration(4.17e-12), electron acceptors(4.14e-07),
Conditions	Biofilm(2), Cu(1), DNA(9), Fe(6), aerobic__anaerobic(30), amino_acids(5), antibiotic__toxic_agent(2), carbon_source(30), diauxic_shift(5), growth(1), nitrosating_agent(3), sigma_factor(2),

**Module 121**	
Motifs	Fur
Seed Genes	b2392 b3008 b3440 b3441
Extended seed Genes	b2392 b3008 b3440 b3441
Functional enrichment	amino acids(0.0309), protection(0.0626), Cell Processes(0.177),

Conditions	Cu(1), DNA(5), aerobic__anaerobic(5), antibiotic__toxic_agent(3), carbon_source(2), diauxic_shift(6), growth(4), heat_cold_stress(3), nitrosating_agent(2), pH(9),

**Module 122**	
Motifs	Fur
Seed Genes	b0590 b0591 b1374 b2393
Extended seed Genes	b0590 b0591 b1374 b2393
Functional enrichment	inner membrane(0.009), transport(0.0146), membrane(0.0152),
Conditions	Biofilm(1), DNA(6), aerobic__anaerobic(8), amino_acids(1), antibiotic__toxic_agent(8), carbon_source(6), diauxic_shift(10), pH(2), sigma_factor(2), unknown(2),

**Module 123**	
Motifs	IHF
Seed Genes	b0280 b0553 b0720 b1892
Extended seed Genes	b0280 b0553 b0720 b1892
Functional enrichment	TCA cycle(0.0178), Extrachromosomal(0.0296), flagella(0.0409),
Conditions	Biofilm(4), Cu(2), DNA(9), Fe(1), aerobic__anaerobic(2), amino_acids(4), antibiotic__toxic_agent(5), carbon_source(36), diauxic_shift(22), growth(1), heat_cold_stress(1), nitrosating_agent(1), pH(2), sigma_factor(4),

**Module 124**	
Motifs	IHF
Seed Genes	b0125 b1344 b1656 b3554
Extended seed Genes	b0125 b1344 b1656 b3554
Functional enrichment	cytoplasm(0.0505), protection(0.0626), Central intermediary metabolism(0.143),
Conditions	Biofilm(2), DNA(3), Fe(2), aerobic__anaerobic(4), amino_acids(14), antibiotic__toxic_agent(5), carbon_source(9), diauxic_shift(16), growth(1), heat_cold_stress(1), nitrosating_agent(1), pH(11), sigma_factor(1), unknown(1),

**Module 125**	
Motifs	IHF
Seed Genes	b0125 b1109 b2435 b3650
Extended seed Genes	b0125 b1109 b2435 b3650
Functional enrichment	Metabolism(0), primary active transporters(0.0159), Central intermediary metabolism(0.03),
Conditions	Biofilm(1), DNA(9), aerobic__anaerobic(8), amino_acids(1), antibiotic__toxic_agent(7), carbon_source(15), diauxic_shift(52), growth(1), heat_cold_stress(2), pH(3),

**Module 126**	

Motifs	IHF
Seed Genes	b0098 b3766 b4138 b4314
Extended seed Genes	b0098 b3766 b4138 b4314
Functional enrichment	pilus(0.0399), amino acids(0.0608), transport(0.124),
Conditions	Biofilm(2), DNA(3), Fe(1), aerobic__anaerobic(11), antibiotic__toxic_agent(7), carbon_source(11), diauxic_shift(24), heat_cold_stress(3), nitrosating_agent(1), pH(3), sigma_factor(3), unknown(1),

**Module 127**	
Motifs	IHF
Seed Genes	b1338 b1387 b2710 b3421
Extended seed Genes	b1338 b1387 b2710 b3421
Functional enrichment	Carbon compounds(0.00389), Carbon Utilization(0.00878), Metabolism(0.131),
Conditions	Biofilm(2), Cu(1), Fe(3), aerobic__anaerobic(4), carbon_source(14), diauxic_shift(15), pH(1), unknown(1),

**Module 128**	
Motifs	ArcA
Seed Genes	b0007 b0280 b1109 b4058
Extended seed Genes	b0007 b0280 b1109 b4058
Functional enrichment	primary active transporters(0.0159), electron donors(0.039), Cell Processes(0.0455),
Conditions	DNA(3), aerobic__anaerobic(5), amino_acids(5), carbon_source(4), diauxic_shift(4), growth(2), sigma_factor(6), unknown(3),

**Module 129**	
Motifs	ArcA
Seed Genes	b1101 b1109 b1132 b3237
Extended seed Genes	b1101 b1109 b1132 b3237
Functional enrichment	Metabolism(0), primary active transporters(0.0159), electron donors(0.039),
Conditions	Biofilm(1), DNA(12), aerobic__anaerobic(17), amino_acids(4), antibiotic__toxic_agent(5), carbon_source(7), diauxic_shift(32), growth(1), heat_cold_stress(1), nitrosating_agent(1), pH(2), sigma_factor(2),

**Module 13**	
Motifs	CRP Mlc
Seed Genes	b0678 b1617 b1817 b3417
Extended seed Genes	b0678 b1617 b1817 b3417

Functional enrichment	Metabolism(0), Carbon compounds(0.000919), Carbon Utilization(0.00305),
Conditions	DNA(16), Fe(1), aerobic__anaerobic(12), antibiotic__toxic_agent(5), carbon_source(18), diauxic_shift(25), growth(2), nitrosating_agent(1), sigma_factor(2),

**Module 130**	
Motifs	ArcA
Seed Genes	b0155 b0854 b0972 b2380
Extended seed Genes	b0155 b0854 b0972 b2380
Functional enrichment	Metabolism(0), amines(0.0363), electron donors(0.039),
Conditions	DNA(3), Fe(1), aerobic__anaerobic(8), amino_acids(1), antibiotic__toxic_agent(2), carbon_source(8), diauxic_shift(16), heat_cold_stress(1), pH(12), sigma_factor(2),

**Module 131**	
Motifs	ArcA
Seed Genes	b0318 b3025 b3128 b3603
Extended seed Genes	b0318 b3025 b3128 b3603
Functional enrichment	Carbon compounds(0.0112), Carbon Utilization(0.0247), Metabolism(0.298),
Conditions	DNA(2), aerobic__anaerobic(2), amino_acids(3), antibiotic__toxic_agent(5), carbon_source(1), diauxic_shift(20), growth(2), nitrosating_agent(3), pH(1), sigma_factor(4), unknown(1),

**Module 132**	
Motifs	FlhDC
Seed Genes	b1880 b1904 b1937 b2625
Extended seed Genes	b1880 b1904 b1937 b2625
Functional enrichment	flagella(0.000313), Cell Structure(0.011), flagellum(0.0252),
Conditions	Biofilm(3), DNA(3), Fe(1), aerobic__anaerobic(17), amino_acids(8), antibiotic__toxic_agent(4), carbon_source(22), diauxic_shift(11), nitrosating_agent(1), pH(3), sigma_factor(4), unknown(1),

**Module 133**	
Motifs	NtrC
Seed Genes	b0470 b2309 b3869 b3871
Extended seed Genes	b0470 b2309 b3869 b3871
Functional enrichment	type of regulation(0.0345), regulation(0.0349), amino acids(0.046),
Conditions	Biofilm(2), DNA(5), Fe(1), aerobic__anaerobic(14), amino_acids(1), antibiotic__toxic_agent(5), carbon_source(14), diauxic_shift(18), growth(3), heat_cold_stress(2), nitrosating_agent(2), pH(8), sigma_factor(5), unknown(2),

Module 134	
Motifs	NtrC
Seed Genes	b1012 b1988 b3073 b3870
Extended seed Genes	b1012 b1988 b3073 b3869 b3870
Functional enrichment	Metabolism(0), regulation(2.54e-12), type of regulation(0.000153),
Conditions	Cu(1), DNA(4), aerobic__anaerobic(20), amino_acids(2), antibiotic__toxic_agent(9), carbon_source(7), diauxic_shift(16), nitrosating_agent(1), pH(3), sigma_factor(4),

Module 135	
Motifs	CRP
Seed Genes	b0347 b0953 b2096 b3132 b3528
Extended seed Genes	b0347 b0953 b1415 b2092 b2093 b2094 b2095 b2096 b2980 b3132 b3403 b3528 b4069 b4227
Functional enrichment	Metabolism(0), Carbon Utilization(2.24e-11), Carbon compounds(6.13e-10),
Conditions	DNA(1), aerobic__anaerobic(2), antibiotic__toxic_agent(1), carbon_source(2), diauxic_shift(41), growth(1),

Module 136	
Motifs	CRP
Seed Genes	b0064 b2146 b2702 b2869 b4299
Extended seed Genes	b0064 b0598 b0880 b1002 b1901 b1976 b2146 b2147 b2702 b2705 b2800 b2869 b2964 b3403 b3566 b4216 b4227 b4299
Functional enrichment	Carbon compounds(0.00011), periplasm(0.000589), Carbon Utilization(0.00102),
Conditions	Biofilm(2), DNA(4), aerobic__anaerobic(2), amino_acids(3), carbon_source(27), diauxic_shift(13), growth(2), unknown(1),

Module 137	
Motifs	CRP
Seed Genes	b0268 b2151 b3093 b4003 b4321
Extended seed Genes	b0268 b2151 b3093 b4003 b4321
Functional enrichment	regulation(0.000713), type of regulation(0.0116), membrane(0.0334),
Conditions	Fe(1), aerobic__anaerobic(3), amino_acids(2), antibiotic__toxic_agent(2), carbon_source(9), diauxic_shift(20), growth(1), nitrosating_agent(1), sigma_factor(4),

Module 138	
Motifs	CRP
Seed Genes	b0598 b1189 b3748 b4118 b4354

Extended seed Genes	b0598 b1189 b1190 b1814 b2535 b2869 b3239 b3748 b4118 b4354
Functional enrichment	amino acids(3.57e-06), Metabolism(0.00438), adaptations(0.0252),
Conditions	aerobic__anaerobic(1), carbon_source(9), diauxic_shift(22), growth(1), pH(7),

**Module 139**	
Motifs	CRP
Seed Genes	b0763 b1139 b3405 b3947
Extended seed Genes	b0763 b1139 b3405 b3947
Functional enrichment	regulation(0.000157), type of regulation(0.00505), periplasm(0.114),
Conditions	Biofilm(1), DNA(4), Fe(2), aerobic__anaerobic(9), amino_acids(9), antibiotic__toxic_agent(4), carbon_source(7), diauxic_shift(14), growth(2), nitrosating_agent(1), pH(1), sigma_factor(9), unknown(1),

**Module 14**	
Motifs	CRP Mlc
Seed Genes	b1101 b1498 b1594 b2415
Extended seed Genes	b1101 b1498 b1594 b2415
Functional enrichment	regulation(0.000157), genetic unit regulated(0.00887), Metabolism(0.017),
Conditions	Biofilm(2), DNA(4), amino_acids(1), antibiotic__toxic_agent(2), carbon_source(13), diauxic_shift(8), growth(3), heat_cold_stress(1), nitrosating_agent(1), sigma_factor(6),

**Module 140**	
Motifs	CRP
Seed Genes	b0822 b0898 b2942 b3830
Extended seed Genes	b0822 b0898 b2942 b3830
Functional enrichment	Central intermediary metabolism(0.0158), unknown(0.0521), Metabolism(0.298),
Conditions	Biofilm(2), Cu(1), DNA(3), Fe(2), aerobic__anaerobic(12), amino_acids(5), antibiotic__toxic_agent(1), carbon_source(10), diauxic_shift(2), heat_cold_stress(1), pH(1), sigma_factor(2), unknown(2),

**Module 141**	
Motifs	CRP
Seed Genes	b0125 b1704 b3363 b3806
Extended seed Genes	b0125 b1704 b3363 b3806
Functional enrichment	amino acids(0.0608), posttranscriptional(0.0866), cytoplasm(0.22),
Conditions	Biofilm(2), DNA(8), Fe(2), aerobic__anaerobic(12), antibiotic__toxic_agent(7), carbon_source(20),

	diauxic_shift(26), growth(3), heat_cold_stress(1), nitrosating_agent(2), pH(10), sigma_factor(6), unknown(1),

**Module 142**	
Motifs	CRP
Seed Genes	b1612 b1778 b3225 b3672
Extended seed Genes	b1612 b1778 b3225 b3672
Functional enrichment	TCA cycle(0.0178), amino acids(0.0608), adaptations(0.122),
Conditions	Biofilm(4), Cu(1), DNA(5), Fe(1), aerobic__anaerobic(23), amino_acids(3), antibiotic__toxic_agent(5), carbon_source(13), diauxic_shift(16), nitrosating_agent(1), pH(1), sigma_factor(3),

**Module 143**	
Motifs	CRP
Seed Genes	b0113 b1685 b2913 b3357
Extended seed Genes	b0113 b1685 b2913 b3357
Functional enrichment	regulation(0), type of regulation(0.00138), genetic unit regulated(0.00455),
Conditions	Biofilm(3), Cu(6), DNA(6), aerobic__anaerobic(13), amino_acids(1), antibiotic__toxic_agent(2), carbon_source(5), diauxic_shift(8), growth(1), heat_cold_stress(1), nitrosating_agent(2), pH(6), sigma_factor(8), unknown(5),

**Module 144**	
Motifs	CRP
Seed Genes	b0041 b1040 b2801 b3904
Extended seed Genes	b0041 b1040 b2801 b3904
Functional enrichment	Carbon compounds(0.000919), Carbon Utilization(0.00305), Metabolism(0.138),
Conditions	Biofilm(1), Cu(1), DNA(10), Fe(3), aerobic__anaerobic(4), amino_acids(5), antibiotic__toxic_agent(9), carbon_source(3), diauxic_shift(14), growth(11), pH(2), sigma_factor(5), unknown(3),

**Module 145**	
Motifs	CRP
Seed Genes	b0331 b0459 b0957 b1188
Extended seed Genes	b0331 b0459 b0957 b1188
Functional enrichment	Carbon Utilization(0.00082), Carbon compounds(0.0112), fatty acids(0.0225),
Conditions	DNA(4), Fe(1), aerobic__anaerobic(6), amino_acids(3), antibiotic__toxic_agent(5), carbon_source(2), diauxic_shift(6), pH(1), sigma_factor(6), unknown(2),

**Module 146**	
Motifs	CRP

Seed Genes	b2393 b2943 b3356 b3870
Extended seed Genes	b2393 b2943 b3356 b3870
Functional enrichment	Metabolism(0), amino acids(0.046), inner membrane(0.0504),
Conditions	Biofilm(4), DNA(2), Fe(1), aerobic__anaerobic(3), antibiotic__toxic_agent(3), carbon_source(4), diauxic_shift(8), heat_cold_stress(1), nitrosating_agent(1), sigma_factor(7), unknown(2),

**Module 147**	
Motifs	CRP
Seed Genes	b1182 b2344 b4208 b4267
Extended seed Genes	b1182 b2344 b4208 b4267
Functional enrichment	extracellular(0.00188), fatty acids(0.0298), Carbon Utilization(0.0463),
Conditions	Biofilm(1), DNA(1), Fe(1), aerobic__anaerobic(8), amino_acids(1), antibiotic__toxic_agent(2), carbon_source(8), diauxic_shift(31), growth(2), heat_cold_stress(1), nitrosating_agent(1), sigma_factor(2),

**Module 148**	
Motifs	CRP
Seed Genes	b1255 b2508 b2509 b3238
Extended seed Genes	b1255 b2507 b2508 b2509 b3238
Functional enrichment	Metabolism(0), Central intermediary metabolism(0.03), DNA related(0.121),
Conditions	Biofilm(1), DNA(11), Fe(1), aerobic__anaerobic(7), antibiotic__toxic_agent(2), carbon_source(11), diauxic_shift(36), growth(6), heat_cold_stress(1), pH(1), sigma_factor(1),

**Module 149**	
Motifs	CRP
Seed Genes	b1138 b3575 b3905 b3909
Extended seed Genes	b1138 b3575 b3905 b3909
Functional enrichment	Carbon compounds(0.0214), Carbon Utilization(0.0463), regulation(0.0646),
Conditions	DNA(7), Fe(1), aerobic__anaerobic(8), amino_acids(1), carbon_source(2), diauxic_shift(12), growth(1), heat_cold_stress(1), nitrosating_agent(1), pH(3), unknown(2),

**Module 15**	
Motifs	CRP Fis
Seed Genes	b0440 b1241 b4000 b4142
Extended seed Genes	b0440 b1241 b1779 b4000 b4142
Functional	protein related(0.00429), cytoplasm(0.0104), glycolysis(0.021),

enrichment	
Conditions	DNA(15), Fe(2), aerobic__anaerobic(24), amino_acids(13), carbon_source(14), diauxic_shift(8), growth(3), heat_cold_stress(2), nitrosating_agent(2), pH(13), sigma_factor(2), unknown(1),

**Module 150**	
Motifs	CRP
Seed Genes	b3707 b4195 b4268 b4322
Extended seed Genes	b3707 b4195 b4268 b4322
Functional enrichment	Carbon compounds(0.0214), group translocators(0.0463), Carbon Utilization(0.0463),
Conditions	Cu(3), DNA(14), Fe(2), aerobic__anaerobic(11), amino_acids(5), antibiotic__toxic_agent(3), carbon_source(24), diauxic_shift(14), growth(3), heat_cold_stress(3), sigma_factor(3), unknown(2),

**Module 16**	
Motifs	CRP IHF
Seed Genes	b0124 b3908 b4014 b4069
Extended seed Genes	b0124 b3908 b4014 b4069
Functional enrichment	Metabolism(0), Central intermediary metabolism(0.00155), Carbon Utilization(0.00305),
Conditions	DNA(11), Fe(1), aerobic__anaerobic(28), amino_acids(2), antibiotic__toxic_agent(3), carbon_source(17), diauxic_shift(2), growth(1), nitrosating_agent(1), pH(5), sigma_factor(10), unknown(1),

**Module 17**	
Motifs	ArcA FNR
Seed Genes	b0432 b1324 b1415 b2288
Extended seed Genes	b0431 b0432 b1324 b1415 b2288
Functional enrichment	Metabolism(0), primary active transporters(5.32e-07), aerobic respiration(1.61e-05),
Conditions	Biofilm(9), Cu(1), DNA(21), aerobic__anaerobic(34), antibiotic__toxic_agent(6), carbon_source(3), diauxic_shift(6), growth(8), heat_cold_stress(1), nitrosating_agent(3), pH(17), sigma_factor(1), unknown(1),

**Module 18**	
Motifs	ArcA CRP
Seed Genes	b1388 b2844 b3907 b4240
Extended seed Genes	b1388 b2844 b3907 b4240
Functional enrichment	Carbon compounds(0.0112), Carbon Utilization(0.0247), group translocators(0.0349),
Conditions	Cu(1), DNA(6), Fe(1), aerobic__anaerobic(2), amino_acids(4), antibiotic__toxic_agent(6), carbon_source(7), diauxic_shift(13), growth(1), nitrosating_agent(1), sigma_factor(3),

Module 19	
Motifs	NtrC
Seed Genes	b0811 b1748 b1988 b3869 b3870
Extended seed Genes	b0811 b1746 b1747 b1748 b1988 b3869 b3870
Functional enrichment	amino acids(1.6e-08), regulation(0.000302), Metabolism(0.0008),
Conditions	aerobic__anaerobic(13), amino_acids(1), antibiotic__toxic_agent(6), carbon_source(3), diauxic_shift(11), growth(2),

Module 2	
Motifs	FNR NarL NarP
Seed Genes	b0873 b1474 b2208 b4070
Extended seed Genes	b0873 b1474 b2204 b2208 b4070
Functional enrichment	Metabolism(0), anaerobic respiration(5.7e-06), electron carriers(0.00015),
Conditions	Biofilm(2), DNA(5), Fe(1), aerobic__anaerobic(27), antibiotic__toxic_agent(5), carbon_source(12), diauxic_shift(12), growth(3), heat_cold_stress(2), nitrosating_agent(1), pH(1), sigma_factor(3), unknown(2),

Module 20	
Motifs	CRP FNR
Seed Genes	b0034 b1901 b2957 b4139
Extended seed Genes	b0034 b1901 b2957 b4139
Functional enrichment	Metabolism(0), amino acids(1.42e-05), periplasm(0.00505),
Conditions	Biofilm(4), Cu(1), DNA(5), Fe(1), aerobic__anaerobic(24), amino_acids(1), antibiotic__toxic_agent(5), carbon_source(18), diauxic_shift(8), heat_cold_stress(2), nitrosating_agent(4), pH(9), unknown(1),

Module 21	
Motifs	Fur
Seed Genes	b0150 b1018 b1252 b1451 b3005 b4367
Extended seed Genes	b0150 b0468 b0591 b0593 b1018 b1252 b1451 b1452 b2155 b2211 b3005 b3006 b3070 b3908 b4293 b4367
Functional enrichment	transport(6.43e-05), Extrachromosomal(0.00246), membrane(0.00356),
Conditions	Fe(7), aerobic__anaerobic(36), amino_acids(5), carbon_source(23), diauxic_shift(4), nitrosating_agent(1), unknown(1),

Module 22	
Motifs	MalT
Seed Genes	b0403 b3417 b4034 b4035

Extended seed Genes	b0403 b3417 b4034 b4035 b4036 b4037
Functional enrichment	Metabolism(0), Carbon compounds(5.22e-06), Carbon Utilization(3.93e-05),
Conditions	DNA(8), Fe(4), aerobic__anaerobic(15), amino_acids(2), antibiotic__toxic_agent(3), carbon_source(29), diauxic_shift(10), growth(2), heat_cold_stress(3), nitrosating_agent(4), pH(10), sigma_factor(2),

**Module 23**	
Motifs	CysB
Seed Genes	b2414 b2425 b2752 b2764 b4311
Extended seed Genes	b1287 b2414 b2422 b2423 b2424 b2425 b2750 b2751 b2752 b2762 b2763 b2764 b4311
Functional enrichment	Metabolism(1.35e-05), transport(0.0859), amino acids(0.159),
Conditions	Biofilm(2), DNA(16), aerobic__anaerobic(17), amino_acids(5), antibiotic__toxic_agent(8), carbon_source(12), diauxic_shift(51), growth(1), heat_cold_stress(2), nitrosating_agent(2), pH(2), sigma_factor(6),

**Module 24**	
Motifs	PurR
Seed Genes	b0032 b0336 b1062 b2499 b2682
Extended seed Genes	b0032 b0033 b0336 b0337 b0945 b1062 b2313 b2499 b2508 b2682
Functional enrichment	Metabolism(0), amino acids(0.0099), Central intermediary metabolism(0.0331),
Conditions	DNA(5), aerobic__anaerobic(12), amino_acids(4), antibiotic__toxic_agent(4), carbon_source(38), diauxic_shift(29), growth(5), heat_cold_stress(1), nitrosating_agent(2), pH(18), sigma_factor(2),

**Module 25**	
Motifs	CRP FNR IHF
Seed Genes	b0904 b2240 b3118 b3365
Extended seed Genes	b0904 b2240 b3118 b3365
Functional enrichment	Metabolism(0), anaerobic respiration(0.0063), aerobic respiration(0.0472),
Conditions	Biofilm(1), Cu(1), DNA(7), aerobic__anaerobic(13), antibiotic__toxic_agent(2), carbon_source(7), diauxic_shift(11), pH(6),

**Module 26**	
Motifs	CRP NagC
Seed Genes	b0678 b0679 b1738 b1817
Extended seed Genes	b0678 b0679 b1738 b1817 b1818
Functional enrichment	Metabolism(0), group translocators(8.46e-08), Carbon Utilization(0.000353),

Conditions	Biofilm(1), Cu(1), DNA(16), Fe(1), aerobic__anaerobic(15), amino_acids(14), antibiotic__toxic_agent(8), carbon_source(22), diauxic_shift(26), growth(1), heat_cold_stress(2), pH(5), sigma_factor(1), unknown(1),

**Module 27**	
Motifs	CpxR
Seed Genes	b0161 b0970 b3912 b3913 b3914 b4484
Extended seed Genes	b0161 b0970 b3912 b3913 b3914 b4484
Functional enrichment	Cell Processes(7.33e-05), periplasm(0.00505), type of regulation(0.00505),
Conditions	Cu(3), DNA(5), aerobic__anaerobic(17), amino_acids(1), antibiotic__toxic_agent(3), carbon_source(25), diauxic_shift(10), growth(4), nitrosating_agent(3), pH(11),

**Module 28**	
Motifs	ArcA CRP
Seed Genes	b0432 b0721 b1276 b4376
Extended seed Genes	b0430 b0432 b0720 b0721 b0722 b0723 b0726 b0728 b0729 b1276 b4376
Functional enrichment	Metabolism(0), TCA cycle(1.59e-13), aerobic respiration(8.99e-08),
Conditions	Biofilm(1), Cu(1), DNA(11), aerobic__anaerobic(52), antibiotic__toxic_agent(2), carbon_source(13), diauxic_shift(1), nitrosating_agent(2), pH(17), sigma_factor(7), unknown(3),

**Module 29**	
Motifs	FNR IHF NarL
Seed Genes	b1224 b2288 b2710 b4070
Extended seed Genes	b1224 b2288 b2710 b4070
Functional enrichment	Metabolism(0), anaerobic respiration(3.59e-05), electron acceptors(0.000144),
Conditions	Cu(1), DNA(1), aerobic__anaerobic(9), amino_acids(4), antibiotic__toxic_agent(3), carbon_source(11), diauxic_shift(19), pH(15), sigma_factor(2), unknown(2),

**Module 3**	
Motifs	FlhDC
Seed Genes	b1072 b1073 b1880 b1904 b1922 b1924 b1938 b1944
Extended seed Genes	b1070 b1071 b1072 b1073 b1074 b1075 b1076 b1077 b1078 b1079 b1080 b1081 b1879 b1880 b1904 b1921 b1922 b1924 b1925 b1926 b1937 b1938 b1939 b1940 b1942 b1943 b1944 b1945 b1946 b1947 b1948 b1949
Functional enrichment	Cell Structure(0), flagella(1.54e-64), flagellum(3.93e-58),
Conditions	DNA(3), aerobic__anaerobic(27), amino_acids(2), antibiotic__toxic_agent(1), carbon_source(7), diauxic_shift(2), growth(1), heat_cold_stress(1), nitrosating_agent(1), pH(4),

**Module 30**	

Motifs	CRP Fis
Seed Genes	b0118 b3072 b4069 b4355
Extended seed Genes	b0118 b3072 b4069 b4355
Functional enrichment	Metabolism(0), Carbon Utilization(7.64e-05), fatty acids(0.000328),
Conditions	DNA(10), aerobic__anaerobic(22), amino_acids(3), antibiotic__toxic_agent(2), carbon_source(10), diauxic_shift(1), growth(1), pH(5), sigma_factor(5), unknown(2),

**Module 31**	
Motifs	CueR
Seed Genes	b0123 b0209 b0484 b0781
Extended seed Genes	b0123 b0209 b0484 b0781
Functional enrichment	Cell Processes(0.0242), periplasm(0.0865), protection(0.0925),
Conditions	Biofilm(2), DNA(4), Fe(3), aerobic__anaerobic(19), amino_acids(6), antibiotic__toxic_agent(1), carbon_source(25), diauxic_shift(8), heat_cold_stress(2), nitrosating_agent(2), pH(2), sigma_factor(2), unknown(3),

**Module 32**	
Motifs	SoxS
Seed Genes	b0683 b0684 b0850 b2159
Extended seed Genes	b0683 b0684 b0850 b2159
Functional enrichment	electron donors(0.0294), regulation(0.0349), protection(0.0925),
Conditions	Cu(6), DNA(17), Fe(1), aerobic__anaerobic(23), amino_acids(9), antibiotic__toxic_agent(6), carbon_source(16), diauxic_shift(17), growth(1), heat_cold_stress(3), nitrosating_agent(4), pH(8), sigma_factor(1), unknown(1),

**Module 33**	
Motifs	LexA
Seed Genes	b0799 b0958 b1848 b2616 b2699
Extended seed Genes	b0799 b0958 b1848 b2616 b2699
Functional enrichment	Cell Processes(0), Information transfer(0), DNA related(0),
Conditions	Cu(1), DNA(29), Fe(1), aerobic__anaerobic(5), amino_acids(10), antibiotic__toxic_agent(1), carbon_source(5), heat_cold_stress(2), sigma_factor(1),

**Module 34**	
Motifs	FadR
Seed Genes	b0954 b1805 b2344 b3846
Extended seed	b0954 b1805 b2344 b3846

Genes	
Functional enrichment	Metabolism(0), fatty acids(1.55e-06), Carbon Utilization(0.00305),
Conditions	Biofilm(2), Cu(1), DNA(4), Fe(1), aerobic__anaerobic(11), antibiotic__toxic_agent(5), carbon_source(15), diauxic_shift(38), growth(1), heat_cold_stress(1), nitrosating_agent(1), pH(3), sigma_factor(7), unknown(1),

**Module 35**	
Motifs	Fis
Seed Genes	b0405 b0835 b1274 b3260 b3965
Extended seed Genes	b0405 b0469 b0835 b1274 b1344 b1876 b3260 b3261 b3965
Functional enrichment	Information transfer(0), RNA related(0.000428), cytoplasm(0.000722),
Conditions	Cu(1), DNA(5), aerobic__anaerobic(12), amino_acids(3), antibiotic__toxic_agent(7), carbon_source(16), diauxic_shift(27), growth(1), heat_cold_stress(1), pH(2),

**Module 36**	
Motifs	MarA
Seed Genes	b3506 b3510 b4107 b4177
Extended seed Genes	b3506 b3509 b3510 b4107 b4177
Functional enrichment	Cell Processes(0.000795), adaptations(0.003), outer membrane(0.048),
Conditions	DNA(7), Fe(1), aerobic__anaerobic(31), amino_acids(4), antibiotic__toxic_agent(4), carbon_source(15), diauxic_shift(2), growth(1), nitrosating_agent(2), pH(9), sigma_factor(4), unknown(1),

**Module 37**	
Motifs	RcsB
Seed Genes	b0419 b1481 b1482 b4377
Extended seed Genes	b0419 b1481 b1482 b4377
Functional enrichment	adaptations(0.0631), Cell Processes(0.177), type of regulation(0.211),
Conditions	Biofilm(4), Cu(1), DNA(5), aerobic__anaerobic(20), amino_acids(1), antibiotic__toxic_agent(7), carbon_source(19), diauxic_shift(12), nitrosating_agent(1), pH(2), sigma_factor(2),

**Module 38**	
Motifs	CysB
Seed Genes	b0058 b0427 b1627 b2375
Extended seed Genes	b0058 b0059 b0427 b1627 b2375
Functional enrichment	RNA related(0.0739), protection(0.121), membrane(0.127),

Conditions	DNA(2), Fe(1), aerobic__anaerobic(16), amino_acids(11), antibiotic__toxic_agent(6), carbon_source(2), diauxic_shift(5), growth(1), heat_cold_stress(2), pH(6),

**Module 39**	
Motifs	PurR
Seed Genes	b1132 b1658 b3654 b3823
Extended seed Genes	b1132 b1658 b3654 b3823
Functional enrichment	inner membrane(0.009), membrane(0.0152), Central intermediary metabolism(0.03),
Conditions	Biofilm(1), Cu(4), DNA(6), Fe(3), aerobic__anaerobic(11), antibiotic__toxic_agent(2), carbon_source(11), diauxic_shift(26), heat_cold_stress(2), nitrosating_agent(3), pH(4), sigma_factor(1), unknown(1),

**Module 4**	
Motifs	MarA SoxS
Seed Genes	b0871 b2237 b3908 b3924
Extended seed Genes	b0871 b2237 b3908 b3924
Functional enrichment	Cell Processes(0.00296), protection(0.00578), Metabolism(0.017),
Conditions	Biofilm(6), Cu(5), DNA(11), Fe(1), aerobic__anaerobic(21), amino_acids(2), antibiotic__toxic_agent(10), carbon_source(7), diauxic_shift(2), growth(1), heat_cold_stress(1), pH(5), sigma_factor(5), unknown(3),

**Module 40**	
Motifs	GadE
Seed Genes	b3490 b3512 b3517 b4326
Extended seed Genes	b3490 b3512 b3517 b4326
Functional enrichment	amino acids(0.0309), unknown(0.035), Metabolism(0.131),
Conditions	Biofilm(2), Cu(1), DNA(12), aerobic__anaerobic(19), amino_acids(3), antibiotic__toxic_agent(5), carbon_source(15), diauxic_shift(23), growth(4), heat_cold_stress(2), pH(11), sigma_factor(2), unknown(1),

**Module 41**	
Motifs	TyrR
Seed Genes	b0112 b0406 b0754 b2601
Extended seed Genes	b0112 b0406 b0754 b2601
Functional enrichment	amino acids(1.42e-05), Metabolism(0.138), RNA related(0.402),
Conditions	Biofilm(2), Cu(2), DNA(10), Fe(2), aerobic__anaerobic(18), antibiotic__toxic_agent(2), carbon_source(28), diauxic_shift(23), growth(2), heat_cold_stress(1), nitrosating_agent(3), pH(10), sigma_factor(4),

**Module 42**	
Motifs	FruR
Seed Genes	b1136 b3357 b3916 b4014
Extended seed Genes	b1136 b3357 b3916 b4014
Functional enrichment	regulation(0.00515), Metabolism(0.017), TCA cycle(0.0178),
Conditions	Biofilm(1), Cu(3), DNA(3), Fe(2), aerobic__anaerobic(15), amino_acids(9), antibiotic__toxic_agent(12), carbon_source(5), diauxic_shift(3), growth(1), heat_cold_stress(2), nitrosating_agent(4), pH(12), sigma_factor(1),

**Module 43**	
Motifs	FruR
Seed Genes	b2169 b2388 b2415 b2927
Extended seed Genes	b2168 b2169 b2388 b2415 b2927
Functional enrichment	Metabolism(0), Carbon compounds(0.00219), Carbon Utilization(0.00709),
Conditions	Biofilm(2), DNA(8), Fe(1), aerobic__anaerobic(26), amino_acids(2), antibiotic__toxic_agent(2), carbon_source(11), diauxic_shift(7), growth(1), heat_cold_stress(2), nitrosating_agent(1), pH(7), sigma_factor(4),

**Module 44**	
Motifs	PhoB
Seed Genes	b2720 b2884 b3211 b3728 b4464
Extended seed Genes	b2720 b2884 b3211 b3728 b4464
Functional enrichment	fermentation(0.0225), Metabolism(0.0472), transport(0.0693),
Conditions	Cu(3), DNA(9), aerobic__anaerobic(7), amino_acids(1), antibiotic__toxic_agent(1), carbon_source(4), diauxic_shift(10), growth(1), nitrosating_agent(1), sigma_factor(2), unknown(1),

**Module 45**	
Motifs	FNR
Seed Genes	b0894 b1256 b1541 b2727 b4238
Extended seed Genes	b0894 b0895 b1256 b1541 b1593 b2726 b2727 b2728 b2729 b4238 b4380
Functional enrichment	Metabolism(0), anaerobic respiration(4.28e-06), electron acceptors(0.00169),
Conditions	Biofilm(1), DNA(2), aerobic__anaerobic(34), amino_acids(2), antibiotic__toxic_agent(3), carbon_source(39), diauxic_shift(13), growth(2), heat_cold_stress(1), sigma_factor(1),

**Module 46**	
Motifs	OxyR
Seed Genes	b0683 b0812 b1688 b4387

Extended seed Genes	b0683 b0812 b1688 b4387
Functional enrichment	regulation(0.0646), adaptations(0.122), genetic unit regulated(0.149),
Conditions	DNA(4), aerobic__anaerobic(2), amino_acids(10), antibiotic__toxic_agent(5), carbon_source(3), diauxic_shift(8), pH(2), sigma_factor(3),

**Module 47**	
Motifs	OmpR
Seed Genes	b0553 b0598 b0811 b1892
Extended seed Genes	b0553 b0598 b0811 b1892
Functional enrichment	flagella(0.0409), Cell Processes(0.0455), amino acids(0.0608),
Conditions	Biofilm(5), DNA(10), Fe(3), aerobic__anaerobic(8), amino_acids(5), antibiotic__toxic_agent(7), carbon_source(24), diauxic_shift(12), growth(3), heat_cold_stress(1), nitrosating_agent(2), pH(3), sigma_factor(5), unknown(1),

**Module 48**	
Motifs	IscR
Seed Genes	b1684 b1706 b2531 b3414
Extended seed Genes	b1684 b1706 b2531 b3414
Functional enrichment	regulation(0.0349), genetic unit regulated(0.114), Central intermediary metabolism(0.207),
Conditions	Biofilm(5), Cu(3), DNA(8), Fe(2), aerobic__anaerobic(19), amino_acids(5), antibiotic__toxic_agent(9), carbon_source(17), diauxic_shift(14), growth(2), heat_cold_stress(2), pH(5), sigma_factor(4),

**Module 49**	
Motifs	FNR NarL
Seed Genes	b0887 b0904 b1474 b4123
Extended seed Genes	b0887 b0904 b1474 b4123
Functional enrichment	inner membrane(0.009), transport(0.0146), membrane(0.0152),
Conditions	DNA(3), Fe(6), aerobic__anaerobic(15), amino_acids(1), antibiotic__toxic_agent(3), carbon_source(3), diauxic_shift(33), growth(1), heat_cold_stress(3), nitrosating_agent(1), pH(2),

**Module 5**	
Motifs	Fur
Seed Genes	b0584 b0585 b0591 b0592 b0593 b1102 b2155 b2673 b4291 b4293
Extended seed Genes	b0150 b0583 b0584 b0585 b0586 b0591 b0592 b0593 b0594 b0595 b0596 b0597 b0805 b1102 b1252 b1452 b1682 b1684 b1705 b2155 b2673 b2674 b2675 b2676 b3005 b3006 b3070 b3337 b4290 b4291 b4292 b4293 b4367
Functional enrichment	adaptations(1.72e-11), Cell Processes(4.19e-06), transport(5.01e-05),

Conditions	Cu(1), Fe(4), aerobic__anaerobic(29), carbon_source(9), diauxic_shift(6), pH(1), sigma_factor(1),

**Module 50**	
Motifs	FNR NarL
Seed Genes	b0827 b1222 b1224 b3365
Extended seed Genes	b0827 b1222 b1224 b3365
Functional enrichment	Metabolism(0), anaerobic respiration(0.00014), electron acceptors(0.028),
Conditions	Biofilm(1), DNA(5), aerobic__anaerobic(15), amino_acids(2), antibiotic__toxic_agent(4), carbon_source(7), diauxic_shift(12), growth(3), heat_cold_stress(3), nitrosating_agent(2), pH(6), sigma_factor(4),

**Module 51**	
Motifs	NsrR
Seed Genes	b1426 b2552 b2732 b4209
Extended seed Genes	b1426 b2552 b2732 b4209
Functional enrichment	Central intermediary metabolism(0.0745), Metabolism(0.362),
Conditions	DNA(2), Fe(1), aerobic__anaerobic(18), amino_acids(1), antibiotic__toxic_agent(5), carbon_source(3), diauxic_shift(7), nitrosating_agent(3), pH(1),

**Module 52**	
Motifs	CpxR
Seed Genes	b0441 b2215 b3057 b4160
Extended seed Genes	b0441 b2215 b3057 b4160
Functional enrichment	membrane(0.0152), phospholipid(0.0187), Cell Structure(0.0366),
Conditions	Biofilm(1), DNA(9), Fe(1), aerobic__anaerobic(10), amino_acids(4), antibiotic__toxic_agent(1), carbon_source(10), diauxic_shift(28), growth(1), heat_cold_stress(1), pH(10), sigma_factor(5), unknown(1),

**Module 53**	
Motifs	PhoP
Seed Genes	b0841 b1505 b1608 b4242
Extended seed Genes	b0841 b1505 b1608 b4242
Functional enrichment	membrane(0.0152), Cell Structure(0.0366), outer membrane(0.0635),
Conditions	DNA(13), aerobic__anaerobic(21), amino_acids(9), antibiotic__toxic_agent(8), carbon_source(10), diauxic_shift(5), growth(5), nitrosating_agent(1), sigma_factor(2), unknown(2),

**Module 54**	
Motifs	ArgR

Seed Genes	b0295 b0674 b2669 b3957
Extended seed Genes	b0295 b0674 b2669 b3957
Functional enrichment	amino acids(3.6e-06), cytoplasm(0.0113), protein related(0.0176),
Conditions	DNA(3), aerobic__anaerobic(2), amino_acids(4), antibiotic__toxic_agent(1), carbon_source(8), diauxic_shift(22), pH(8), sigma_factor(3), unknown(1),

**Module 55**	
Motifs	NarL
Seed Genes	b0904 b0972 b1241 b2997
Extended seed Genes	b0904 b0972 b1241 b2997
Functional enrichment	Metabolism(0), electron donors(0.000567), anaerobic respiration(0.0063),
Conditions	DNA(6), Fe(5), aerobic__anaerobic(54), amino_acids(4), antibiotic__toxic_agent(4), carbon_source(9), diauxic_shift(23), growth(3), nitrosating_agent(1), pH(1), sigma_factor(3), unknown(1),

**Module 56**	
Motifs	CRP Fis
Seed Genes	b0116 b0118 b3073 b4111
Extended seed Genes	b0116 b0118 b3073 b4111
Functional enrichment	Metabolism(0), aerobic respiration(0.000837), anaerobic respiration(0.0063),
Conditions	DNA(11), aerobic__anaerobic(38), antibiotic__toxic_agent(3), carbon_source(13), diauxic_shift(1), growth(4), heat_cold_stress(1), pH(9), sigma_factor(2),

**Module 57**	
Motifs	NagC
Seed Genes	b0681 b2774 b3411 b3427
Extended seed Genes	b0681 b2774 b3411 b3427
Functional enrichment	transposon related(0.0323), outer membrane(0.0323), Extrachromosomal(0.143),
Conditions	DNA(5), aerobic__anaerobic(4), amino_acids(6), carbon_source(8), diauxic_shift(16), pH(9), sigma_factor(2), unknown(1),

**Module 58**	
Motifs	NagC
Seed Genes	b0394 b2112 b3688 b3746
Extended seed Genes	b0394 b2112 b3688 b3746
Functional	unknown(0.035), Carbon compounds(0.121), Carbon Utilization(0.179),

enrichment	
Conditions	Biofilm(4), Cu(2), DNA(5), Fe(5), aerobic__anaerobic(18), carbon_source(19), diauxic_shift(40), growth(1), heat_cold_stress(1), nitrosating_agent(1), pH(7), sigma_factor(2), unknown(1),

**Module 59**	
Motifs	NagC
Seed Genes	b2443 b2846 b3717 b4311
Extended seed Genes	b2443 b2846 b3717 b4311
Functional enrichment	Extrachromosomal(0.074),
Conditions	Biofilm(1), DNA(11), Fe(2), aerobic__anaerobic(13), amino_acids(8), antibiotic__toxic_agent(5), carbon_source(15), diauxic_shift(12), growth(7), heat_cold_stress(2), unknown(1),

**Module 6**	
Motifs	CRP DeoR
Seed Genes	b0683 b4380 b4381 b4383
Extended seed Genes	b0683 b4380 b4381 b4383
Functional enrichment	Central intermediary metabolism(0.0158), regulation(0.0349), genetic unit regulated(0.114),
Conditions	Biofilm(2), DNA(9), Fe(1), aerobic__anaerobic(5), amino_acids(10), antibiotic__toxic_agent(8), carbon_source(14), diauxic_shift(30), growth(2), heat_cold_stress(5), nitrosating_agent(1), pH(1), sigma_factor(4), unknown(2),

**Module 60**	
Motifs	NagC
Seed Genes	b0191 b1266 b1654 b2987
Extended seed Genes	b0191 b1266 b1654 b2987
Functional enrichment	inner membrane(0.136), transport(0.161), membrane(0.163),
Conditions	Biofilm(3), DNA(2), aerobic__anaerobic(7), amino_acids(4), antibiotic__toxic_agent(5), carbon_source(5), diauxic_shift(12), pH(1),

**Module 61**	
Motifs	LexA
Seed Genes	b1061 b1861 b3065 b4043
Extended seed Genes	b1061 b1861 b3065 b4043
Functional enrichment	Information transfer(2.48e-12), DNA related(9.81e-07), Cell Processes(0.00296),
Conditions	Biofilm(1), Cu(2), DNA(15), Fe(1), aerobic__anaerobic(17), amino_acids(4), carbon_source(15), diauxic_shift(16), pH(10), unknown(4),

**Module 62**	

Motifs	LexA
Seed Genes	b0231 b0779 b3645 b3832
Extended seed Genes	b0231 b0779 b3645 b3832
Functional enrichment	DNA related(3.15e-05), Cell Processes(0.000795), Information transfer(0.0103),
Conditions	Biofilm(2), DNA(27), Fe(1), aerobic__anaerobic(16), amino_acids(11), antibiotic__toxic_agent(1), carbon_source(10), diauxic_shift(2), growth(2), heat_cold_stress(2), nitrosating_agent(1), unknown(2),

**Module 63**	
Motifs	LexA
Seed Genes	b0227 b3391 b3501 b3673
Extended seed Genes	b0227 b3391 b3501 b3673
Functional enrichment	protection(0.00295), Cell Processes(0.0242), regulation(0.0349),
Conditions	Biofilm(3), DNA(9), aerobic__anaerobic(18), amino_acids(4), antibiotic__toxic_agent(6), carbon_source(44), diauxic_shift(16), growth(1), heat_cold_stress(1), pH(2), unknown(2),

**Module 64**	
Motifs	Lrp
Seed Genes	b0889 b0891 b2669 b3460
Extended seed Genes	b0889 b0891 b2669 b3460
Functional enrichment	regulation(0.000157), amino acids(0.0014), type of regulation(0.00505),
Conditions	Biofilm(2), Cu(2), DNA(3), Fe(1), aerobic__anaerobic(6), amino_acids(3), antibiotic__toxic_agent(3), carbon_source(7), diauxic_shift(25), heat_cold_stress(1), nitrosating_agent(5), pH(14), sigma_factor(2), unknown(2),

**Module 65**	
Motifs	Lrp
Seed Genes	b0077 b0922 b1243 b4314
Extended seed Genes	b0077 b0922 b1243 b4314
Functional enrichment	pilus(0.0399), murein(0.0545), amino acids(0.0608),
Conditions	Biofilm(2), DNA(6), aerobic__anaerobic(6), carbon_source(15), diauxic_shift(33), nitrosating_agent(2), pH(6), sigma_factor(4), unknown(2),

**Module 66**	
Motifs	Lrp
Seed Genes	b0966 b1189 b1660 b3617
Extended seed Genes	b0966 b1189 b1660 b3617

Functional enrichment	amino acids(0.000708), Metabolism(0.0472), Central intermediary metabolism(0.207),
Conditions	Biofilm(1), DNA(12), Fe(1), aerobic__anaerobic(15), amino_acids(2), antibiotic__toxic_agent(6), carbon_source(10), diauxic_shift(24), heat_cold_stress(2), nitrosating_agent(1), sigma_factor(1),

**Module 67**	
Motifs	Fis
Seed Genes	b2549 b2550 b3000 b3588
Extended seed Genes	b2549 b2550 b3000 b3588
Functional enrichment	Carbon compounds(0.121), Metabolism(0.131), Central intermediary metabolism(0.143),
Conditions	Biofilm(1), Cu(1), DNA(4), Fe(1), aerobic__anaerobic(9), amino_acids(1), antibiotic__toxic_agent(2), carbon_source(10), diauxic_shift(20), growth(1), heat_cold_stress(1), pH(1), sigma_factor(1), unknown(2),

**Module 68**	
Motifs	Fis
Seed Genes	b2288 b2296 b2508 b4108
Extended seed Genes	b2288 b2296 b2508 b4108
Functional enrichment	Metabolism(0), Central intermediary metabolism(3.02e-05), primary active transporters(0.0159),
Conditions	DNA(12), Fe(1), aerobic__anaerobic(13), amino_acids(5), antibiotic__toxic_agent(2), carbon_source(2), diauxic_shift(11), growth(2), heat_cold_stress(2), nitrosating_agent(2), pH(5), sigma_factor(5),

**Module 69**	
Motifs	Fis
Seed Genes	b1185 b2231 b2784 b3813
Extended seed Genes	b1185 b2231 b2784 b3813
Functional enrichment	DNA related(0.000123), Information transfer(0.00224), Cell Structure(0.00244),
Conditions	Cu(2), DNA(14), Fe(2), aerobic__anaerobic(16), antibiotic__toxic_agent(2), carbon_source(3), diauxic_shift(30), growth(3), heat_cold_stress(1), pH(4),

**Module 7**	
Motifs	CRP GlpR
Seed Genes	b2240 b2241 b3426 b3927
Extended seed Genes	b2239 b2240 b2241 b2243 b3426 b3926 b3927
Functional enrichment	Metabolism(0), Central intermediary metabolism(1.19e-08), electron donors(3.07e-05),
Conditions	Biofilm(1), DNA(5), Fe(2), aerobic__anaerobic(8), amino_acids(3), antibiotic__toxic_agent(3),

	carbon_source(65), diauxic_shift(14), heat_cold_stress(2), nitrosating_agent(4), pH(3), sigma_factor(2),

**Module 70**	
Motifs	Fur
Seed Genes	b0755 b1684 b2392 b2673 b3006 b4367
Extended seed Genes	b0468 b0584 b0592 b0755 b1252 b1452 b1679 b1680 b1681 b1682 b1683 b1684 b2392 b2673 b2674 b2675 b2676 b3005 b3006 b3337 b4293 b4367
Functional enrichment	Central intermediary metabolism(3.17e-05), Metabolism(0.00017), adaptations(0.000254),
Conditions	Biofilm(1), Cu(3), DNA(1), Fe(4), aerobic__anaerobic(23), carbon_source(18), diauxic_shift(6), nitrosating_agent(1), pH(1), sigma_factor(2),

**Module 71**	
Motifs	CRP Fur
Seed Genes	b0584 b0593 b0683 b0805
Extended seed Genes	b0583 b0584 b0592 b0593 b0594 b0596 b0683 b0805 b2155
Functional enrichment	adaptations(0.00235), membrane(0.00807), outer membrane(0.00872),
Conditions	Cu(4), DNA(8), Fe(1), aerobic__anaerobic(25), amino_acids(8), antibiotic__toxic_agent(1), carbon_source(15), diauxic_shift(7), growth(2), heat_cold_stress(2), pH(1), sigma_factor(4), unknown(4),

**Module 72**	
Motifs	NtrC
Seed Genes	b0450 b1823 b1824 b1988
Extended seed Genes	b0450 b1823 b1824 b1988
Functional enrichment	type of regulation(0), regulation(0), unknown(0.0521),
Conditions	Biofilm(2), Cu(1), DNA(13), Fe(1), aerobic__anaerobic(12), antibiotic__toxic_agent(13), carbon_source(24), diauxic_shift(8), growth(4), heat_cold_stress(2), nitrosating_agent(2), pH(7), sigma_factor(4), unknown(2),

**Module 73**	
Motifs	NtrC
Seed Genes	b3870 b3871 b3973 b4146
Extended seed Genes	b3870 b3871 b3973 b4146
Functional enrichment	regulation(0.0349), amino acids(0.046), Metabolism(0.0472),
Conditions	DNA(20), aerobic__anaerobic(9), amino_acids(1), antibiotic__toxic_agent(8), carbon_source(25), diauxic_shift(29), growth(3), heat_cold_stress(2), nitrosating_agent(1), pH(11), sigma_factor(4), unknown(1),

**Module 74**	

Motifs	IHF
Seed Genes	b0032 b0811 b2508 b3784
Extended seed Genes	b0032 b0432 b0809 b0811 b2507 b2508 b3784
Functional enrichment	Metabolism(0), amino acids(0.00012), primary active transporters(0.0277),
Conditions	DNA(7), Fe(4), aerobic__anaerobic(12), amino_acids(1), antibiotic__toxic_agent(3), carbon_source(12), diauxic_shift(7), growth(1), heat_cold_stress(1), nitrosating_agent(5), pH(5), unknown(1),

**Module 75**	
Motifs	IHF
Seed Genes	b0125 b0754 b0929 b4314
Extended seed Genes	b0125 b0565 b0754 b0929 b3319 b3320 b3321 b3734 b3736 b4314
Functional enrichment	Cell Structure(0.000171), ribosomes(0.00113), protein related(0.00541),
Conditions	Biofilm(1), Cu(1), DNA(4), aerobic__anaerobic(14), amino_acids(5), antibiotic__toxic_agent(3), carbon_source(12), diauxic_shift(38), growth(1), heat_cold_stress(3), nitrosating_agent(1), pH(5), sigma_factor(1), unknown(2),

**Module 76**	
Motifs	IHF
Seed Genes	b1684 b1739 b2215 b3405
Extended seed Genes	b1684 b1739 b2215 b3405
Functional enrichment	regulation(2.54e-12), type of regulation(0.000153), genetic unit regulated(0.00887),
Conditions	Cu(2), DNA(7), Fe(1), aerobic__anaerobic(12), amino_acids(11), antibiotic__toxic_agent(7), carbon_source(11), diauxic_shift(6), growth(1), heat_cold_stress(1), nitrosating_agent(1), pH(4), sigma_factor(2),

**Module 77**	
Motifs	IHF
Seed Genes	b0720 b3073 b3544 b4332
Extended seed Genes	b0720 b3073 b3544 b4332
Functional enrichment	Metabolism(0), amino acids(0.0014), TCA cycle(0.0178),
Conditions	DNA(15), Fe(1), aerobic__anaerobic(19), antibiotic__toxic_agent(2), carbon_source(1), diauxic_shift(17), growth(4), heat_cold_stress(4), nitrosating_agent(4), pH(12), sigma_factor(3), unknown(4),

**Module 78**	
Motifs	IHF
Seed Genes	b0812 b0912 b1712 b4376

Extended seed Genes	b0812 b0912 b1712 b4376
Functional enrichment	Information transfer(0.00224), DNA related(0.00578), adaptations(0.00587),
Conditions	Biofilm(6), Cu(1), DNA(8), Fe(2), aerobic__anaerobic(22), amino_acids(8), antibiotic__toxic_agent(4), carbon_source(34), diauxic_shift(13), growth(1), pH(8), sigma_factor(3),

**Module 79**	
Motifs	IHF
Seed Genes	b0992 b1908 b2725 b2726
Extended seed Genes	b0992 b1908 b2725 b2726
Functional enrichment	regulation(0.0014), fermentation(0.0225), type of regulation(0.0345),
Conditions	DNA(1), Fe(1), aerobic__anaerobic(19), amino_acids(1), antibiotic__toxic_agent(1), carbon_source(11), diauxic_shift(3), growth(3), heat_cold_stress(1), pH(1),

**Module 8**	
Motifs	PurR
Seed Genes	b0523 b2312 b2313 b2476 b2551 b2557 b4006
Extended seed Genes	b0522 b0523 b2312 b2313 b2476 b2551 b2557 b4005 b4006
Functional enrichment	Metabolism(0), cytoplasm(4.6e-05), amino acids(0.132),
Conditions	DNA(4), aerobic__anaerobic(10), amino_acids(12), antibiotic__toxic_agent(6), carbon_source(21), diauxic_shift(18), growth(1), heat_cold_stress(1), pH(8), sigma_factor(2),

**Module 80**	
Motifs	FNR
Seed Genes	b0621 b1674 b2504 b2552
Extended seed Genes	b0621 b1674 b2504 b2552
Functional enrichment	Carbon compounds(0.121), Metabolism(0.131), Central intermediary metabolism(0.143),
Conditions	DNA(4), aerobic__anaerobic(5), amino_acids(14), antibiotic__toxic_agent(1), carbon_source(1), diauxic_shift(5), growth(1), nitrosating_agent(1), pH(1), sigma_factor(4),

**Module 81**	
Motifs	FNR
Seed Genes	b1241 b3516 b3517 b4401
Extended seed Genes	b1241 b3516 b3517 b4401
Functional enrichment	regulation(2.54e-12), type of regulation(0.000153), fermentation(0.0298),

Conditions	Biofilm(4), DNA(4), Fe(5), aerobic__anaerobic(25), amino_acids(2), antibiotic__toxic_agent(7), carbon_source(33), diauxic_shift(32), growth(6), heat_cold_stress(2), pH(6), sigma_factor(3), unknown(2),

**Module 82**	
Motifs	FNR
Seed Genes	b0781 b1210 b2579 b3544
Extended seed Genes	b0781 b1210 b2579 b3544
Functional enrichment	Metabolism(0.017), amino acids(0.0608), periplasm(0.114),
Conditions	Biofilm(1), DNA(4), aerobic__anaerobic(21), antibiotic__toxic_agent(2), carbon_source(6), diauxic_shift(3), heat_cold_stress(1), nitrosating_agent(1), unknown(1),

**Module 83**	
Motifs	FNR
Seed Genes	b0650 b3072 b3476 b4122
Extended seed Genes	b0650 b3072 b3476 b4122
Functional enrichment	Metabolism(0.017), TCA cycle(0.0178), fermentation(0.0298),
Conditions	Biofilm(3), DNA(9), Fe(1), aerobic__anaerobic(12), antibiotic__toxic_agent(11), carbon_source(19), diauxic_shift(14), growth(3), heat_cold_stress(2), nitrosating_agent(1), pH(4), unknown(3),

**Module 84**	
Motifs	FNR
Seed Genes	b1750 b4039 b4279 b4403
Extended seed Genes	b1358 b1750 b4039 b4279 b4326 b4403
Functional enrichment	aerobic respiration(0.0587), transport(0.185), Extrachromosomal(0.319),
Conditions	DNA(1), aerobic__anaerobic(22), antibiotic__toxic_agent(1), diauxic_shift(4), heat_cold_stress(2), nitrosating_agent(1), pH(2), unknown(1),

**Module 85**	
Motifs	FNR
Seed Genes	b0585 b2498 b2580 b4278
Extended seed Genes	b0585 b2498 b2580 b4278
Functional enrichment	Central intermediary metabolism(0.03), transposon related(0.0635), DNA related(0.121),
Conditions	Biofilm(1), DNA(9), Fe(1), aerobic__anaerobic(3), antibiotic__toxic_agent(3), carbon_source(10), diauxic_shift(37), pH(6), sigma_factor(1), unknown(2),

**Module 86**	
Motifs	FNR

Seed Genes	b0113 b1109 b1276 b3519
Extended seed Genes	b0113 b1109 b1276 b3519
Functional enrichment	Metabolism(0), anaerobic respiration(0.0063), primary active transporters(0.0159),
Conditions	Biofilm(1), DNA(4), Fe(1), aerobic__anaerobic(32), amino_acids(6), antibiotic__toxic_agent(1), carbon_source(13), diauxic_shift(3), growth(3), pH(2), sigma_factor(4),

**Module 87**	
Motifs	FNR
Seed Genes	b1902 b2111 b3518 b3942
Extended seed Genes	b1902 b2111 b3518 b3942
Functional enrichment	large molecule carriers(0.039), pilus(0.0399), Cell Processes(0.0455),
Conditions	Biofilm(3), DNA(9), Fe(1), aerobic__anaerobic(16), antibiotic__toxic_agent(4), carbon_source(20), diauxic_shift(4), growth(1), heat_cold_stress(1), nitrosating_agent(3), pH(2), sigma_factor(4),

**Module 88**	
Motifs	FruR
Seed Genes	b2340 b2597 b3403 b4014
Extended seed Genes	b2096 b2340 b2597 b3403 b4014
Functional enrichment	Central intermediary metabolism(0.0476), protein related(0.0527), type of regulation(0.0987),
Conditions	DNA(6), Fe(1), aerobic__anaerobic(6), amino_acids(9), antibiotic__toxic_agent(6), carbon_source(14), diauxic_shift(39), growth(5), heat_cold_stress(1), nitrosating_agent(2), pH(1), sigma_factor(2),

**Module 89**	
Motifs	FruR
Seed Genes	b1241 b3299 b3357 b3956
Extended seed Genes	b1241 b3299 b3357 b3956
Functional enrichment	fermentation(0.000328), regulation(0.00515), TCA cycle(0.0178),
Conditions	Biofilm(3), Cu(1), DNA(2), Fe(1), aerobic__anaerobic(30), amino_acids(8), antibiotic__toxic_agent(1), carbon_source(11), diauxic_shift(14), growth(13), heat_cold_stress(2), nitrosating_agent(6), pH(12), sigma_factor(6), unknown(3),

**Module 9**	
Motifs	ArcA CRP
Seed Genes	b0116 b0118 b0720 b1415 b3236
Extended seed Genes	b0116 b0118 b0720 b0721 b0722 b0723 b0726 b0727 b0728 b0729 b1415 b3236
Functional	Metabolism(0), TCA cycle(6.36e-12), aerobic respiration(1.53e-07),

enrichment	
Conditions	Biofilm(5), DNA(17), aerobic__anaerobic(55), amino_acids(3), antibiotic__toxic_agent(8), carbon_source(31), diauxic_shift(9), growth(9), nitrosating_agent(1), pH(9), sigma_factor(2),

**Module 90**	
Motifs	ArcA
Seed Genes	b1415 b2092 b2537 b2844 b3603 b3846
Extended seed Genes	b0598 b1415 b1853 b2091 b2092 b2093 b2094 b2095 b2096 b2537 b2844 b3603 b3604 b3846
Functional enrichment	Metabolism(6.28e-13), Carbon compounds(7.27e-09), Carbon Utilization(1.05e-08),
Conditions	Biofilm(2), DNA(2), aerobic__anaerobic(3), amino_acids(3), antibiotic__toxic_agent(2), carbon_source(22), diauxic_shift(38), growth(5), heat_cold_stress(1), nitrosating_agent(1),

**Module 91**	
Motifs	ArcA
Seed Genes	b0854 b1136 b2741 b3739
Extended seed Genes	b0854 b1136 b2741 b3739
Functional enrichment	Metabolism(0), adaptations(0.00587), TCA cycle(0.0178),
Conditions	Cu(3), DNA(6), Fe(2), aerobic__anaerobic(15), amino_acids(6), antibiotic__toxic_agent(3), carbon_source(5), diauxic_shift(1), nitrosating_agent(1), pH(6), sigma_factor(1),

**Module 92**	
Motifs	ArcA
Seed Genes	b0040 b2070 b2997 b4328
Extended seed Genes	b0040 b2070 b2997 b4328
Functional enrichment	Metabolism(0), electron donors(0.0294), anaerobic respiration(0.0964),
Conditions	Cu(1), DNA(3), Fe(2), aerobic__anaerobic(14), amino_acids(1), carbon_source(8), diauxic_shift(3), growth(1), heat_cold_stress(2), nitrosating_agent(1), pH(9), sigma_factor(1),

**Module 93**	
Motifs	ArcA
Seed Genes	b0733 b0827 b0904 b2193
Extended seed Genes	b0733 b0827 b0904 b2193
Functional enrichment	regulation(0.00515), Metabolism(0.017), electron acceptors(0.028),
Conditions	Biofilm(2), Cu(1), DNA(9), Fe(1), aerobic__anaerobic(19), amino_acids(12), antibiotic__toxic_agent(9), carbon_source(5), diauxic_shift(15), growth(3), heat_cold_stress(1), nitrosating_agent(1), pH(9), sigma_factor(3), unknown(4),

**Module 94**	

Motifs	ArcA
Seed Genes	b0313 b1109 b2518 b3908
Extended seed Genes	b0313 b0429 b0430 b0432 b1109 b2518 b3908
Functional enrichment	Metabolism(0), aerobic respiration(6.31e-07), primary active transporters(1.85e-06),
Conditions	Biofilm(1), DNA(10), aerobic__anaerobic(34), amino_acids(2), antibiotic__toxic_agent(5), carbon_source(19), diauxic_shift(29), growth(2), nitrosating_agent(2), pH(2), sigma_factor(1), unknown(3),

**Module 95**	
Motifs	PurR
Seed Genes	b1062 b2498 b2508 b2888
Extended seed Genes	b0945 b1062 b2498 b2507 b2508 b2888
Functional enrichment	Metabolism(0), Central intermediary metabolism(0.00691), cytoplasm(0.0257),
Conditions	Biofilm(1), DNA(12), Fe(1), aerobic__anaerobic(17), amino_acids(3), antibiotic__toxic_agent(3), carbon_source(30), diauxic_shift(33), growth(2), heat_cold_stress(2), nitrosating_agent(1), pH(8), sigma_factor(3), unknown(2),

**Module 96**	
Motifs	PurR
Seed Genes	b0032 b0945 b1039 b2905
Extended seed Genes	b0032 b0033 b0945 b1039 b2905
Functional enrichment	Metabolism(0), amino acids(3.52e-05), pilus(0.0497),
Conditions	Biofilm(2), DNA(7), Fe(4), aerobic__anaerobic(8), amino_acids(9), antibiotic__toxic_agent(1), carbon_source(17), diauxic_shift(29), growth(3), heat_cold_stress(1), nitrosating_agent(1), pH(5), sigma_factor(6), unknown(1),

**Module 97**	
Motifs	PurR
Seed Genes	b0411 b1849 b2312 b3714
Extended seed Genes	b0411 b1849 b2312 b3714
Functional enrichment	Extrachromosomal(0.0296), transport(0.124), Metabolism(0.138),
Conditions	DNA(12), Fe(3), aerobic__anaerobic(21), antibiotic__toxic_agent(12), carbon_source(35), diauxic_shift(3), growth(2), pH(4), sigma_factor(2), unknown(1),

**Module 98**	
Motifs	TyrR
Seed Genes	b0112 b1323 b1842 b1894

Extended seed Genes	b0112 b1323 b1842 b1894
Functional enrichment	regulation(0.00515), amino acids(0.0608), transposon related(0.0635),
Conditions	Cu(1), DNA(7), Fe(1), aerobic__anaerobic(11), amino_acids(7), antibiotic__toxic_agent(8), carbon_source(23), diauxic_shift(9), growth(1), pH(8), sigma_factor(3), unknown(1),

**Module 99**	
Motifs	CRP FNR
Seed Genes	b2240 b2241 b2481 b4123
Extended seed Genes	b2240 b2241 b2481 b4123
Functional enrichment	Metabolism(0), anaerobic respiration(0.00014), Central intermediary metabolism(0.03),
Conditions	Biofilm(1), DNA(6), Fe(6), aerobic__anaerobic(8), amino_acids(4), antibiotic__toxic_agent(1), carbon_source(20), diauxic_shift(19), growth(4), heat_cold_stress(1), pH(5), sigma_factor(3), unknown(2),
