# Supplementary material for: DISTILLER: a data integration framework to reveal condition dependency of complex regulons in Escherichia coli
Source: Genome Biol. 2009 Mar 6;10(3):R27. doi: 10.1186/gb-2009-10-3-r27 (PMC2690998; doi:10.1186/gb-2009-10-3-r27)
Supplement: Additional data file 2 — Description of how the benchmarking with RegulonDB was performed and information on the number of interactions from RegulonDB and the number of novel interactions that were identified by DISTILLER. [file gb-2009-10-3-r27-S2.pdf]

ADDITIONAL DATA FILE 2: Benchmarking with RegulonDB and novel predictions

Motif data were integrated with our large scale expression compendium to generate condition-dependent modules. The 150 statistically significant modules recovered by DISTILLER represent 454 of 736 interactions for 67 regulators with known binding sites described in RegulonDB.

In addition to identifying 454 previously described interactions, we predict 278 novel interactions that have not previously been described in RegulonDB. We classified these predictions according to decreasing levels of confidence. Our classification is based on the fact that regulator-target interactions confirm each other if the target genes are co-expressed in the same subset of conditions, i.e. if the target genes belong to the same module. Many of the new predictions correspond to interactions of a known regulator with hypothetical genes, expanding the knowledge on regulons of well studied regulators. Novel interactions from modules that contain at least 50% known interactions can be assumed very reliable (Category I: 157 interactions). Predicted interactions from modules that contain at least one previously known interaction form the second most reliable group (Category II: 118 interactions). Finally, novel interactions from modules that contain no previously known interaction (Category III: 20 interactions) are more interesting but can no further be verified based on existing knowledge.

Table S1 gives an overview of the number of known interactions per regulator that were identified, as well as the number of new, predicted interactions. The large number of predictions for FNR, CRP, ArcA, Fis, and IHF confirms their role as global hubs in the network. For some regulators only the previously described interactions, and/or a few additional ones could be retrieved (e.g. CueR, GlpR). Because we found these regulators active in conditions of the compendium but could not extend these regulons any further, we postulate that these regulators are nearly completely characterized and indeed target only few genes (operons) triggering very specific pathways. Table S2 gives an overview of the novel predictions for each regulator.

Table S1: Interactions recovered by DISTILLER

The number of known and novel interactions for each regulator that were identified by applying DISTILLER on the current motif and microarray compendia is shown. Regulator: a list of all regulators in RegulonDB for which the binding site is known; Modules: the number of modules that were found to be dependent on a specific regulator; Overlap RegulonDB: the number of experimentally confirmed interactions described in RegulonDB that could be detected by DISTILLER; Total new: the number of new interactions not previously described in RegulonDB that were detected by DISTILLER; New cat I: new interactions originating from modules that contain at least 50% known interactions; New cat II: new interactions originating from modules that contain at least 1 known interaction; New cat III: new interactions originating from modules that contain no known interactions.

Regulator	Modules	Overlap RegulonDB	Total new	New cat I	New cat II	New cat III
Ada	0	0 out of 3	0	0	0	0
AgaR	0	0 out of 3	0	0	0	0
AraC	0	0 out of 5	0	0	0	0
ArcA	13	28 out of 35	23	13	12	0
ArgP	0	0 out of 2	0	0	0	0
ArgR	2	7 out of 9	5	2	3	0
CRP	38	111 out of 153	63	49	24	0
CaiF	0	0 out of 2	0	0	0	0
CpxR	3	8 out of 17	5	5	0	0
CueR	1	3 out of 3	1	1	0	0
CysB	2	4 out of 6	6	2	0	4
CytR	1	3 out of 7	1	1	0	0
DcuR	0	0 out of 3	0	0	0	0
DeoR	1	1 out of 2	3	0	3	0
DnaA	0	0 out of 6	0	0	0	0
FNR	19	48 out of 57	25	7	18	0
FadR	1	4 out of 7	0	0	0	0
FhlA	0	0 out of 4	0	0	0	0
Fis	10	21 out of 25	20	12	8	0
FlhDC	2	9 out of 10	5	5	0	0
FruR	4	8 out of 12	7	2	6	0
Fur	7	23 out of 29	15	13	3	0
GadE	1	2 out of 3	2	2	0	0
GalR	0	0 out of 4	0	0	0	0
GalS	0	0 out of 4	0	0	0	0
GcvA	0	0 out of 2	0	0	0	0
GlpR	1	4 out of 4	0	0	0	0
GntR	0	0 out of 6	0	0	0	0
H-NS	0	0 out of 6	0	0	0	0
IHF	15	39 out of 51	18	10	8	0
IclR	0	0 out of 2	0	0	0	0
IdnR	0	0 out of 3	0	0	0	0
IscR	1	4 out of 7	0	0	0	0
LacI	0	0 out of 1	0	0	0	0
LexA	6	11 out of 14	13	3	6	4
Lrp	5	13 out of 18	5	3	3	0
MalT	1	4 out of 5	0	0	0	0
MarA	2	7 out of 15	1	1	0	0
MelR	0	0 out of 2	0	0	0	0
MetJ	2	7 out of 8	0	0	0	0
MetR	0	0 out of 4	0	0	0	0
Mlc	5	5 out of 5	11	3	9	0
ModE	0	0 out of 6	0	0	0	0
Nac	0	0 out of 5	0	0	0	0
NagC	5	5 out of 6	15	0	3	12
NanR	0	0 out of 2	0	0	0	0

NarL	7	19 out of 20	0	0	0	0
NarP	1	4 out of 7	0	0	0	0
NorR	0	0 out of 1	0	0	0	0
NsrR	1	3 out of 4	1	1	0	0
NtrC	5	7 out of 7	8	3	5	0
OmpR	1	2 out of 8	2	2	0	0
OxyR	1	2 out of 8	2	2	0	0
PhoB	1	1 out of 10	4	0	4	0
PhoP	3	9 out of 19	1	1	0	0
PspF	0	0 out of 2	0	0	0	0
PurR	6	14 out of 15	11	8	3	0
RcsB	1	2 out of 7	2	2	0	0
RhaS	0	0 out of 2	0	0	0	0
Rob	0	0 out of 5	0	0	0	0
SoxS	2	8 out of 15	0	0	0	0
TorR	0	0 out of 3	0	0	0	0
TrpR	0	0 out of 5	0	0	0	0
TyrR	2	4 out of 8	3	3	0	0
UlaR	0	0 out of 2	0	0	0	0
UxuR	0	0 out of 3	0	0	0	0
XylR	0	0 out of 2	0	0	0	0
			278	157	118	20

Table S2: The complete list of novel interactions.

Regulator	Regulated bnumber	Regulated gene	Module
ArcA	b0007	yaaJ	128
ArcA	b0155	clcA	130
ArcA	b0280	yagN	128
ArcA	b0318	yahD	131
ArcA	b0598	cstA	90
ArcA	b0854	potF	130 91
ArcA	b1132	hflD	129
ArcA	b1388	paaA	18
ArcA	b1853	yebK	90
ArcA	b2070	yegI	92
ArcA	b2092	gatC	90
ArcA	b2093	gatB	90
ArcA	b2096	gatY	90
ArcA	b2193	narP	93
ArcA	b2380	ypdA	130
ArcA	b2518	ndk	94
ArcA	b2844	yqeF	18 90
ArcA	b3025	qseB	131
ArcA	b3128	garD	131
ArcA	b3237	argR	129
ArcA	b3739	atpI	91
ArcA	b4328	iadA	92
ArcA	b4376	osmY	28
ArgR	b0295	ykgL	54
ArgR	b0674	asnB	54
ArgR	b0860	artJ	10
ArgR	b2669	stpA	54
ArgR	b2818	argA	10
CRP	b0162	cdaR	115
CRP	b0268	yagE	114 137
CRP	b0459	maa	145
CRP	b0486	ybaT	116
CRP	b0592	fepB	71

CRP	b0822	ybiV	140
CRP	b0880	cspD	114 136
CRP	b0898	ycaD	140
CRP	b0953	rmf	116 135
CRP	b1002	agp	114 115 136
CRP	b1138	ymfE	149
CRP	b1139	lit	139
CRP	b1188	ycgB	145
CRP	b1241	adhE	15
CRP	b1255	yciC	148
CRP	b1397	paaJ	115
CRP	b1440	ydcS	114 115
CRP	b1492	gadC	116
CRP	b1498	ydeN	14
CRP	b1513	lsrA	115
CRP	b1685	ydiH	143
CRP	b1704	aroH	141
CRP	b1778	yeaA	142
CRP	b1814	sdaA	138
CRP	b1976	yeeI	114 136
CRP	b2097	fbaB	116
CRP	b2146	yeiT	136
CRP	b2155	cirA	71
CRP	b2536	hcaT	114 115
CRP	b2597	yfiA	114 116
CRP	b2702	srlA	136
CRP	b2715	ascF	117
CRP	b2775	yqcE	117
CRP	b2800	fucA	114 136
CRP	b2844	yqeF	114 115 18
CRP	b2869	ygeV	114 136 138
CRP	b2870	ygeW	117
CRP	b3072	aer	30
CRP	b3073	ygjG	56
CRP	b3093	exuT	137
CRP	b3238	yhcN	148
CRP	b3239	yhcO	138
CRP	b3253	yhdH	114
CRP	b3403	pck	114 135 136
CRP	b3453	ugpB	114 115
CRP	b3510	hdeA	116
CRP	b3683	glvC	117
CRP	b3830	ysgA	140
CRP	b3831	udp	12
CRP	b3882	yihU	114
CRP	b3909	kdgT	149
CRP	b3947	ptsA	139
CRP	b4142	groS	15
CRP	b4189	yjfO	114 115
CRP	b4208	cycA	147
CRP	b4216	ytfJ	115 136
CRP	b4227	ytfQ	114 115 135 136
CRP	b4238	nrdD	114
CRP	b4299	yjhI	136
CRP	b4354	yjiY	138
CRP	b4355	tsr	30
CRP	b4380	yjjI	6
CRP	b4383	deoB	6
CpxR	b0970	yccA	27

CpxR	b3913		27
CpxR	b3914		27
CpxR	b4143	groL	101
CpxR	b4219	msrA	101
CueR	b0209	yafD	31
CysB	b0058	rluA	38
CysB	b0427	yajR	38
CysB	b1287	yciW	23
CysB	b1627	rsxA	38
CysB	b2375	yfdX	38
CysB	b4311	yjhA	23
CytR	b3831	udp	12
DeoR	b0683	fur	6
DeoR	b4380	yjjI	6
DeoR	b4383	deoB	6
FNR	b0643	ybeL	106
FNR	b0650	hscC	83
FNR	b1002	agp	106
FNR	b1003	yccJ	106
FNR	b1004	wrbA	106
FNR	b1256	ompW	45
FNR	b1358	ydaT	84
FNR	b1376	uspF	106
FNR	b1541	ydfZ	45
FNR	b1593	ynfK	45
FNR	b1674	ydhY	80
FNR	b1750	ydjX	84
FNR	b1901	araF	20
FNR	b1902	yecI	106 87
FNR	b2111	yehD	87
FNR	b2504	yfgG	80
FNR	b2580	ung	85
FNR	b2729	hypD	45
FNR	b3518	yhjA	87
FNR	b3519	treF	86
FNR	b4278	insG	85
FNR	b4279	yjhB	84
FNR	b4326	yjiD	84
FNR	b4380	yjjI	45
FNR	b4403	yjtD	84
Fis	b0469	apt	35
Fis	b0779	uvrB	112
Fis	b0835	yliG	35
Fis	b1185	dsbB	69
Fis	b1344	ydaO	35
Fis	b1779	gapA	15
Fis	b1876	argS	35
Fis	b2296	ackA	113 68
Fis	b2398	yfeC	113
Fis	b2549	yphG	67
Fis	b2550	yphH	67
Fis	b2821	ptr	112
Fis	b2927	epd	111
Fis	b3000	yghY	67
Fis	b3072	aer	30
Fis	b3260	dusB	35
Fis	b3813	uvrD	69
Fis	b4108	phnA	68
Fis	b4142	groS	15

Fis	b4355	tsr	30
FlhDC	b1078	flgG	3
FlhDC	b1926	fliT	3
FlhDC	b1943	fliK	3
FlhDC	b1945	fliM	3
FlhDC	b2625	yfjI	132
FruR	b2096	gatY	88
FruR	b2169	fruB	43
FruR	b2340	sixA	88
FruR	b2597	yfiA	88
FruR	b3299	rpmJ	89
FruR	b3357	crp	42 89
FruR	b3956	ppc	89
Fur	b0150	fhuA	21 5
Fur	b0468	ybaN	21 70
Fur	b1018	ycdO	21
Fur	b1374	pinR	122
Fur	b1451	yncD	21
Fur	b1452	yncE	21 5 70
Fur	b1705	ydiE	5
Fur	b2211	yojI	21
Fur	b2392	mntH	121 70
Fur	b2393	nupC	122
Fur	b2675	nrdE	5 70
Fur	b3008	metC	121
Fur	b3070	yqjH	21 5
Fur	b3337	bfd	5 70
Fur	b3440	yhhX	121
GadE	b3490	yhiL	40
GadE	b4326	yjiD	40
IHF	b0098	secA	126
IHF	b0280	yagN	123
IHF	b0432	cyoA	74
IHF	b0565	ompT	75
IHF	b0754	aroG	75
IHF	b0992	yccM	79
IHF	b1338	abgA	127
IHF	b1344	ydaO	124
IHF	b1908	yecA	79
IHF	b2508	guaB	74
IHF	b3320	rplC	75
IHF	b3554	yiaF	124
IHF	b3650	spoT	125
IHF	b3734	atpA	75
IHF	b3736	atpF	75
IHF	b3784	rfe	74
IHF	b4138	dcuA	126
IHF	b4332	yjiJ	77
LexA	b0019	nhaA	103
LexA	b0226	dinJ	103
LexA	b0227	yafL	63
LexA	b0231	dinB	62
LexA	b0685	ybfE	102
LexA	b1061	dinI	61
LexA	b1848	yebG	33
LexA	b2009	sbmC	103
LexA	b3391	hofQ	63
LexA	b3501	arsR	63
LexA	b3645	dinD	62

LexA	b3673	emrD	63
LexA	b3832	rmuC	62
Lrp	b0891	lolA	64
Lrp	b0922	mukF	65
Lrp	b0966	yccV	66
Lrp	b1660	ydhC	66
Lrp	b3617	kbl	109 66
MarA	b4107	phnB	36
Mlc	b0426	yajQ	108
Mlc	b0678	nagB	13
Mlc	b0796	ybiH	108
Mlc	b1387	maoC	100
Mlc	b1498	ydeN	14
Mlc	b1617	uidA	13
Mlc	b3147	yraM	108
Mlc	b3417	malP	100 13
Mlc	b3862	yihG	107
Mlc	b3884	yihW	107
Mlc	b3900	frvA	107
NagC	b0191	yaeJ	60
NagC	b0394	mak	58
NagC	b0681	ybfM	57
NagC	b1266	yciV	60
NagC	b1654	ydhD	60
NagC	b2112	yehE	58
NagC	b2443	yffL	59
NagC	b2774	ygcW	57
NagC	b2846	yqeH	59
NagC	b2987	pitB	60
NagC	b3427	yzgL	57
NagC	b3688	yidQ	58
NagC	b3717	cbrC	59
NagC	b3746	yieN	58
NagC	b4311	yjhA	59
NsrR	b1426	ydcH	51
NtrC	b0470	dnaX	133
NtrC	b1012	ycdM	134
NtrC	b1823	cspC	72
NtrC	b1824	yobF	72
NtrC	b2309	hisJ	133
NtrC	b3871	bipA	133 73
NtrC	b3973	birA	73
NtrC	b4146	yjeK	73
OmpR	b0598	cstA	47
OmpR	b0811	glnH	47
OxyR	b1688	ydiK	46
OxyR	b4387	ytjB	46
PhoB	b2720	hycF	44
PhoB	b2884		44
PhoB	b3211	yhcC	44
PhoB	b4464	ygfQ	44
PhoP	b1505	ydeT	53
PurR	b0336	codB	24
PurR	b0411	tsx	97
PurR	b1039	csgE	96
PurR	b1849	purT	97
PurR	b2313	cvpA	24 8
PurR	b2498	upp	95
PurR	b2682	ygaZ	24

PurR	b2888	ygfU	95
PurR	b3654	yicE	39
PurR	b3714	yieG	97
PurR	b3823	rhtC	39
RcsB	b0419	yajO	37
RcsB	b4377	yjjU	37
TyrR	b0406	tgt	41
TyrR	b1842	holE	98
TyrR	b1894	insA-5	98
