# Supplementary material for: DISTILLER: a data integration framework to reveal condition dependency of complex regulons in Escherichia coli
Source: Genome Biol. 2009 Mar 6;10(3):R27. doi: 10.1186/gb-2009-10-3-r27 (PMC2690998; doi:10.1186/gb-2009-10-3-r27)
Supplement: Additional data file 3 — A more detailed description of Figure 1. [file gb-2009-10-3-r27-S3.pdf]

ADDITIONAL DATA FILE 3: Conditional dependency of the regulatory network

Arrays were grouped into conditional categories depending on the major cue that was changed in the experiments of the compendium (this categorization is shown in Table S1). For instance, the category *aerobic-anaerobic* groups all arrays in which the effect of changing the oxygen level on gene expression was measured.

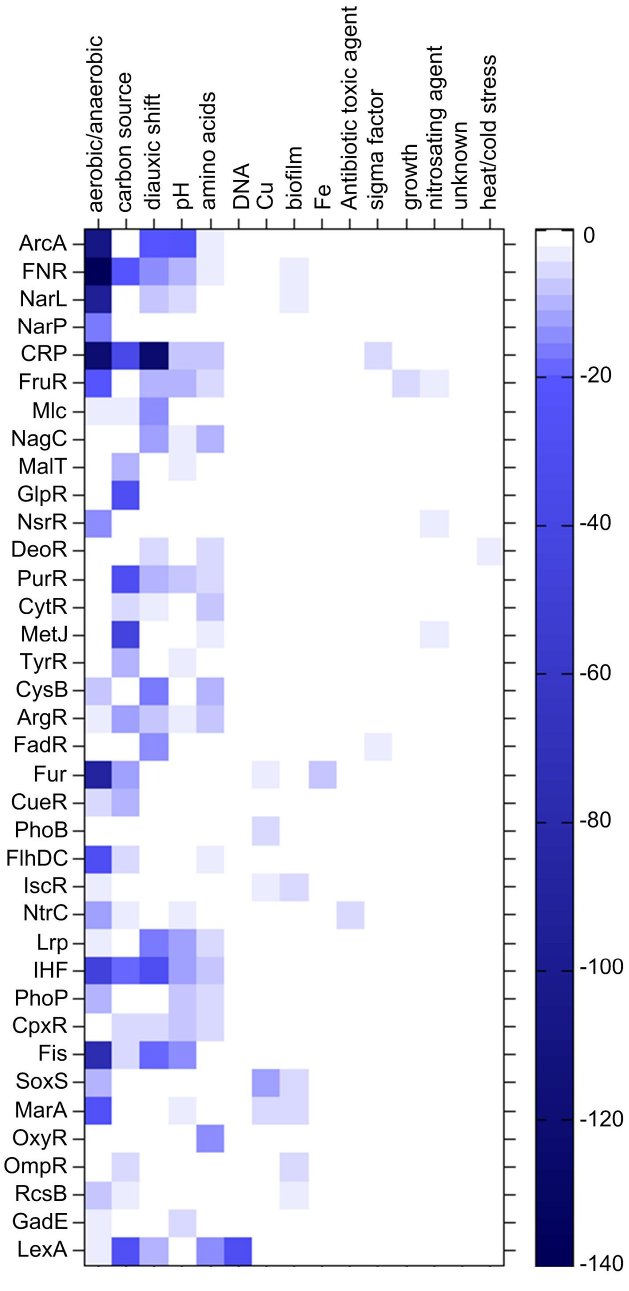

Figure S1: Columns: Conditional categories. Rows: regulators for which modules were detected by DISTILLER. Each entry indicates to what extent the conditions of the modules of a particular regulator are enriched (log p-value) for a specific category.

Figure S1 shows to what extent the conditions of the modules of a particular regulator are enriched for a specific category. Enrichment of a conditional category thus implies that the target genes of a particular regulator are mainly co-expressed in conditions belonging to the enriched category and this indirectly gives information on the conditions where a particular regulator is active. As the global metabolism has to be tuned towards the simultaneous interplay of several triggers such as oxygen, carbon source and amino acid availability, cross talk between conditional categories is to be expected: the more categories a regulator is involved in, the more global its role is in coordinating the overall cellular response. In general, most regulators are active in conditions that are in agreement with their annotation. A more detailed description follows next.

Oxygen responsive regulators

The well-known regulators responsive to oxygen, redox levels and alternative electron acceptors (FNR, ArcA, NarL and NarP) [49] are among the most overrepresented in the conditional category *aerobic-anaerobic*. To some extent their activity is also visible during *diauxic shift*. This is to be expected as changes in the expression levels of genes of the respiratory chain should be tuned towards the reducing power generated during carbon catabolism switches.

Regulators of the carbon metabolism

The regulators of carbon metabolism (CRP, MalT, Mlc, NagC, FruR, GlpR) are active in different conditional categories. The global regulator CRP has the most pronounced pleiotropic behavior and is active in the categories *aerobic-anaerobic*, *diauxic shift*, *carbon source* and *amino acids*. FruR and Mlc (and its paralog NagC [28]), regulators of respectively glyconeogenesis and glycolysis, seem mainly active in *diauxic shift* conditions. FruR activity also depends on changes in the oxygen concentration and to a lower extent on amino acid availability, indicating that FruR together with CRP tunes the level of carbon source utilization towards oxygen and amino acid availability. Although they are paralogs, Mlc is mainly active during *diauxic shift*, while NagC modules are also linked to *amino acids* conditions. This is to be expected as Mlc is mainly sensitive to glucose levels while NagC repressed genes are derepressed in the presence of amino sugars (N-acetylglucosamine (GlcNAc) [28]. Modules of the more specific regulators MalT and GlpR, involved respectively in the regulation of maltose and glycerol catabolism, are mainly expressed in the conditional category *carbon source*, upon addition of their respective carbon sources to the medium.

Regulators of nucleoside and amino acid biosynthesis

The conditions of modules involved in synthesis of novel molecular building blocks (nucleotides (DeoR, PurR, CytR), amino acids (CysB, Lrp, ArgR) and fatty acids (FadR)) are all enriched for the *diauxic shift* category. This is not surprising as most diauxic experiments assess growth recovery after starvation: the growth renewal requires the synthesis of novel building blocks. The conditional categories where regulators of purine and pyrimidine metabolism (DeoR, PurR, and CytR) are active suggest partial complementary: DeoR is mainly active during *diauxic shift* and *amino acid* starvation, CytR during *amino acid* starvation and PurR during *diauxic shift* and alterations of the *carbon source*. The condition sets of the modules involved in the biosynthesis of methionine, tyrosine and arginine (MetJ, TyrR and ArgR modules) are overrepresented in the *carbon source* category. Modules regulated by the nitrogen sensor NtrBC contain mainly conditions related to *diauxic shift*, and *amino acid* and *carbon source* alterations, confirming the role of the two-component system NtrBC in tuning the nitrogen availability to the carbon source and energy availability [50].

Regulators sensitive towards Iron/Cu

The role of Fur is very pronounced during *iron* related conditions but also during conditions which alter the oxygen level (*aerobic-anaerobic*). Indeed, Fe^{2+} is known to be an essential component of the prosthetic groups in enzymes involved in the electron transport chain and in protection against oxidative stress. The synthesis of these proteins not only depends on the oxygen concentration but also needs to be tuned towards the iron availability [51]. CueR, the regulator of the copper efflux system *copA,* is induced upon elevated copper levels [52], but does not seem active during comparable conditions in our compendium.

Nucleoid associated regulators

By acting both as a specific transcriptional regulator and nucleoid-associated protein, Lrp functions as a global regulator that is responsive to medium quality changes (starvation, osmolarity, …) and involved in the biosynthesis and transport of molecules (amino acids, rRNA) [53-55]. According to our results its modules are mainly enriched for *diauxic shift* and *pH* related stress conditions. Similarly, modules of the nucleoid associated proteins Fis and IHF are active in a whole range of (stress) related conditions (carbon source starvation, pH dependent and O_2 related stress conditions) [27].

Stress responsive regulators

The other stress related regulators seem to act in more specific conditions. LexA, for instance, is apparently the only regulator with a clear activity during conditions that induce *DNA damage* [56,57]. Starvation conditions (*carbon source* and *amino acid* starvation) also induce the LexA dependent pathways.

The modules of both the PhoPQ two-component system, a pleiotropic regulator known to be induced by low pH [58,59], and CpxR, activated by envelope stress which results in protein misfolding (such as alkaline conditions) [60], are mainly enriched in *pH* induced stress conditions.

The SoxS (regulator of the superoxide response regulon) and MarA regulators (multiple antibiotic resistance) seem to act in concert in the detoxification of externally added copper, during growth on biofilm and upon changes in the cellular oxygen level. The OxyR regulator, mainly active during oxygen changes [61], may activate the Fur regulon and protect the cell by scavenging iron (expression of *dps*, a stationary phase nucleoid protein that sequesters iron and protects DNA from damage).

Biofilm related conditions

OmpR, a known major regulator of membrane remodeling during growth on biofilms [29], and RscB, also a known regulator of growth during biofilm formation [30], are indeed overrepresented in *biofilm* conditions but may also play a role in alterations of the *carbon source* (both OmpR and RscB) or in oxygen changes i.e. *aerobic-anaerobic* (only RscB). GadE, known to be involved in the resistance to low pH [62], is mainly activated upon *diauxic shift* but also upon changes in the *pH* of the medium. Although CpxR has recently been described as a biofilm related regulator [31], it does not seem to be overrepresented in the biofilm related conditions present in our compendium.

Table S1: Information on the arrays that are present in the microarray compendium. ArrayID: a unique identifier for each array. **Array**: the array name. **Exp. ID**: a unique identifier assigned to all experiments. **Experiment:** the experiment name. **Funct. category ID:** a unique identifier indicating the conditional category. **Functional category:** arrays were grouped into functional categories depending on the major cue that was changed in the experiments of the compendium.

Array ID	Array	Exp. ID	Experiment	Funct. category ID	Functional category
86	EC_GLU_WILD_A	1	Palsson_2005_Genome_Res	1	carbon_source
87	EC_GLU_WILD_B	1	Palsson_2005_Genome_Res	1	carbon_source
88	EC_GLU_WILD_C	1	Palsson_2005_Genome_Res	1	carbon_source
1	EC_GLY_GLY1_20_A	1	Palsson_2005_Genome_Res	1	carbon_source
2	EC_GLY_GLY1_20_B	1	Palsson_2005_Genome_Res	1	carbon_source
3	EC_GLY_GLY1_20_C	1	Palsson_2005_Genome_Res	1	carbon_source
4	EC_GLY_GLY1_44_A	1	Palsson_2005_Genome_Res	1	carbon_source
5	EC_GLY_GLY1_44_B	1	Palsson_2005_Genome_Res	1	carbon_source
6	EC_GLY_GLY1_44_C	1	Palsson_2005_Genome_Res	1	carbon_source
7	EC_GLY_GLY2_20_A	1	Palsson_2005_Genome_Res	1	carbon_source
8	EC_GLY_GLY2_20_B	1	Palsson_2005_Genome_Res	1	carbon_source
9	EC_GLY_GLY2_20_C	1	Palsson_2005_Genome_Res	1	carbon_source
10	EC_GLY_GLY2_44_A	1	Palsson_2005_Genome_Res	1	carbon_source
11	EC_GLY_GLY2_44_B	1	Palsson_2005_Genome_Res	1	carbon_source
12	EC_GLY_GLY2_44_C	1	Palsson_2005_Genome_Res	1	carbon_source
13	EC_GLY_GLYA_20_A	1	Palsson_2005_Genome_Res	1	carbon_source
14	EC_GLY_GLYA_20_B	1	Palsson_2005_Genome_Res	1	carbon_source
15	EC_GLY_GLYA_20_C	1	Palsson_2005_Genome_Res	1	carbon_source
16	EC_GLY_GLYA_44_A	1	Palsson_2005_Genome_Res	1	carbon_source
17	EC_GLY_GLYA_44_B	1	Palsson_2005_Genome_Res	1	carbon_source
18	EC_GLY_GLYA_44_C	1	Palsson_2005_Genome_Res	1	carbon_source
19	EC_GLY_GLYB_20_A	1	Palsson_2005_Genome_Res	1	carbon_source
20	EC_GLY_GLYB_20_B	1	Palsson_2005_Genome_Res	1	carbon_source
21	EC_GLY_GLYB_20_C	1	Palsson_2005_Genome_Res	1	carbon_source
22	EC_GLY_GLYB_44_A	1	Palsson_2005_Genome_Res	1	carbon_source
23	EC_GLY_GLYB_44_B	1	Palsson_2005_Genome_Res	1	carbon_source
24	EC_GLY_GLYB_44_C	1	Palsson_2005_Genome_Res	1	carbon_source
25	EC_GLY_GLYC_20_A	1	Palsson_2005_Genome_Res	1	carbon_source
26	EC_GLY_GLYC_20_B	1	Palsson_2005_Genome_Res	1	carbon_source
27	EC_GLY_GLYC_20_C	1	Palsson_2005_Genome_Res	1	carbon_source
28	EC_GLY_GLYC_44_A	1	Palsson_2005_Genome_Res	1	carbon_source
29	EC_GLY_GLYC_44_B	1	Palsson_2005_Genome_Res	1	carbon_source
30	EC_GLY_GLYC_44_C	1	Palsson_2005_Genome_Res	1	carbon_source
31	EC_GLY_GLYD_20_A	1	Palsson_2005_Genome_Res	1	carbon_source
32	EC_GLY_GLYD_20_B	1	Palsson_2005_Genome_Res	1	carbon_source
33	EC_GLY_GLYD_20_C	1	Palsson_2005_Genome_Res	1	carbon_source
34	EC_GLY_GLYD_44_A	1	Palsson_2005_Genome_Res	1	carbon_source
35	EC_GLY_GLYD_44_B	1	Palsson_2005_Genome_Res	1	carbon_source
36	EC_GLY_GLYD_44_C	1	Palsson_2005_Genome_Res	1	carbon_source
37	EC_GLY_GLYE_20_A	1	Palsson_2005_Genome_Res	1	carbon_source
38	EC_GLY_GLYE_20_B	1	Palsson_2005_Genome_Res	1	carbon_source
39	EC_GLY_GLYE_20_C	1	Palsson_2005_Genome_Res	1	carbon_source
40	EC_GLY_GLYE_44_A	1	Palsson_2005_Genome_Res	1	carbon_source
41	EC_GLY_GLYE_44_B	1	Palsson_2005_Genome_Res	1	carbon_source
42	EC_GLY_GLYE_44_C	1	Palsson_2005_Genome_Res	1	carbon_source
89	EC_GLY_WILD_A	1	Palsson_2005_Genome_Res	1	carbon_source
90	EC_GLY_WILD_B	1	Palsson_2005_Genome_Res	1	carbon_source
91	EC_GLY_WILD_C	1	Palsson_2005_Genome_Res	1	carbon_source
92	EC_GLY_WILD_D	1	Palsson_2005_Genome_Res	1	carbon_source
93	EC_GLY_WILD_E	1	Palsson_2005_Genome_Res	1	carbon_source
43	EC_LAC_L2_20_A	1	Palsson_2005_Genome_Res	1	carbon_source
44	EC_LAC_L2_20_B	1	Palsson_2005_Genome_Res	1	carbon_source
45	EC_LAC_L2_20_C	1	Palsson_2005_Genome_Res	1	carbon_source
46	EC_LAC_L2_60_A	1	Palsson_2005_Genome_Res	1	carbon_source
47	EC_LAC_L2_60_B	1	Palsson_2005_Genome_Res	1	carbon_source
48	EC_LAC_L2_60_C	1	Palsson_2005_Genome_Res	1	carbon_source
49	EC_LAC_L3_20_A	1	Palsson_2005_Genome_Res	1	carbon_source

50	EC_LAC_L3_20_B	1	Palsson_2005_Genome_Res	1	carbon_source
51	EC_LAC_L3_20_C	1	Palsson_2005_Genome_Res	1	carbon_source
52	EC_LAC_L3_60_A	1	Palsson_2005_Genome_Res	1	carbon_source
53	EC_LAC_L3_60_B	1	Palsson_2005_Genome_Res	1	carbon_source
54	EC_LAC_L3_60_C	1	Palsson_2005_Genome_Res	1	carbon_source
55	EC_LAC_LA_20_A	1	Palsson_2005_Genome_Res	1	carbon_source
56	EC_LAC_LA_20_B	1	Palsson_2005_Genome_Res	1	carbon_source
57	EC_LAC_LA_20_C	1	Palsson_2005_Genome_Res	1	carbon_source
58	EC_LAC_LA_60_A	1	Palsson_2005_Genome_Res	1	carbon_source
59	EC_LAC_LA_60_B	1	Palsson_2005_Genome_Res	1	carbon_source
60	EC_LAC_LA_60_C	1	Palsson_2005_Genome_Res	1	carbon_source
61	EC_LAC_LA_60_D	1	Palsson_2005_Genome_Res	1	carbon_source
62	EC_LAC_LB_20_A	1	Palsson_2005_Genome_Res	1	carbon_source
63	EC_LAC_LB_20_B	1	Palsson_2005_Genome_Res	1	carbon_source
64	EC_LAC_LB_20_C	1	Palsson_2005_Genome_Res	1	carbon_source
65	EC_LAC_LB_60_A	1	Palsson_2005_Genome_Res	1	carbon_source
66	EC_LAC_LB_60_B	1	Palsson_2005_Genome_Res	1	carbon_source
67	EC_LAC_LB_60_C	1	Palsson_2005_Genome_Res	1	carbon_source
68	EC_LAC_LC_20_A	1	Palsson_2005_Genome_Res	1	carbon_source
69	EC_LAC_LC_20_B	1	Palsson_2005_Genome_Res	1	carbon_source
70	EC_LAC_LC_20_C	1	Palsson_2005_Genome_Res	1	carbon_source
71	EC_LAC_LC_60_A	1	Palsson_2005_Genome_Res	1	carbon_source
72	EC_LAC_LC_60_B	1	Palsson_2005_Genome_Res	1	carbon_source
73	EC_LAC_LC_60_C	1	Palsson_2005_Genome_Res	1	carbon_source
74	EC_LAC_LD_20_A	1	Palsson_2005_Genome_Res	1	carbon_source
75	EC_LAC_LD_20_B	1	Palsson_2005_Genome_Res	1	carbon_source
76	EC_LAC_LD_20_C	1	Palsson_2005_Genome_Res	1	carbon_source
77	EC_LAC_LD_60_A	1	Palsson_2005_Genome_Res	1	carbon_source
78	EC_LAC_LD_60_B	1	Palsson_2005_Genome_Res	1	carbon_source
79	EC_LAC_LD_60_C	1	Palsson_2005_Genome_Res	1	carbon_source
80	EC_LAC_LE_20_A	1	Palsson_2005_Genome_Res	1	carbon_source
81	EC_LAC_LE_20_B	1	Palsson_2005_Genome_Res	1	carbon_source
82	EC_LAC_LE_20_C	1	Palsson_2005_Genome_Res	1	carbon_source
83	EC_LAC_LE_60_A	1	Palsson_2005_Genome_Res	1	carbon_source
84	EC_LAC_LE_60_B	1	Palsson_2005_Genome_Res	1	carbon_source
85	EC_LAC_LE_60_C	1	Palsson_2005_Genome_Res	1	carbon_source
94	EC_LAC_WILD_A	1	Palsson_2005_Genome_Res	1	carbon_source
95	EC_LAC_WILD_B	1	Palsson_2005_Genome_Res	1	carbon_source
96	EC_LAC_WILD_C	1	Palsson_2005_Genome_Res	1	carbon_source
97	EC_LAC_WILD_D	1	Palsson_2005_Genome_Res	1	carbon_source
98	EC_LAC_WILD_E	1	Palsson_2005_Genome_Res	1	carbon_source
99	EC_LAC_WILD_F	1	Palsson_2005_Genome_Res	1	carbon_source
256	Clean_ANA12	10	Chang_2002_Mol_Biol	2	aerobic__anaerobic
257	Clean_ANA13	10	Chang_2002_Mol_Biol	2	aerobic__anaerobic
258	Clean_ANA15	10	Chang_2002_Mol_Biol	2	aerobic__anaerobic
259	Clean_ANA17	10	Chang_2002_Mol_Biol	2	aerobic__anaerobic
251	Clean_ANA2	10	Chang_2002_Mol_Biol	2	aerobic__anaerobic
260	Clean_ANA20	10	Chang_2002_Mol_Biol	2	aerobic__anaerobic
252	Clean_ANA3	10	Chang_2002_Mol_Biol	2	aerobic__anaerobic
253	Clean_ANA6	10	Chang_2002_Mol_Biol	2	aerobic__anaerobic
254	Clean_ANA7	10	Chang_2002_Mol_Biol	2	aerobic__anaerobic
255	Clean_ANA9	10	Chang_2002_Mol_Biol	2	aerobic__anaerobic
296	Clean_DIAUX10	10	Chang_2002_Mol_Biol	7	diauxic_shift
297	Clean_DIAUX11	10	Chang_2002_Mol_Biol	7	diauxic_shift
298	Clean_DIAUX12	10	Chang_2002_Mol_Biol	7	diauxic_shift
299	Clean_DIAUX13	10	Chang_2002_Mol_Biol	7	diauxic_shift
300	Clean_DIAUX14	10	Chang_2002_Mol_Biol	7	diauxic_shift
301	Clean_DIAUX15	10	Chang_2002_Mol_Biol	7	diauxic_shift
302	Clean_DIAUX16	10	Chang_2002_Mol_Biol	7	diauxic_shift
303	Clean_DIAUX17	10	Chang_2002_Mol_Biol	7	diauxic_shift
288	Clean_DIAUX2	10	Chang_2002_Mol_Biol	7	diauxic_shift
289	Clean_DIAUX3	10	Chang_2002_Mol_Biol	7	diauxic_shift
290	Clean_DIAUX4	10	Chang_2002_Mol_Biol	7	diauxic_shift
291	Clean_DIAUX5	10	Chang_2002_Mol_Biol	7	diauxic_shift
292	Clean_DIAUX6	10	Chang_2002_Mol_Biol	7	diauxic_shift
293	Clean_DIAUX7	10	Chang_2002_Mol_Biol	7	diauxic_shift
294	Clean_DIAUX8	10	Chang_2002_Mol_Biol	7	diauxic_shift
295	Clean_DIAUX9	10	Chang_2002_Mol_Biol	7	diauxic_shift

277	Clean_H2O21	10	Chang_2002_Mol_Biol	7	diauxic_shift
286	Clean_H2O210	10	Chang_2002_Mol_Biol	7	diauxic_shift
287	Clean_H2O211	10	Chang_2002_Mol_Biol	7	diauxic_shift
278	Clean_H2O22	10	Chang_2002_Mol_Biol	7	diauxic_shift
279	Clean_H2O23	10	Chang_2002_Mol_Biol	7	diauxic_shift
280	Clean_H2O24	10	Chang_2002_Mol_Biol	7	diauxic_shift
281	Clean_H2O25	10	Chang_2002_Mol_Biol	7	diauxic_shift
282	Clean_H2O26	10	Chang_2002_Mol_Biol	7	diauxic_shift
283	Clean_H2O27	10	Chang_2002_Mol_Biol	7	diauxic_shift
284	Clean_H2O28	10	Chang_2002_Mol_Biol	7	diauxic_shift
285	Clean_H2O29	10	Chang_2002_Mol_Biol	7	diauxic_shift
241	Clean_TGC1	10	Chang_2002_Mol_Biol	2	aerobic__anaerobic
246	Clean_TGC10	10	Chang_2002_Mol_Biol	2	aerobic__anaerobic
247	Clean_TGC12	10	Chang_2002_Mol_Biol	2	aerobic__anaerobic
248	Clean_TGC14	10	Chang_2002_Mol_Biol	2	aerobic__anaerobic
249	Clean_TGC16	10	Chang_2002_Mol_Biol	2	aerobic__anaerobic
250	Clean_TGC18	10	Chang_2002_Mol_Biol	2	aerobic__anaerobic
242	Clean_TGC2	10	Chang_2002_Mol_Biol	2	aerobic__anaerobic
243	Clean_TGC5	10	Chang_2002_Mol_Biol	2	aerobic__anaerobic
244	Clean_TGC6	10	Chang_2002_Mol_Biol	2	aerobic__anaerobic
245	Clean_TGC8	10	Chang_2002_Mol_Biol	2	aerobic__anaerobic
261	GADXW_exp1	10	Chang_2002_Mol_Biol	6	pH
270	GADXW_exp10	10	Chang_2002_Mol_Biol	6	pH
271	GADXW_exp11	10	Chang_2002_Mol_Biol	6	pH
272	GADXW_exp12	10	Chang_2002_Mol_Biol	6	pH
273	GADXW_exp13	10	Chang_2002_Mol_Biol	6	pH
274	GADXW_exp14	10	Chang_2002_Mol_Biol	6	pH
275	GADXW_exp15	10	Chang_2002_Mol_Biol	6	pH
276	GADXW_exp16	10	Chang_2002_Mol_Biol	6	pH
262	GADXW_exp2	10	Chang_2002_Mol_Biol	6	pH
263	GADXW_exp3	10	Chang_2002_Mol_Biol	6	pH
264	GADXW_exp4	10	Chang_2002_Mol_Biol	6	pH
265	GADXW_exp5	10	Chang_2002_Mol_Biol	6	pH
266	GADXW_exp6	10	Chang_2002_Mol_Biol	6	pH
267	GADXW_exp7	10	Chang_2002_Mol_Biol	6	pH
268	GADXW_exp8	10	Chang_2002_Mol_Biol	6	pH
269	GADXW_exp9	10	Chang_2002_Mol_Biol	6	pH
304	GSM62194	11	GSE2827	10	antibiotic__toxic_agent
305	GSM62195	11	GSE2827	10	antibiotic__toxic_agent
306	GSM62196	11	GSE2827	10	antibiotic__toxic_agent
307	GSM62197	11	GSE2827	10	antibiotic__toxic_agent
308	GSM62198	11	GSE2827	10	antibiotic__toxic_agent
309	GSM62199	11	GSE2827	10	antibiotic__toxic_agent
310	GSM7886	12	GSE533	9	heat_cold_stress
311	GSM8110	12	GSE533	9	heat_cold_stress
312	GSM8111	12	GSE533	9	heat_cold_stress
313	GSM8112	12	GSE533	9	heat_cold_stress
314	GSM8113	12	GSE533	9	heat_cold_stress
315	GSM8114	12	GSE533	9	heat_cold_stress
316	GSM8115	12	GSE533	9	heat_cold_stress
317	GSM8116	12	GSE533	9	heat_cold_stress
318	GSM8117	12	GSE533	9	heat_cold_stress
319	GSM8118	12	GSE533	9	heat_cold_stress
320	GSM8119	12	GSE533	9	heat_cold_stress
321	GSM8120	12	GSE533	9	heat_cold_stress
322	E-MEXP-222-raw-data-386284924.txt	13	E-MEXP-222	10	antibiotic__toxic_agent
323	E-MEXP-222-raw-data-386284973.txt	13	E-MEXP-222	10	antibiotic__toxic_agent
324	E-MEXP-222-raw-data-386285007.txt	13	E-MEXP-222	10	antibiotic__toxic_agent
325	E-MEXP-222-raw-data-386285038.txt	13	E-MEXP-222	10	antibiotic__toxic_agent
326	E-MEXP-222-raw-data-386285069.txt	13	E-MEXP-222	10	antibiotic__toxic_agent
327	E-MEXP-222-raw-data-386285101.txt	13	E-MEXP-222	10	antibiotic__toxic_agent
328	E-MEXP-244-raw-data-	14	E-MEXP-244	10	antibiotic__toxic_agent

	396455050.txt				
329	E-MEXP-244-raw-data-396455066.txt	14	E-MEXP-244	10	antibiotic__toxic_agent
330	E-MEXP-244-raw-data-396455082.txt	14	E-MEXP-244	10	antibiotic__toxic_agent
331	E-MEXP-244-raw-data-396455098.txt	14	E-MEXP-244	10	antibiotic__toxic_agent
332	E-MEXP-245-raw-data-396455333.txt	15	E-MEXP-245	10	antibiotic__toxic_agent
333	E-MEXP-245-raw-data-396455349.txt	15	E-MEXP-245	10	antibiotic__toxic_agent
334	E-MEXP-245-raw-data-396455365.txt	15	E-MEXP-245	10	antibiotic__toxic_agent
335	E-MEXP-245-raw-data-396455381.txt	15	E-MEXP-245	10	antibiotic__toxic_agent
336	E-MEXP-245-raw-data-396455397.txt	15	E-MEXP-245	10	antibiotic__toxic_agent
337	E-MEXP-245-raw-data-396455413.txt	15	E-MEXP-245	10	antibiotic__toxic_agent
338	E-MEXP-267-raw-data-495080191.txt	18	E-MEXP-267	3	DNA
339	E-MEXP-267-raw-data-495080207.txt	18	E-MEXP-267	3	DNA
340	E-MEXP-267-raw-data-495080223.txt	18	E-MEXP-267	3	DNA
341	E-MEXP-267-raw-data-495080239.txt	18	E-MEXP-267	3	DNA
342	E-MEXP-267-raw-data-495080255.txt	18	E-MEXP-267	3	DNA
343	E-MEXP-267-raw-data-495080271.txt	18	E-MEXP-267	3	DNA
344	E-MEXP-267-raw-data-495080287.txt	18	E-MEXP-267	3	DNA
345	E-MEXP-267-raw-data-495080303.txt	18	E-MEXP-267	3	DNA
346	E-MEXP-267-raw-data-495080319.txt	18	E-MEXP-267	3	DNA
347	E-MEXP-267-raw-data-495080335.txt	18	E-MEXP-267	3	DNA
348	E-MEXP-267-raw-data-495080351.txt	18	E-MEXP-267	3	DNA
349	E-MEXP-267-raw-data-495080367.txt	18	E-MEXP-267	3	DNA
350	ANAEROBIC_CH	19	Blattner_2005_Genome_Res	2	aerobic__anaerobic
351	HEATSHOCK_CH	19	Blattner_2005_Genome_Res	9	heat_cold_stress
352	STATIONARYPHASE_CH	19	Blattner_2005_Genome_Res	14	growth
102	crp	2	Gossett_2005_J_of_Bact	1	carbon_source
103	crp__G	2	Gossett_2005_J_of_Bact	1	carbon_source
110	crpcysB	2	Gossett_2005_J_of_Bact	1	carbon_source
111	crpcysB__G	2	Gossett_2005_J_of_Bact	1	carbon_source
106	crpfruR	2	Gossett_2005_J_of_Bact	1	carbon_source
107	crpfruR__G	2	Gossett_2005_J_of_Bact	1	carbon_source
114	crpfur	2	Gossett_2005_J_of_Bact	1	carbon_source
115	crpfur__G	2	Gossett_2005_J_of_Bact	1	carbon_source
108	cysB	2	Gossett_2005_J_of_Bact	1	carbon_source
109	cysB__G	2	Gossett_2005_J_of_Bact	1	carbon_source
104	fruR	2	Gossett_2005_J_of_Bact	1	carbon_source
105	fruR__G	2	Gossett_2005_J_of_Bact	1	carbon_source
112	fur	2	Gossett_2005_J_of_Bact	1	carbon_source
113	fur__G	2	Gossett_2005_J_of_Bact	1	carbon_source
100	wt	2	Gossett_2005_J_of_Bact	1	carbon_source
101	wt__G	2	Gossett_2005_J_of_Bact	1	carbon_source
353	GSM1367	20	GSE33	3	DNA
354	GSM1368	20	GSE33	3	DNA
355	GSM1369	20	GSE33	3	DNA
356	GSM1370	20	GSE33	3	DNA
357	GSM1371	20	GSE33	3	DNA
358	GSM1372	20	GSE33	3	DNA
359	GSM1373	20	GSE33	3	DNA

360	GSM1374	20	GSE33	3	DNA
361	GSM1375	20	GSE33	3	DNA
362	GSM1376	20	GSE33	3	DNA
363	GSM1377	20	GSE33	3	DNA
364	GSM1378	20	GSE33	3	DNA
365	GSM1379	20	GSE33	3	DNA
366	GSM1380	20	GSE33	3	DNA
367	GSM1381	20	GSE33	3	DNA
368	GSM1382	20	GSE33	3	DNA
369	GSM23459	21	GSE1421	1	carbon_source
370	GSM23465	21	GSE1421	1	carbon_source
371	GSM23467	21	GSE1421	1	carbon_source
372	GSM30815	22	GSE1780	11	Cu
373	GSM30816	22	GSE1780	11	Cu
374	GSM30817	22	GSE1780	11	Cu
375	GSM30818	22	GSE1780	11	Cu
376	GSM30819	22	GSE1780	11	Cu
377	GSM30820	22	GSE1780	11	Cu
378	GSM30821	22	GSE1780	11	Cu
379	GSM30822	22	GSE1780	11	Cu
380	GSM30823	22	GSE1780	11	Cu
381	GSM35417	23	GSE1981	1	carbon_source
382	GSM35418	23	GSE1981	1	carbon_source
383	GSM35419	23	GSE1981	1	carbon_source
384	GSM37885	24	GSE2095	10	antibiotic__toxic_agent
385	GSM37886	24	GSE2095	10	antibiotic__toxic_agent
386	GSM37887	24	GSE2095	10	antibiotic__toxic_agent
387	GSM37888	24	GSE2095	10	antibiotic__toxic_agent
388	GSM73118	25	GSE3250	7	diauxic_shift
389	GSM73119	25	GSE3250	7	diauxic_shift
390	GSM73120	25	GSE3250	7	diauxic_shift
391	GSM73121	25	GSE3250	7	diauxic_shift
392	GSM73122	25	GSE3250	7	diauxic_shift
393	GSM73123	25	GSE3250	7	diauxic_shift
394	GSM73124	25	GSE3250	7	diauxic_shift
395	GSM73125	25	GSE3250	7	diauxic_shift
396	GSM73126	25	GSE3250	7	diauxic_shift
397	GSM73127	25	GSE3250	7	diauxic_shift
398	GSM73128	25	GSE3250	7	diauxic_shift
399	GSM73129	25	GSE3250	7	diauxic_shift
400	GSM73130	25	GSE3250	7	diauxic_shift
401	GSM73131	25	GSE3250	7	diauxic_shift
402	GSM73132	25	GSE3250	7	diauxic_shift
403	GSM73133	25	GSE3250	7	diauxic_shift
404	GSM73134	25	GSE3250	7	diauxic_shift
405	GSM73135	25	GSE3250	7	diauxic_shift
406	GSM73136	25	GSE3250	7	diauxic_shift
407	GSM73137	25	GSE3250	7	diauxic_shift
408	GSM73138	25	GSE3250	7	diauxic_shift
409	GSM73139	25	GSE3250	7	diauxic_shift
410	GSM73140	25	GSE3250	7	diauxic_shift
411	GSM73141	25	GSE3250	7	diauxic_shift
412	GSM73142	25	GSE3250	7	diauxic_shift
413	GSM73143	25	GSE3250	7	diauxic_shift
414	GSM73144	25	GSE3250	7	diauxic_shift
415	GSM73145	25	GSE3250	7	diauxic_shift
416	GSM73146	25	GSE3250	7	diauxic_shift
417	GSM73147	25	GSE3250	7	diauxic_shift
418	GSM73148	25	GSE3250	7	diauxic_shift
419	GSM73149	25	GSE3250	7	diauxic_shift
420	GSM73150	25	GSE3250	7	diauxic_shift
421	GSM73151	25	GSE3250	7	diauxic_shift
422	GSM73152	25	GSE3250	7	diauxic_shift
423	GSM73153	25	GSE3250	7	diauxic_shift
424	GSM73154	25	GSE3250	7	diauxic_shift
425	GSM73155	25	GSE3250	7	diauxic_shift
426	GSM73156	25	GSE3250	7	diauxic_shift
427	GSM73157	25	GSE3250	7	diauxic_shift

428	GSM73158	25	GSE3250	7	diauxic_shift
429	GSM73159	25	GSE3250	7	diauxic_shift
430	GSM73160	25	GSE3250	7	diauxic_shift
431	GSM73252	25	GSE3250	7	diauxic_shift
432	GSM73253	25	GSE3250	7	diauxic_shift
433	GSM73254	25	GSE3250	7	diauxic_shift
434	GSM73255	25	GSE3250	7	diauxic_shift
435	GSM73256	25	GSE3250	7	diauxic_shift
436	GSM73257	25	GSE3250	7	diauxic_shift
437	GSM73258	25	GSE3250	7	diauxic_shift
438	GSM73259	25	GSE3250	7	diauxic_shift
439	GSM73260	25	GSE3250	7	diauxic_shift
440	GSM73261	25	GSE3250	7	diauxic_shift
441	GSM73262	25	GSE3250	7	diauxic_shift
442	GSM73263	25	GSE3250	7	diauxic_shift
443	GSM73264	25	GSE3250	7	diauxic_shift
444	GSM73265	25	GSE3250	7	diauxic_shift
445	GSM73266	25	GSE3250	7	diauxic_shift
446	GSM73267	25	GSE3250	7	diauxic_shift
447	GSM73268	25	GSE3250	7	diauxic_shift
448	GSM73269	25	GSE3250	7	diauxic_shift
449	GSM73270	25	GSE3250	7	diauxic_shift
450	GSM73271	25	GSE3250	7	diauxic_shift
451	GSM73272	25	GSE3250	7	diauxic_shift
452	GSM73273	25	GSE3250	7	diauxic_shift
453	GSM73274	25	GSE3250	7	diauxic_shift
454	GSM73275	25	GSE3250	7	diauxic_shift
455	GSM73276	25	GSE3250	7	diauxic_shift
456	GSM73277	25	GSE3250	7	diauxic_shift
457	GSM73278	25	GSE3250	7	diauxic_shift
458	GSM73279	25	GSE3250	7	diauxic_shift
459	GSM73280	25	GSE3250	7	diauxic_shift
460	GSM73281	25	GSE3250	7	diauxic_shift
461	GSM73282	25	GSE3250	7	diauxic_shift
462	GSM73283	25	GSE3250	7	diauxic_shift
463	GSM73284	25	GSE3250	7	diauxic_shift
464	GSM73285	25	GSE3250	7	diauxic_shift
465	GSM73286	25	GSE3250	7	diauxic_shift
466	GSM73288	25	GSE3250	7	diauxic_shift
467	GSM73289	25	GSE3250	7	diauxic_shift
468	GSM73290	25	GSE3250	7	diauxic_shift
469	GSM77341	26	GSE3437	13	sigma_factor
470	GSM77342	26	GSE3437	13	sigma_factor
471	GSM77343	26	GSE3437	13	sigma_factor
472	GSM77344	26	GSE3437	13	sigma_factor
473	GSM77345	26	GSE3437	13	sigma_factor
474	GSM77346	26	GSE3437	13	sigma_factor
475	GSM77347	26	GSE3437	13	sigma_factor
476	GSM77348	26	GSE3437	13	sigma_factor
477	GSM77349	26	GSE3437	13	sigma_factor
478	GSM77350	26	GSE3437	13	sigma_factor
479	GSM77351	26	GSE3437	13	sigma_factor
480	GSM77352	26	GSE3437	13	sigma_factor
481	GSM77353	26	GSE3437	13	sigma_factor
482	GSM77354	26	GSE3437	13	sigma_factor
483	GSM77355	26	GSE3437	13	sigma_factor
484	GSM77356	26	GSE3437	13	sigma_factor
485	GSM77357	26	GSE3437	13	sigma_factor
486	GSM77358	26	GSE3437	13	sigma_factor
487	GSM77359	26	GSE3437	13	sigma_factor
488	GSM77360	26	GSE3437	13	sigma_factor
489	GSM77361	26	GSE3437	13	sigma_factor
490	GSM82542	27	GSE3591	2	aerobic__anaerobic
491	GSM82543	27	GSE3591	2	aerobic__anaerobic
492	GSM82544	27	GSE3591	2	aerobic__anaerobic
493	GSM82545	27	GSE3591	2	aerobic__anaerobic
494	GSM82546	27	GSE3591	2	aerobic__anaerobic
495	GSM82547	27	GSE3591	2	aerobic__anaerobic

496	GSM82548	27	GSE3591	2	aerobic__anaerobic
497	GSM82549	27	GSE3591	2	aerobic__anaerobic
498	GSM82550	27	GSE3591	2	aerobic__anaerobic
499	GSM82551	27	GSE3591	2	aerobic__anaerobic
500	GSM82552	27	GSE3591	2	aerobic__anaerobic
501	GSM82553	27	GSE3591	2	aerobic__anaerobic
502	GSM82598	27	GSE3591	2	aerobic__anaerobic
503	GSM82600	27	GSE3591	2	aerobic__anaerobic
504	GSM82603	27	GSE3591	2	aerobic__anaerobic
505	GSM82604	27	GSE3591	2	aerobic__anaerobic
506	GSM82605	27	GSE3591	2	aerobic__anaerobic
507	GSM82606	27	GSE3591	2	aerobic__anaerobic
508	GSM82608	27	GSE3591	2	aerobic__anaerobic
509	GSM82610	27	GSE3591	2	aerobic__anaerobic
510	GSM82613	27	GSE3591	2	aerobic__anaerobic
511	GSM82616	27	GSE3591	2	aerobic__anaerobic
512	GSM82620	27	GSE3591	2	aerobic__anaerobic
513	GSM82621	27	GSE3591	2	aerobic__anaerobic
514	GSM83053	27	GSE3591	2	aerobic__anaerobic
515	GSM83054	27	GSE3591	2	aerobic__anaerobic
516	GSM83055	27	GSE3591	2	aerobic__anaerobic
517	GSM83056	27	GSE3591	2	aerobic__anaerobic
518	GSM83057	27	GSE3591	2	aerobic__anaerobic
519	GSM83058	27	GSE3591	2	aerobic__anaerobic
520	GSM83059	27	GSE3591	2	aerobic__anaerobic
521	GSM83060	27	GSE3591	2	aerobic__anaerobic
522	GSM83061	27	GSE3591	2	aerobic__anaerobic
523	GSM83062	27	GSE3591	2	aerobic__anaerobic
524	GSM83063	27	GSE3591	2	aerobic__anaerobic
525	GSM83064	27	GSE3591	2	aerobic__anaerobic
526	GSM83065	27	GSE3591	2	aerobic__anaerobic
527	GSM83066	27	GSE3591	2	aerobic__anaerobic
528	GSM83067	27	GSE3591	2	aerobic__anaerobic
529	GSM83068	27	GSE3591	2	aerobic__anaerobic
530	GSM83069	27	GSE3591	2	aerobic__anaerobic
531	GSM83070	27	GSE3591	2	aerobic__anaerobic
532	GSM83071	27	GSE3591	2	aerobic__anaerobic
533	GSM83072	27	GSE3591	2	aerobic__anaerobic
534	GSM83073	27	GSE3591	2	aerobic__anaerobic
535	GSM83074	27	GSE3591	2	aerobic__anaerobic
536	GSM83075	27	GSE3591	2	aerobic__anaerobic
537	GSM83076	27	GSE3591	2	aerobic__anaerobic
538	GSM83077	27	GSE3591	2	aerobic__anaerobic
539	GSM83078	27	GSE3591	2	aerobic__anaerobic
540	GSM83079	27	GSE3591	2	aerobic__anaerobic
541	GSM83080	27	GSE3591	2	aerobic__anaerobic
542	GSM83081	27	GSE3591	2	aerobic__anaerobic
543	GSM83082	27	GSE3591	2	aerobic__anaerobic
544	GSM83083	27	GSE3591	2	aerobic__anaerobic
545	GSM94002	28	GSE4112	3	DNA
546	GSM94003	28	GSE4112	3	DNA
547	GSM94004	28	GSE4112	3	DNA
548	GSM94005	28	GSE4112	3	DNA
564	GSM98717	29	GSE4321	13	sigma_factor
549	GSM98718	29	GSE4321	13	sigma_factor
550	GSM98719	29	GSE4321	13	sigma_factor
551	GSM98720	29	GSE4321	13	sigma_factor
552	GSM98721	29	GSE4321	13	sigma_factor
553	GSM98722	29	GSE4321	13	sigma_factor
554	GSM98723	29	GSE4321	13	sigma_factor
555	GSM98724	29	GSE4321	13	sigma_factor
556	GSM98725	29	GSE4321	13	sigma_factor
557	GSM98726	29	GSE4321	13	sigma_factor
558	GSM98727	29	GSE4321	13	sigma_factor
559	GSM98728	29	GSE4321	13	sigma_factor
560	GSM98729	29	GSE4321	13	sigma_factor
561	GSM98730	29	GSE4321	13	sigma_factor
562	GSM98731	29	GSE4321	13	sigma_factor

563	GSM98732	29	GSE4321	13	sigma_factor
116	GSM18235	3	GSE1121	2	aerobic__anaerobic
117	GSM18236	3	GSE1121	2	aerobic__anaerobic
118	GSM18237	3	GSE1121	2	aerobic__anaerobic
119	GSM18246	3	GSE1121	2	aerobic__anaerobic
120	GSM18247	3	GSE1121	2	aerobic__anaerobic
121	GSM18248	3	GSE1121	2	aerobic__anaerobic
122	GSM18249	3	GSE1121	2	aerobic__anaerobic
123	GSM18250	3	GSE1121	2	aerobic__anaerobic
124	GSM18251	3	GSE1121	2	aerobic__anaerobic
125	GSM18252	3	GSE1121	2	aerobic__anaerobic
126	GSM18253	3	GSE1121	2	aerobic__anaerobic
127	GSM18254	3	GSE1121	2	aerobic__anaerobic
128	GSM18255	3	GSE1121	2	aerobic__anaerobic
129	GSM18256	3	GSE1121	2	aerobic__anaerobic
130	GSM18257	3	GSE1121	2	aerobic__anaerobic
131	GSM18258	3	GSE1121	2	aerobic__anaerobic
132	GSM18259	3	GSE1121	2	aerobic__anaerobic
133	GSM18260	3	GSE1121	2	aerobic__anaerobic
134	GSM18261	3	GSE1121	2	aerobic__anaerobic
135	GSM18262	3	GSE1121	2	aerobic__anaerobic
136	GSM18263	3	GSE1121	2	aerobic__anaerobic
137	GSM18264	3	GSE1121	2	aerobic__anaerobic
138	GSM18265	3	GSE1121	2	aerobic__anaerobic
139	GSM18266	3	GSE1121	2	aerobic__anaerobic
140	GSM18271	3	GSE1121	2	aerobic__anaerobic
141	GSM18272	3	GSE1121	2	aerobic__anaerobic
142	GSM18273	3	GSE1121	2	aerobic__anaerobic
143	GSM18274	3	GSE1121	2	aerobic__anaerobic
144	GSM18275	3	GSE1121	2	aerobic__anaerobic
145	GSM18276	3	GSE1121	2	aerobic__anaerobic
146	GSM18277	3	GSE1121	2	aerobic__anaerobic
147	GSM18278	3	GSE1121	2	aerobic__anaerobic
148	GSM18279	3	GSE1121	2	aerobic__anaerobic
149	GSM18280	3	GSE1121	2	aerobic__anaerobic
150	GSM18281	3	GSE1121	2	aerobic__anaerobic
151	GSM18282	3	GSE1121	2	aerobic__anaerobic
152	GSM18283	3	GSE1121	2	aerobic__anaerobic
153	GSM18284	3	GSE1121	2	aerobic__anaerobic
154	GSM18285	3	GSE1121	2	aerobic__anaerobic
155	GSM18286	3	GSE1121	2	aerobic__anaerobic
156	GSM18287	3	GSE1121	2	aerobic__anaerobic
157	GSM18288	3	GSE1121	2	aerobic__anaerobic
158	GSM18289	3	GSE1121	2	aerobic__anaerobic
565	GSM99081	30	GSE4357	10	antibiotic__toxic_agent
566	GSM99082	30	GSE4357	10	antibiotic__toxic_agent
567	GSM99083	30	GSE4357	10	antibiotic__toxic_agent
568	GSM99084	30	GSE4357	10	antibiotic__toxic_agent
569	GSM99085	30	GSE4357	10	antibiotic__toxic_agent
570	GSM99086	30	GSE4357	10	antibiotic__toxic_agent
571	GSM99087	31	GSE4358	9	heat_cold_stress
572	GSM99088	31	GSE4358	9	heat_cold_stress
573	GSM99089	31	GSE4358	9	heat_cold_stress
574	GSM99090	31	GSE4358	9	heat_cold_stress
575	GSM99091	31	GSE4358	9	heat_cold_stress
576	GSM99092	31	GSE4358	9	heat_cold_stress
577	GSM99093	32	GSE4359	7	diauxic_shift
578	GSM99094	32	GSE4359	7	diauxic_shift
579	GSM99095	32	GSE4359	7	diauxic_shift
580	GSM99096	32	GSE4359	7	diauxic_shift
581	GSM99097	32	GSE4359	7	diauxic_shift
582	GSM99098	32	GSE4359	7	diauxic_shift
583	GSM99099	32	GSE4359	7	diauxic_shift
584	GSM99100	33	GSE4360	10	antibiotic__toxic_agent
585	GSM99101	33	GSE4360	10	antibiotic__toxic_agent
586	GSM99102	33	GSE4360	10	antibiotic__toxic_agent
587	GSM99103	33	GSE4360	10	antibiotic__toxic_agent
588	GSM99104	34	GSE4361	10	antibiotic__toxic_agent

589	GSM99105	34	GSE4361	10	antibiotic__toxic_agent
590	GSM99106	34	GSE4361	10	antibiotic__toxic_agent
591	GSM99107	34	GSE4361	10	antibiotic__toxic_agent
592	GSM99108	35	GSE4362	10	antibiotic__toxic_agent
593	GSM99109	35	GSE4362	10	antibiotic__toxic_agent
594	GSM99110	35	GSE4362	10	antibiotic__toxic_agent
595	GSM99111	35	GSE4362	10	antibiotic__toxic_agent
596	GSM99112	35	GSE4362	10	antibiotic__toxic_agent
597	GSM99113	35	GSE4362	10	antibiotic__toxic_agent
598	GSM99114	36	GSE4363	7	diauxic_shift
599	GSM99115	36	GSE4363	7	diauxic_shift
600	GSM99116	36	GSE4363	7	diauxic_shift
601	GSM99117	36	GSE4363	7	diauxic_shift
602	GSM99118	36	GSE4363	7	diauxic_shift
603	GSM99119	36	GSE4363	7	diauxic_shift
604	GSM99120	36	GSE4363	7	diauxic_shift
605	GSM99121	36	GSE4363	7	diauxic_shift
606	GSM99122	36	GSE4363	7	diauxic_shift
607	GSM99123	36	GSE4363	7	diauxic_shift
608	GSM99124	36	GSE4363	7	diauxic_shift
609	GSM99125	37	GSE4364	7	diauxic_shift
610	GSM99126	37	GSE4364	7	diauxic_shift
611	GSM99127	37	GSE4364	7	diauxic_shift
612	GSM99128	37	GSE4364	7	diauxic_shift
613	GSM99129	37	GSE4364	7	diauxic_shift
614	GSM99130	37	GSE4364	7	diauxic_shift
615	GSM99131	37	GSE4364	7	diauxic_shift
616	GSM99132	38	GSE4365	14	growth
617	GSM99133	38	GSE4365	14	growth
618	GSM99134	38	GSE4365	14	growth
619	GSM99135	38	GSE4365	14	growth
620	GSM99136	38	GSE4365	14	growth
621	GSM99137	39	GSE4366	14	growth
622	GSM99138	39	GSE4366	14	growth
623	GSM99139	39	GSE4366	14	growth
624	GSM99140	39	GSE4366	14	growth
625	GSM99141	39	GSE4366	14	growth
159	GSM37063	4	GSE2037	1	carbon_source
160	GSM37064	4	GSE2037	1	carbon_source
161	GSM37065	4	GSE2037	1	carbon_source
162	GSM37066	4	GSE2037	1	carbon_source
163	GSM37067	4	GSE2037	1	carbon_source
164	GSM37068	4	GSE2037	1	carbon_source
165	GSM37069	4	GSE2037	1	carbon_source
166	GSM37070	4	GSE2037	1	carbon_source
167	GSM37071	4	GSE2037	1	carbon_source
168	GSM37072	4	GSE2037	1	carbon_source
169	GSM37073	4	GSE2037	1	carbon_source
170	GSM37074	4	GSE2037	1	carbon_source
171	GSM37075	4	GSE2037	1	carbon_source
172	GSM37076	4	GSE2037	1	carbon_source
173	GSM37077	4	GSE2037	1	carbon_source
626	GSM99142	40	GSE4367	3	DNA
627	GSM99143	40	GSE4367	3	DNA
628	GSM99144	40	GSE4367	3	DNA
629	GSM99145	40	GSE4367	3	DNA
630	GSM99146	40	GSE4367	3	DNA
631	GSM99147	41	GSE4368	3	DNA
632	GSM99148	41	GSE4368	3	DNA
633	GSM99149	41	GSE4368	3	DNA
634	GSM99150	41	GSE4368	3	DNA
635	GSM99151	41	GSE4368	3	DNA
636	GSM99152	42	GSE4369	3	DNA
637	GSM99153	42	GSE4369	3	DNA
638	GSM99154	42	GSE4369	3	DNA
639	GSM99155	42	GSE4369	3	DNA
640	GSM99156	42	GSE4369	3	DNA
641	GSM99157	43	GSE4370	7	diauxic_shift

642	GSM99158	43	GSE4370	7	diauxic_shift
643	GSM99159	43	GSE4370	7	diauxic_shift
644	GSM99160	43	GSE4370	7	diauxic_shift
645	GSM99161	43	GSE4370	7	diauxic_shift
646	GSM99162	43	GSE4370	7	diauxic_shift
647	GSM99163	43	GSE4370	7	diauxic_shift
648	GSM99164	44	GSE4371	7	diauxic_shift
649	GSM99165	44	GSE4371	7	diauxic_shift
650	GSM99166	44	GSE4371	7	diauxic_shift
651	GSM99167	44	GSE4371	7	diauxic_shift
652	GSM99168	44	GSE4371	7	diauxic_shift
653	GSM99169	44	GSE4371	7	diauxic_shift
654	GSM99170	44	GSE4371	7	diauxic_shift
655	GSM99171	45	GSE4372	10	antibiotic__toxic_agent
656	GSM99172	45	GSE4372	10	antibiotic__toxic_agent
657	GSM99173	45	GSE4372	10	antibiotic__toxic_agent
658	GSM99174	45	GSE4372	10	antibiotic__toxic_agent
659	GSM99175	46	GSE4373	14	growth
660	GSM99176	46	GSE4373	14	growth
661	GSM99177	46	GSE4373	14	growth
662	GSM99178	46	GSE4373	14	growth
663	GSM99179	46	GSE4373	14	growth
664	GSM99180	46	GSE4373	14	growth
665	GSM99181	47	GSE4374	14	growth
666	GSM99182	47	GSE4374	14	growth
667	GSM99183	47	GSE4374	14	growth
668	GSM99184	47	GSE4374	14	growth
669	GSM99185	47	GSE4374	14	growth
670	GSM99186	47	GSE4374	14	growth
671	GSM99187	48	GSE4375	2	aerobic__anaerobic
672	GSM99188	48	GSE4375	2	aerobic__anaerobic
673	GSM99189	48	GSE4375	2	aerobic__anaerobic
674	GSM99190	48	GSE4375	2	aerobic__anaerobic
675	GSM99191	48	GSE4375	2	aerobic__anaerobic
676	GSM99192	48	GSE4375	2	aerobic__anaerobic
677	GSM99193	49	GSE4376	2	aerobic__anaerobic
678	GSM99194	49	GSE4376	2	aerobic__anaerobic
679	GSM99195	49	GSE4376	2	aerobic__anaerobic
680	GSM99196	49	GSE4376	2	aerobic__anaerobic
681	GSM99197	49	GSE4376	2	aerobic__anaerobic
682	GSM99198	49	GSE4376	2	aerobic__anaerobic
174	GSM63885	5	GSE2928	3	DNA
175	GSM63886	5	GSE2928	3	DNA
176	GSM63887	5	GSE2928	3	DNA
177	GSM63888	5	GSE2928	3	DNA
178	GSM63889	5	GSE2928	3	DNA
179	GSM63890	5	GSE2928	3	DNA
180	GSM63891	5	GSE2928	3	DNA
181	GSM63892	5	GSE2928	3	DNA
182	GSM63893	5	GSE2928	3	DNA
183	GSM63894	5	GSE2928	3	DNA
184	GSM63895	5	GSE2928	3	DNA
185	GSM63896	5	GSE2928	3	DNA
683	GSM99199	50	GSE4377	3	DNA
684	GSM99200	50	GSE4377	3	DNA
685	GSM99201	50	GSE4377	3	DNA
686	GSM99202	50	GSE4377	3	DNA
687	GSM99203	50	GSE4377	3	DNA
688	GSM99204	51	GSE4378	3	DNA
689	GSM99205	51	GSE4378	3	DNA
690	GSM99206	51	GSE4378	3	DNA
691	GSM99207	51	GSE4378	3	DNA
692	GSM99208	52	GSE4379	14	growth
693	GSM99209	52	GSE4379	14	growth
694	GSM99210	52	GSE4379	14	growth
695	GSM99211	53	GSE4380	14	growth
696	GSM99212	53	GSE4380	14	growth
697	GSM99213	53	GSE4380	14	growth

698	GSM99214	53	GSE4380	14	growth
699	GSM99215	53	GSE4380	14	growth
700	GSM99216	53	GSE4380	14	growth
701	GSM99380	54	GSE4408	3	DNA
702	GSM99384	54	GSE4408	3	DNA
703	GSM99386	54	GSE4408	3	DNA
704	GSM99389	54	GSE4408	3	DNA
705	GSM99390	54	GSE4408	3	DNA
706	GSM99391	54	GSE4408	3	DNA
707	GSM99392	54	GSE4408	3	DNA
708	GSM99393	54	GSE4408	3	DNA
709	GSM99394	54	GSE4408	3	DNA
710	GSM99395	54	GSE4408	3	DNA
711	GSM99396	54	GSE4408	3	DNA
712	GSM99397	54	GSE4408	3	DNA
713	GSM99398	54	GSE4408	3	DNA
714	GSM99399	54	GSE4408	3	DNA
715	GSM99400	54	GSE4408	3	DNA
716	GSM99401	54	GSE4408	3	DNA
717	GSM99636	55	GSE4417	9	heat_cold_stress
718	GSM99637	55	GSE4417	9	heat_cold_stress
719	GSM99638	55	GSE4417	9	heat_cold_stress
720	GSM99639	55	GSE4417	9	heat_cold_stress
721	GSM38546	56	GSE2129	3	DNA
722	GSM38547	56	GSE2129	3	DNA
723	GSM38548	56	GSE2129	3	DNA
724	GSM38549	56	GSE2129	3	DNA
725	1277	57	Courcelle_2001_Genetics	3	DNA
726	1278	57	Courcelle_2001_Genetics	3	DNA
727	1282	57	Courcelle_2001_Genetics	3	DNA
728	1285	57	Courcelle_2001_Genetics	3	DNA
729	1287	57	Courcelle_2001_Genetics	3	DNA
730	1290	57	Courcelle_2001_Genetics	3	DNA
731	1292	57	Courcelle_2001_Genetics	3	DNA
732	1908	57	Courcelle_2001_Genetics	3	DNA
733	1909	57	Courcelle_2001_Genetics	3	DNA
734	1911	57	Courcelle_2001_Genetics	3	DNA
735	1912	57	Courcelle_2001_Genetics	3	DNA
736	1913	57	Courcelle_2001_Genetics	3	DNA
737	1914	57	Courcelle_2001_Genetics	3	DNA
738	1915	57	Courcelle_2001_Genetics	3	DNA
739	1916	57	Courcelle_2001_Genetics	3	DNA
740	1585	58	Khodursky_2000_24_okt_PNAS	8	amino_acids
741	1589	58	Khodursky_2000_24_okt_PNAS	8	amino_acids
742	1592	58	Khodursky_2000_24_okt_PNAS	8	amino_acids
743	1593	58	Khodursky_2000_24_okt_PNAS	8	amino_acids
744	1595	58	Khodursky_2000_24_okt_PNAS	8	amino_acids
745	1597	58	Khodursky_2000_24_okt_PNAS	8	amino_acids
746	1637	58	Khodursky_2000_24_okt_PNAS	8	amino_acids
747	1638	58	Khodursky_2000_24_okt_PNAS	8	amino_acids
748	1639	58	Khodursky_2000_24_okt_PNAS	8	amino_acids
749	1641	58	Khodursky_2000_24_okt_PNAS	8	amino_acids
750	1642	58	Khodursky_2000_24_okt_PNAS	8	amino_acids
752	1644	58	Khodursky_2000_24_okt_PNAS	8	amino_acids
753	1646	58	Khodursky_2000_24_okt_PNAS	8	amino_acids
754	1647	58	Khodursky_2000_24_okt_PNAS	8	amino_acids
757	5265	58	Khodursky_2000_24_okt_PNAS	8	amino_acids
758	5266	58	Khodursky_2000_24_okt_PNAS	8	amino_acids
759	5268	58	Khodursky_2000_24_okt_PNAS	8	amino_acids
760	5272	58	Khodursky_2000_24_okt_PNAS	8	amino_acids
761	5273	58	Khodursky_2000_24_okt_PNAS	8	amino_acids
762	5277	58	Khodursky_2000_24_okt_PNAS	8	amino_acids
763	5278	58	Khodursky_2000_24_okt_PNAS	8	amino_acids
764	5281	58	Khodursky_2000_24_okt_PNAS	8	amino_acids
765	5284	58	Khodursky_2000_24_okt_PNAS	8	amino_acids
766	5287	58	Khodursky_2000_24_okt_PNAS	8	amino_acids
767	8455	58	Khodursky_2000_24_okt_PNAS	8	amino_acids
768	9395	58	Khodursky_2000_24_okt_PNAS	8	amino_acids

769	14829	59	Tani_2002_15_okt_PNAS	8	amino_acids
770	14830	59	Tani_2002_15_okt_PNAS	8	amino_acids
771	14831	59	Tani_2002_15_okt_PNAS	8	amino_acids
186	GSM65588	6	GSE2999	3	DNA
187	GSM65589	6	GSE2999	3	DNA
188	GSM65590	6	GSE2999	3	DNA
189	GSM65591	6	GSE2999	3	DNA
190	GSM65593	6	GSE2999	3	DNA
191	GSM65594	6	GSE2999	3	DNA
192	GSM65596	6	GSE2999	3	DNA
193	GSM65597	6	GSE2999	3	DNA
194	GSM65598	6	GSE2999	3	DNA
195	GSM65600	6	GSE2999	3	DNA
196	GSM65601	6	GSE2999	3	DNA
197	GSM65622	6	GSE2999	3	DNA
198	GSM65623	6	GSE2999	3	DNA
199	GSM65624	6	GSE2999	3	DNA
200	GSM65626	6	GSE2999	3	DNA
201	GSM65628	6	GSE2999	3	DNA
202	GSM65629	6	GSE2999	3	DNA
203	GSM65630	6	GSE2999	3	DNA
204	GSM65632	6	GSE2999	3	DNA
205	GSM65634	6	GSE2999	3	DNA
206	GSM65636	6	GSE2999	3	DNA
207	GSM65638	6	GSE2999	3	DNA
208	GSM65639	6	GSE2999	3	DNA
209	GSM65641	6	GSE2999	3	DNA
783	GSM120166	61	GSE5356	4	Fe
784	GSM120192	61	GSE5356	4	Fe
785	GSM120193	61	GSE5356	4	Fe
786	GSM120194	61	GSE5356	4	Fe
787	GSM120196	61	GSE5356	4	Fe
788	GSM120197	61	GSE5356	4	Fe
789	GSM116028	62	GSE5139	12	nitrosating_agent
790	GSM116029	62	GSE5139	12	nitrosating_agent
791	GSM116030	62	GSE5139	12	nitrosating_agent
792	GSM116031	62	GSE5139	12	nitrosating_agent
793	GSM116024	63	GSE5137	12	nitrosating_agent
794	GSM116025	63	GSE5137	12	nitrosating_agent
795	GSM116026	63	GSE5137	12	nitrosating_agent
796	GSM116027	63	GSE5137	12	nitrosating_agent
797	GSM114383	64	GSE5076	12	nitrosating_agent
798	GSM114384	64	GSE5076	12	nitrosating_agent
799	GSM114385	64	GSE5076	12	nitrosating_agent
800	GSM114386	64	GSE5076	12	nitrosating_agent
801	GSM114368	65	GSE5075	12	nitrosating_agent
802	GSM114370	65	GSE5075	12	nitrosating_agent
803	GSM114371	65	GSE5075	12	nitrosating_agent
804	GSM114372	65	GSE5075	12	nitrosating_agent
805	GSM106337	66	GSE4706	7	diauxic_shift
806	GSM106338	66	GSE4706	7	diauxic_shift
807	GSM106339	66	GSE4706	7	diauxic_shift
808	GSM106340	66	GSE4706	7	diauxic_shift
809	GSM106341	66	GSE4706	7	diauxic_shift
810	GSM106342	66	GSE4706	7	diauxic_shift
811	GSM114612	67	GSE5084	12	nitrosating_agent
812	GSM114613	67	GSE5084	12	nitrosating_agent
813	GSM114614	67	GSE5084	12	nitrosating_agent
814	GSM114615	67	GSE5084	12	nitrosating_agent
815	GSM111413	68	GSE4941	10	antibiotic__toxic_agent
816	GSM111414	68	GSE4941	10	antibiotic__toxic_agent
817	GSM111415	68	GSE4941	10	antibiotic__toxic_agent
818	GSM111416	68	GSE4941	10	antibiotic__toxic_agent
819	GSM111417	68	GSE4941	10	antibiotic__toxic_agent
820	GSM111418	68	GSE4941	10	antibiotic__toxic_agent
821	GSM111419	68	GSE4941	10	antibiotic__toxic_agent
822	GSM111420	68	GSE4941	10	antibiotic__toxic_agent
823	GSM111421	68	GSE4941	10	antibiotic__toxic_agent

824	GSM111422	68	GSE4941	10	antibiotic__toxic_agent
825	GSM111423	68	GSE4941	10	antibiotic__toxic_agent
826	GSM111424	68	GSE4941	10	antibiotic__toxic_agent
210	GSM67647	7	GSE3105	4	Fe
211	GSM67648	7	GSE3105	4	Fe
212	GSM67651	7	GSE3105	4	Fe
213	GSM67652	7	GSE3105	4	Fe
214	GSM67655	7	GSE3105	4	Fe
215	GSM67656	7	GSE3105	4	Fe
216	GSM67657	7	GSE3105	4	Fe
217	GSM67658	7	GSE3105	4	Fe
218	GSM67659	7	GSE3105	4	Fe
219	GSM67660	7	GSE3105	4	Fe
220	GSM67663	7	GSE3105	4	Fe
221	GSM67665	7	GSE3105	4	Fe
833	GSM101766	70	GSE4556	6	pH
834	GSM101767	70	GSE4556	6	pH
835	GSM101768	70	GSE4556	6	pH
836	GSM101769	70	GSE4556	6	pH
837	GSM101770	70	GSE4556	6	pH
838	GSM101771	70	GSE4556	6	pH
839	GSM101772	70	GSE4556	6	pH
840	GSM101773	70	GSE4556	6	pH
841	GSM101774	70	GSE4556	6	pH
842	GSM101775	70	GSE4556	6	pH
843	GSM101776	70	GSE4556	6	pH
844	GSM101777	70	GSE4556	6	pH
845	GSM101778	70	GSE4556	6	pH
846	GSM101779	70	GSE4556	6	pH
847	GSM101780	70	GSE4556	6	pH
848	GSM88912	71	GSE3905	5	Biofilm
849	GSM88913	71	GSE3905	5	Biofilm
850	GSM88914	71	GSE3905	5	Biofilm
851	GSM88915	71	GSE3905	5	Biofilm
852	GSM88916	71	GSE3905	5	Biofilm
853	GSM88917	71	GSE3905	5	Biofilm
854	GSM88918	71	GSE3905	5	Biofilm
855	GSM88919	71	GSE3905	5	Biofilm
856	GSM106504	72	GSE4724	8	amino_acids
857	GSM106751	72	GSE4724	8	amino_acids
858	GSM106752	72	GSE4724	8	amino_acids
859	GSM106753	72	GSE4724	8	amino_acids
860	GSM106755	72	GSE4724	8	amino_acids
861	GSM106756	72	GSE4724	8	amino_acids
862	GSM106757	72	GSE4724	8	amino_acids
863	GSM106758	72	GSE4724	8	amino_acids
864	GSM106759	72	GSE4724	8	amino_acids
865	GSM137282	73	GSE5904	5	Biofilm
866	GSM137283	73	GSE5904	5	Biofilm
867	GSM118600	74	GSE5239	15	unknown
868	GSM118601	74	GSE5239	15	unknown
869	GSM118602	74	GSE5239	15	unknown
870	GSM118603	74	GSE5239	15	unknown
871	GSM118604	74	GSE5239	15	unknown
872	GSM118605	74	GSE5239	15	unknown
873	GSM118606	74	GSE5239	15	unknown
874	GSM118607	74	GSE5239	15	unknown
875	GSM118608	74	GSE5239	15	unknown
876	GSM118609	74	GSE5239	15	unknown
877	GSM118610	74	GSE5239	15	unknown
878	GSM118611	74	GSE5239	15	unknown
879	GSM118612	74	GSE5239	15	unknown
880	GSM118613	74	GSE5239	15	unknown
881	GSM118614	74	GSE5239	15	unknown
882	GSM118615	74	GSE5239	15	unknown
883	GSM118616	74	GSE5239	15	unknown
884	GSM118617	74	GSE5239	15	unknown
885	GSM118618	74	GSE5239	15	unknown

886	GSM118619	74	GSE5239	15	unknown
887	GSM118620	74	GSE5239	15	unknown
888	GSM118621	74	GSE5239	15	unknown
889	GSM118622	74	GSE5239	15	unknown
890	GSM118623	74	GSE5239	15	unknown
222	GSM89485	8	GSE3937	5	Biofilm
223	GSM89486	8	GSE3937	5	Biofilm
224	GSM89487	8	GSE3937	5	Biofilm
225	GSM89488	8	GSE3937	5	Biofilm
226	GSM101230	9	GSE4511	6	pH
227	GSM101231	9	GSE4511	6	pH
228	GSM101232	9	GSE4511	6	pH
229	GSM101233	9	GSE4511	6	pH
230	GSM101234	9	GSE4511	6	pH
231	GSM101235	9	GSE4511	6	pH
232	GSM101236	9	GSE4511	6	pH
233	GSM101237	9	GSE4511	6	pH
234	GSM101238	9	GSE4511	6	pH
235	GSM101239	9	GSE4511	6	pH
236	GSM101240	9	GSE4511	6	pH
237	GSM101241	9	GSE4511	6	pH
238	GSM101242	9	GSE4511	6	pH
239	GSM101243	9	GSE4511	6	pH
240	GSM101244	9	GSE4511	6	pH
