# Supplementary material for: DISTILLER: a data integration framework to reveal condition dependency of complex regulons in Escherichia coli
Source: Genome Biol. 2009 Mar 6;10(3):R27. doi: 10.1186/gb-2009-10-3-r27 (PMC2690998; doi:10.1186/gb-2009-10-3-r27)
Supplement: Additional data file 4 — Detailed comparison of DISTILLER and CLR. [file gb-2009-10-3-r27-S4.pdf]

ADDITIONAL DATA FILE 4: Comparison with the Context Likelihood Relatedness and SEREND

Faith *et al*. [14] developed the "context of likelihood relatedness (CLR) framework" for network inference. This algorithm is an extension of the "relevance networks approach" where an interaction between a regulator and a target gene is predicted if the mutual information between the expression profiles of the target and the regulator exceeds a certain threshold.

In contrast to CLR and similar to DISTILLER, the SEREND data integration framework [17] does not rely on the assumption that the transcription factor should have an expression profile related to the profile of its target genes. SEREND applies an iterative classification scheme that exploits existing knowledge on regulator-target interactions in a semi-supervised way in order to predict novel interactions for these regulators. SEREND uses a co-expression score and a motif score to classify unknown interactions: a prediction between a regulator and a target gene will be ranked as highly reliable if the predicted target gene contains a motif instance similar to the motif instances in the known target genes of that regulator and if the target gene is co-expressed with the previously described targets of that regulator. Note that SEREND can also predict interactions for regulators with experimentally confirmed targets but without known binding sites: in that case it is sufficient that the novel target is co-expressed with the known targets. In contrast, DISTILLER only reports highly reliable targets for which both the co-expression and the motif constraint are fulfilled.

In the results described below we start by comparing our results with those obtained by Faith *et al*. [14] and Ernst *et al.* [17] in their original papers. Next we compare the methods by applying CLR, SEREND and DISTILLER on our expression compendium (see Supplementary File 6). As we were mainly interested in examining to what extent the methods agreed in predicting similar interactions, we only compared the interactions inferred for those 67 regulators for which a binding site is described in RegulonDB.

1. Comparison with the published CLR and SEREND networks

CLR was in the initial publication applied to an Affymetrix microarray compendium consisting of 445 arrays [14] while Ernst *et al.* [17] integrated in their initial work the same Affymetrix compendium as used by Faith *et al.* [14] with regulatory motif data. Only regulator-operon interactions are taken into account.

In comparison with the known interactions of RegulonDB, we can conclude the following (Figure S1):

- CLR identified in total 159 interactions of which 29 interactions were previously known in RegulonDB.
- DISTILLER, on the other hand, detected 732 interactions of which 454 are known.
- There were only 21 interactions reported by CLR, DISTILLER and RegulonDB.

The following comparison could be made for the novel interactions (Figure S1):

- CLR detected 130 novel interactions. The results of DISTILLER and CLR show a low overlap: only six novel interactions were identified by both methods.
- SEREND extended the known regulatory network of RegulonDB with 796 novel predictions. Of the 796 novel interactions identified by SEREND and the 278 by DISTILLER, 70 interactions were found in common by both methods, 713 were uniquely identified by SEREND and 206 were identified by DISTILLER only.
- Only four novel interactions were found in common by all three methods.

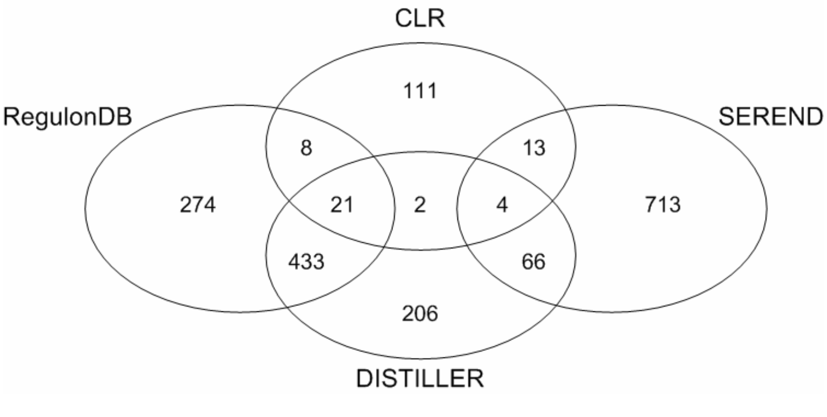

Figure S1: Venn Diagram showing the number of overlapping interactions of the networks of RegulonDB, CLR, SEREND and DISTILLER. Note that the overlap between SEREND and RegulonDB is algorithmically enforced to be 100%. Therefore only the interactions that were not previously reported in RegulonDB are visualized for SEREND.

2. Networks obtained by applying CLR and SEREND on the expression compendium used in our study

In this section, we compare our results with those obtained by CLR [14] and SEREND [17] by applying both methods on the same datasets as used in our study (the microarray compendium and regulatory motif data). As DISTILLER reports interactions between the first gene of an operon and a regulator, while CLR [14] and SEREND [17] output interactions between each operon gene and a regulator, we counted only interactions between a regulator and the first gene of an operon, in order to make the output of CLR [14] and SEREND [17] comparable. Prior to applying CLR, genes and conditions, for which too many missing values were present in the expression data, had to be deleted from the data set since CLR cannot handle missing values.

SEREND [17] assigns a prioritization for all the predictions per regulator, but does not describe a statistical way to select the number of reliable interactions per regulator (a different threshold has to be set for each regulator). For the comparison with DISTILLER we used the threshold defined in the original work of Ernst *et al.* [17]: we selected the same number of best-scoring predicted targets as the number of known targets for each regulator (referred to hereafter as the default threshold).

To determine the targets for each regulator in the CLR network, the threshold z-score has to be set. The threshold z-score can be chosen as a the default threshold used in the original publication (5.78) or by any other heuristic. For the purpose of optimizing the comparability between results we choose the z threshold as to 1) maximize the overlap between the CLR and the DISTILLER inferred networks or 2) maximize the overlap between CLR and RegulonDB networks (see also main text). Note that the latter one follows the same reasoning as in the original publication and therefore was also used to present the results in the main text.

As shown below, the overall results remained the same irrespective of how we choose the threshold: in all cases a low overall overlap in results was observed between CLR and DISTILLER on one hand and between CLR and SEREND on the other hand, whereas DISTILLER and SEREND show the largest overlap.

2.1 Default threshold of CLR

In the comparisons below we set the z threshold for CLR *equal to the one used* in the paper of Faith *et al.* (5.78) [14].

For the known interactions, the following comparison could be made (Figure S2):

- CLR reported 27 previously known interactions, while in total 382 interactions were identified. Thus 7% of the interactions identified by CLR were known interactions.
- DISTILLER detected 732 interactions of which 454 (62%) were known ones. The overlap between DISTILLER and RegulonDB is thus larger than the overlap between CLR and RegulonDB.
- Only 22 interactions were identified by CLR, DISTILLER and RegulonDB.

For the novel interactions, we can conclude the following (Figure S2):

- DISTILLER inferred 732 regulator-operon interactions for 37 different regulators of which 278 were novel.
- CLR identified 382 interactions in total. Among these 382 interactions, there were 354 novel interactions. Three of these novel interactions were also predicted by DISTILLER.
- SEREND predicted 1049 novel interactions of which, 18 were found by CLR. The overlap between DISTILLER and SEREND was the largest: 142 interactions were identified by both methods.
- Two interactions were shared by all three methods.

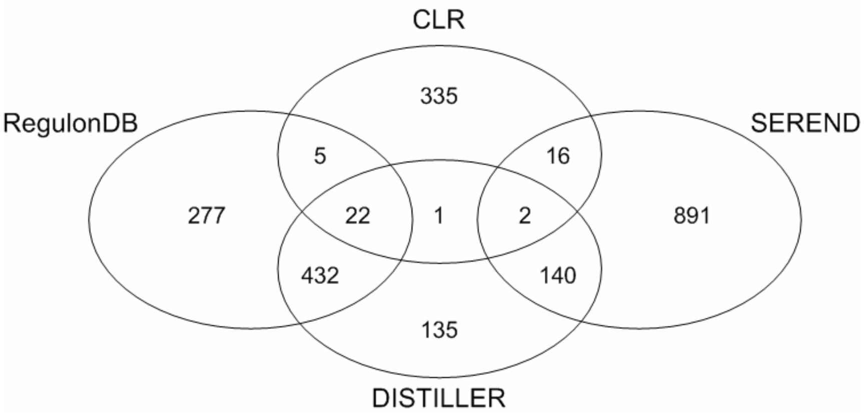

Figure S2: Venn Diagram showing the number of overlapping interactions of the networks of RegulonDB, CLR, SEREND and DISTILLER. All methods were applied on the same data sets. The default threshold z score was chosen for CLR. Note that the overlap between SEREND and RegulonDB is algorithmically enforced to be 100%. Therefore only the interactions that were not previously reported in RegulonDB are visualized for SEREND.

2.2 Maximum overlap between CLR and DISTILLER

In the comparison below we set the z threshold for CLR equal to the value that *maximizes the overlap in results between DISTILLER and CLR.*

To obtain this z-score threshold, we calculated the overlap of the obtained network with the DISTILLER inferred network for varying z-score thresholds using the Jaccard coefficient (see Figure S3). When we only bring into account the interactions between a regulator and the first gene of an operon, the CLR threshold z-score resulting in the highest overlap with the DISTILLER network was equal to 5.

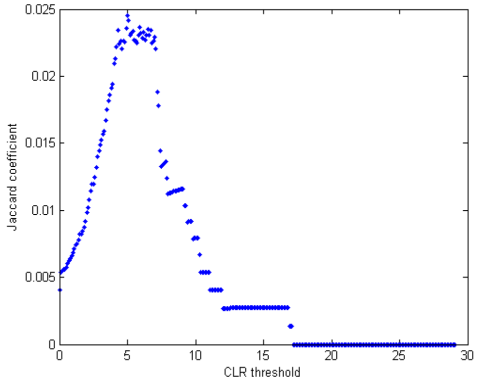

Figure S3 : Jaccard coefficients for the comparison of the DISTILLER inferred network with the networks obtained by applying different CLR thresholds.

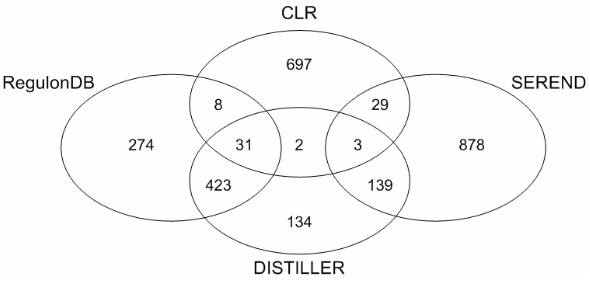

Figure S4: Venn Diagram showing the number of overlapping interactions of the networks of RegulonDB, CLR, SEREND and DISTILLER. Note that the overlap between SEREND and RegulonDB is algorithmically enforced to be 100%. Therefore only the interactions that were not previously reported in RegulonDB are visualized for SEREND.

Using this setting, the following conclusions could be drawn (see Figure S4):

- Of the 770 interactions that were inferred by CLR, 39 interactions (5% of the total number of interactions) were previously known interactions.
- DISTILLER detected 454 (62% of the total number of interactions) known interactions in total. DISTILLER can thus recover more of the known interactions than CLR.
- In total, 31 interactions were identified by CLR, DISTILLER and RegulonDB.

For the novel interactions, we can conclude the following (see Figure S4):

- DISTILLER inferred 454 previously reported interactions and 278 novel ones.
- CLR identified a total of 770 interactions of which 721 were novel ones. Five novel interactions were also predicted by DISTILLER.
- SEREND identified 1049 novel interactions in total. Of these 1049 interactions, CLR predicted 32 interactions The overlap between DISTILLER and SEREND was the largest: 142 interactions were identified by both methods.
- Only 3 novel interactions were shared by all three methods.

2.3 Maximum overlap between CLR and RegulonDB

In the comparisons below we set the z threshold for CLR equal to the value that maximized the overlap in results between CLR and RegulonDB.

To obtain this z-score, we calculated the overlap of the obtained network with the RegulonDB known network for varying z-score thresholds using the Jaccard coefficient (see Figure S5). When calculating the overlap between both networks, we bring only interactions between a regulator and the first gene of operon into account. The CLR threshold z-score resulting in the highest overlap with the RegulonDB network was 4.3.

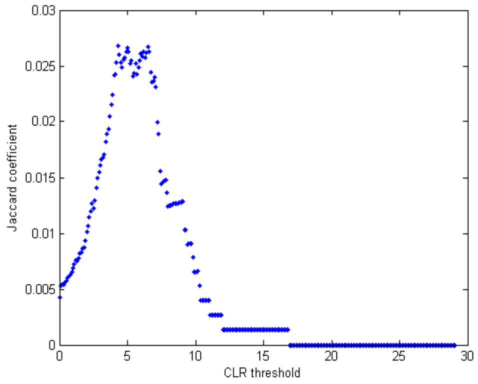

Figure S5: Jaccard coefficients for the comparison of the RegulonDB network with the networks obtained by applying different CLR thresholds.

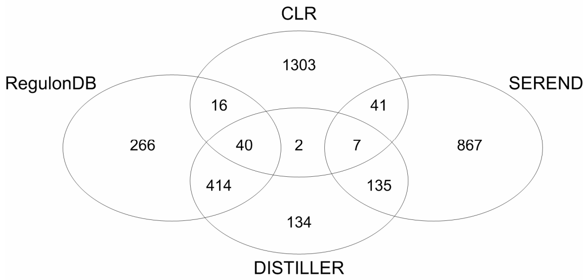

Figure S6: Venn Diagram showing the number of overlapping interactions of the networks of RegulonDB, CLR, SEREND and DISTILLER. Note that the overlap between SEREND and RegulonDB is algorithmically enforced to be 100%. Therefore only the interactions that were not previously reported in RegulonDB are visualized for SEREND.

Using this setting, the following conclusions could be made for the known interactions (Figure S6):

- CLR inferred 1409 interactions in total, of which 56 interactions (4% of the total number of interactions inferred by CLR) were previously known interactions.
- DISTILLER detected 454 (62% of the total number of interactions reported by DISTILLER) known interactions in total. DISTILLER could thus identify more known interactions than CLR.
- In total, 40 interactions were reported by CLR, DISTILLER and RegulonDB.

For the novel interactions, we can conclude the following (Figure S6):

- DISTILLER inferred 454 previously reported interactions and 278 novel ones.
- CLR identified 1409 interactions amongst which 1353 were novel ones. The overlap between the DISTILLER and the CLR network was again rather low: when considering the novel interactions only 9 interactions were common between DISTILLER and CLR.
- SEREND identified 1049 novel interactions in total of which 48 were also identified by CLR. The overlap between SEREND and DISTILLER interactions was the largest: 142 interactions were identified by both methods.
- All methods shared 7 interactions in common.

Conclusion

Irrespective of whether we compared the three methods on the same datasets (using different ways to optimize the thresholds for CLR) or whether we compared previously published results obtained by each of these methods on a different *E. coli* dataset, the results remain the same. In general the overlap between all three methods is rather low. DISTLLER agrees most with SEREND and the lowest overlap between the results was observed in the comparison between DISTILLER and CLR. This is to be expected as both DISTILLER AND SEREND are integrative approaches designed to make less but more reliable predictions while CLR makes use of completely different underlying assumptions.
