# Supplementary material for: DISTILLER: a data integration framework to reveal condition dependency of complex regulons in Escherichia coli
Source: Genome Biol. 2009 Mar 6;10(3):R27. doi: 10.1186/gb-2009-10-3-r27 (PMC2690998; doi:10.1186/gb-2009-10-3-r27)
Supplement: Additional data file 5 — Comparison of CLR, SEREND and DISTILLER with five available ChIP-chip experiments. [file gb-2009-10-3-r27-S5.pdf]

ADDITIONAL DATA FILE 5: Comparison with ChIP-chip experiments

CLR [14] and SEREND [17] were applied on our data sets (microarray compendium and regulatory motif data). To determine the number of targets for each regulator in CLR [14], we had to choose a threshold z score. We choose a threshold of 4.3 such that a maximum overlap between the network of CLR [14] and RegulonDB [3] was obtained (see also main text and Additional file 4). To determine the targets of each regulator in SEREND [17], we used the threshold defined in the original work of Ernst *et al.* [17]: we selected the same number of best-scoring predicted targets as the number of known targets for each regulator.

A comparison of CLR [14], SEREND [17] and DISTILLER with the available ChIP-chip data [24,36,37] is shown in Table S1 and Figure S1. From the table it is clear that ChIP-chip experiments are not able to recover many of the known RegulonDB interactions (see remark below).

For the remainder of the comparisons we focused on 'novel interactions' as only for these interactions a fair comparison between supervised (the ones that build upon RegulonDB) and unsupervised methods (the ones that are independent from RegulonDB) can be made. The ChIP-chip data were subsequently considered as our golden standard: novel interactions identified by CLR, SEREND or DISTILLER and confirmed by ChIP-chip are considered true positives, whereas novel interactions that were not confirmed by ChIP-chip are considered false positives. Interactions found by ChIP-chip but not detected by the methods were considered false negatives.

The recall is then defined as the ratio of the number of ChIP-chip interactions that were identified by either CLR, SEREND or DISTILLER versus the total number of ChIP-chip interactions:

$$\frac{TP}{TP + FN}$$

Where TP are the true positives and FN are the false negatives. The TP+FN is the total number of interactions in the ChIP-chip data (not confirmed in RegulonDB, since we only compare the novel interactions).

On average, CLR [14] does not recover many of the ChIP-chip confirmed interactions and has thus the lowest recall. The recall of SEREND [17] on the other hand seems to be the highest. DISTILLER scores in between.

The precision is defined as the ratio of the number of ChIP-chip confirmed interactions that were identified by CLR, SEREND or DISTILLER versus the total number of predictions by either CLR, SEREND or DISTILLER:

$$\frac{TP}{TP + FP}$$

Where TP are the true positives and FP are the false positives. The TP + FP are the total number of predictions that were inferred by CLR, SEREND or DISTILLER.

SEREND [17] predicts many more interactions than DISTILLER. For instance, SEREND [17] reports 212 novel interactions for CRP of which 9 were also present in the ChIP-chip data, while DISTILLER reports only 62 novel interactions for CRP of which six were identified by the ChIP-chip data. So the ratio of the ChIP-chip confirmed predictions made by SEREND (TP) versus the total number of predictions (TP+FP) at the used precision-recall tradeoff is much lower for SEREND than for DISTILLER. The higher recall of SEREND is thus obtained at the expense of a lower precision. CLR predicted in general less interactions for each regulator (lower recall), but the precision is also low, as not many ChIP-chip confirmed interactions could be identified by CLR.

When using the default settings, all three algorithms thus work at a different precision-recall trade-off. In order to obtain a more fair comparison in terms of this underlying precision-recall trade-off, we varied the threshold for the predictions in SEREND [17] and CLR [14]. For each regulator for which ChIP-chip data were available, a precision-recall curve was as such obtained (see Figure S1). Subsequently, we could identify the parameter settings of SEREND and CLR that result in the same precision/recall trade off as DISTILLER. From this analysis it is clear that for the same recall as DISTILLER, CLR shows a lower precision for all regulators, while SEREND shows a similar or slightly lower precision for most regulators. This confirms the general comparison by the different methods on our dataset: the overlap between the two integrative methods DISTILLER and SEREND is larger than the overlap with CLR. The integrative approaches are designed to make less but more reliable predictions. Note that the results of this comparison, although indicative should be treated with caution (see remark below).

Table S1: Comparison of interactions identified by ChIP-chip experiments, CLR (threshold z score = 4.3), SEREND and DISTILLER for five global regulators.

The identified interactions for each method were categorized according to whether these interactions were known interactions (Confirmed RegulonDB) or novel interactions (Predictions) as compared to RegulonDB. In addition it is indicated whether (ChIP-chip) or not (Not ChIP-chip) the interactions were found in a corresponding ChIP-chip experiment. The recall (TP/TP+FN) and precision (TP/TP+FP) were calculated using the ChIP-chip data as a golden standard. Interactions identified by either CLR, SEREND or DISTILLER and confirmed by a ChIP-chip experiment were considered to be true positive interactions (TP); interactions confirmed by a ChIP-chip experiment but not identified by either CLR, SEREND or DISTILLER were considered false negatives (FN); interactions identified by either CLR, SEREND or DISTILLER but not confirmed in a ChIP-chip experiment were considered false positives (FP). Note that since all interactions of RegulonDB are recovered by SEREND by definition (algorithmic consequence of using RegulonDB as a training set), we only showed the "novel" interactions predicted by SEREND.

	Confirmed RegulonDB					Predictions				
	Not ChIP-chip	ChIP-chip	Total	Recall	Precision	Not ChIP-chip	ChIP-chip	Total	Recall	Precision
**FNR**										
ChIP-chip	/	21	21			/	73	73		
CLR	0	0	0	0	0	14	0	14	0	0
SEREND	/	/	/	/	/	76	19	95	0,26	0,20
DISTILLER	29	19	48	0,90	0,40	21	4	25	0,055	0,16
**CRP**										
ChIP-chip	/	31	31			/	57	57		
CLR	3	0	3	0	0	23	0	23	0	0
SEREND	/	/	/	/	/	203	9	212	0,16	0,042
DISTILLER	90	21	111	0,68	0,19	57	6	63	0,11	0,095
**FIS**										
ChIP-chip	/	5	5			/	179	179		
CLR	1	0	1	0	0	59	4	63	0,022	0,06
SEREND	/	/	/	/	/	33	4	37	0,022	0,11
DISTILLER	18	3	21	0,60	0,14	17	3	20	0,017	0,15
**H-NS**										
ChIP-chip	/	0	0			/	82	82		
CLR	0	0	0	0	0	26	0	26	0	0
SEREND	/	/	/	/	/	4	0	4	0	0
DISTILLER	0	0	0	0	0	0	0	0	0	0
**IHF**										
ChIP-chip	/	8	8			/	110	110		
CLR	4	0	4	0	0	67	4	71	0,036	0,056
SEREND	/	/	/	/	/	79	10	89	0,091	0,11
DISTILLER	32	7	39	0,88	0,18	14	4	18	0,036	0,22

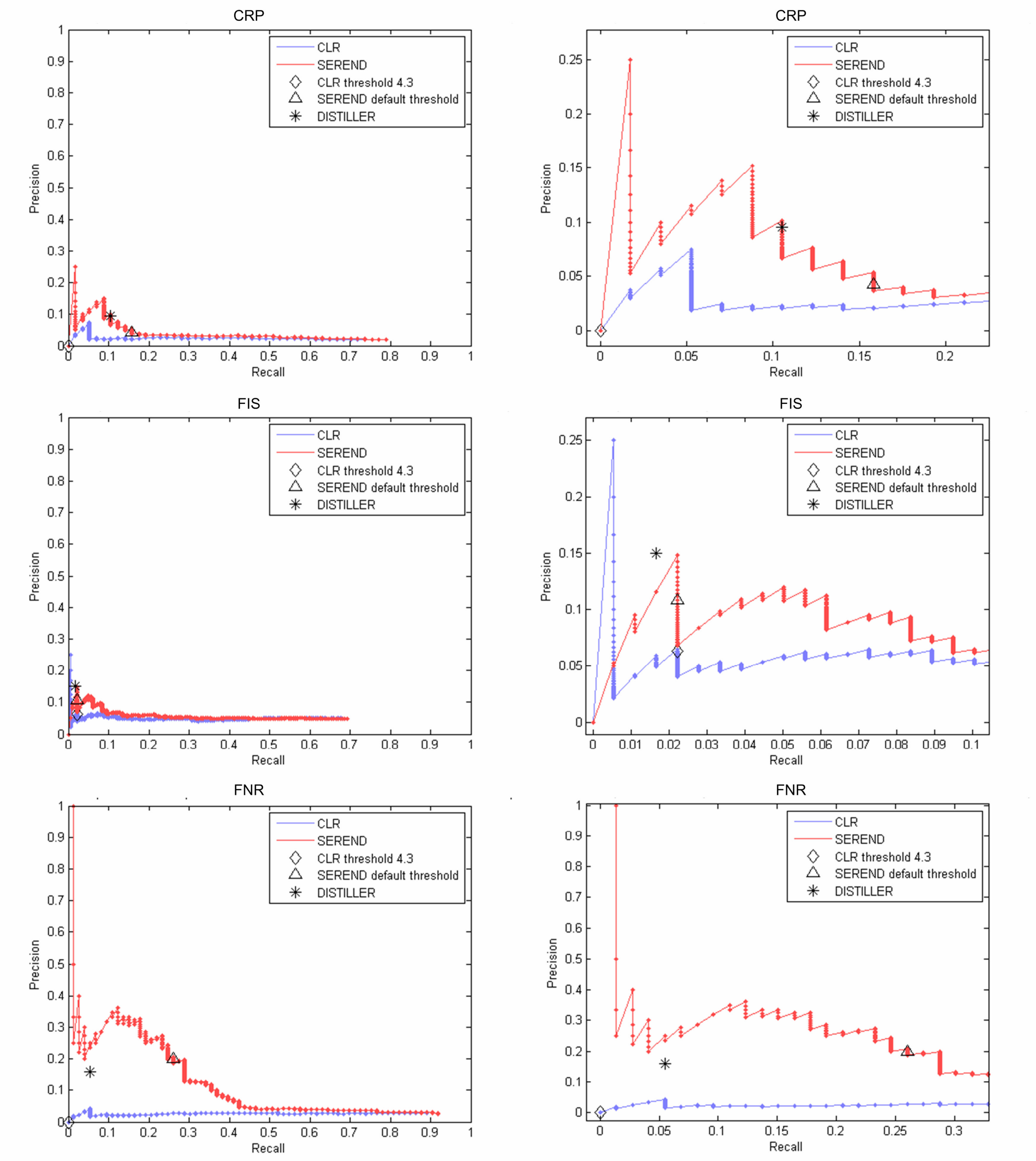
CRP
CLR
SEREND
CLR threshold 4.3
SEREND default threshold
DISTILLER
Precision
Recall
CRP
CLR
SEREND
CLR threshold 4.3
SEREND default threshold
DISTILLER
Precision
Recall
FIS
CLR
SEREND
CLR threshold 4.3
SEREND default threshold
DISTILLER
Precision
Recall
FIS
CLR
SEREND
CLR threshold 4.3
SEREND default threshold
DISTILLER
Precision
Recall
FNR
CLR
SEREND
CLR threshold 4.3
SEREND default threshold
DISTILLER
Precision
Recall
FNR
CLR
SEREND
CLR threshold 4.3
SEREND default threshold
DISTILLER
Precision
Recall

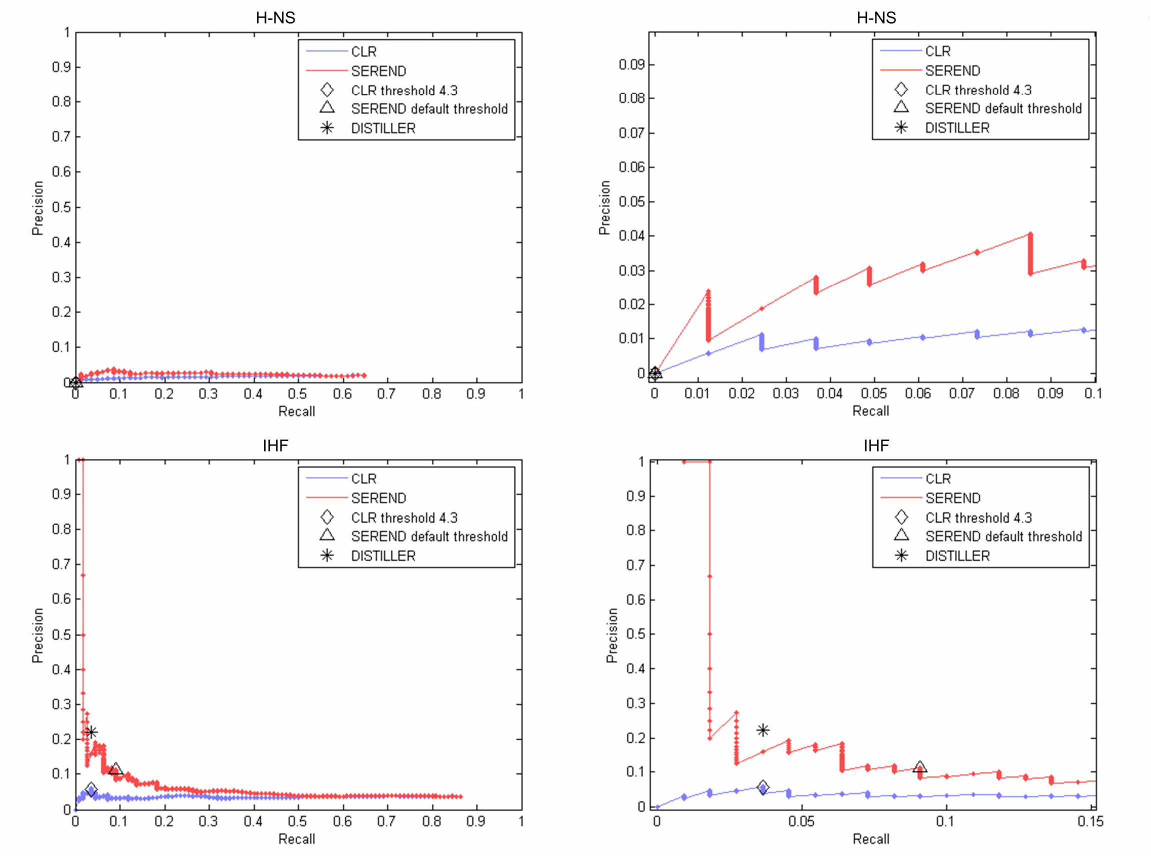

Figure S1: Recall (TP/(TP+FN) versus precision (TP/(TP+FP) for the predictions made by SEREND, CLR and DISTLLER using ChIP-chip data as golden standard (TP are those interactions detected by the inference method and confirmed by ChIP-Chip; FN are those interactions identified by the ChIP-chip experiment but not by the inference method; FP are those interactions detected by the inference method but not confirmed by ChIP-Chip; TN are those interactions not detected by the inference method and not confirmed by ChIP-Chip). The recall and precision were calculated starting with the best-ranked prediction of SEREND and by adding the next best ranked predictions one by one. The same procedure was applied on the results of CLR where predictions were ranked according to their z score. For each regulator that was measured in a ChIP-chip experiment, a plot is shown. The right panel shows plots that zoom in on the plots in the left panel. The recall-precision trade-off are indicated for the SEREND default values, and for three z threshold values for CLR used on the total dataset. For DISTILLER only one precision and recall is given (the one used in this work). For most examined regulators (except FNR), at the working regime used for DISTILLER, DISTILLER has the best precision for the corresponding recall.

Remark

Although ChIP-chip analysis is a valuable technique to screen for direct physical interactions between a regulator and its target genes, ChIP-chip data may contain a significant number of false-positives and false-negatives. False-positives might arise due to, for instance, biological noise: often a TF binds to the genome but seems to be physiologically inactive (not inducing expression). On the other hand, false-negatives may be present in ChIP-chip data because of, for instance, the condition-dependency of the regulator binding. For these reasons, although ChIP-chip inferred interactions may yield low overlap with known interactions from RegulonDB [1] they are the only benchmark resource currently available that is independent from RegulonDB. So using them for benchmarking is useful but should be done with caution (in order not overestimate its value as golden standard).
