# Supplementary material for: DISTILLER: a data integration framework to reveal condition dependency of complex regulons in Escherichia coli
Source: Genome Biol. 2009 Mar 6;10(3):R27. doi: 10.1186/gb-2009-10-3-r27 (PMC2690998; doi:10.1186/gb-2009-10-3-r27)
Supplement: Additional data file 6 — The microarray compendium and the normalization of the microarrays in detail. [file gb-2009-10-3-r27-S6.pdf]

ADDITIONAL DATA FILE 6: the microarray compendium

Our cross-platform compendium contains a large collection of publicly available microarray. The data were collected from the three major microarray databases: Stanford Microarray Database (SMD) [39], Gene Expression Omnibus (GEO) [40] and ArrayExpress (AE) [41]. Additionally, we added four microarray experiments described in literature that were available as supplementary information. After removing redundant information in the microarray databases, we obtained a total of 870 microarrays (data available upon request). Table S1 gives an overview of the contribution of the different platforms to the compendium. Conditional categories were assigned based on manual curation: descriptions of each experiment as available in the database were combined with those of the corresponding publications. A full description of the microarray compendium can be found in Table S2 and on the supplementary website.

Preprocessing of the microarray compendium

To make the data comparable between different experimental conditions and platforms the following normalization procedures were conducted. If possible, raw intensities were preferred as data source over normalized data provided by the public repository. Dual-channel data were loess fitted to remove nonlinear, dye-related discrepancies [63]. No background correction procedures were performed to avoid an increase in expression logratio variance for lower, less reliable intensity levels. Whenever raw data were available, single-channel data were first normalized per experiment with RMA [64]. Logratios were then created for the single-channel data in order to combine them with the dual channel measurements. For each single-channel array, expression logratios were computed by comparing the normalized values against an artificial reference array. This artificial reference array was constructed on a per experiment basis by taking the median expression of each gene across all arrays in the corresponding experiment. When deemed necessary (e.g. experiments normalized by MAS5.0 for which the raw data was not available), a loess fit was performed on these logratios. To ensure that the artificial reference was not altered by this intensity dependent non-linear rescaling, the artificial reference expression levels were chosen for the average log intensity (instead of the mean expression levels of the respective array and the artificial reference). To ensure comparability between arrays with a different reference, gene expression profiles were median centered across arrays that share the same reference. An additional variance rescaling of the gene expression profiles was performed to render genes with differing magnitudes of expression changes more comparable.

Per array percentile ranks (ranging from zero to one) of the normalized logratios were used as input for DISTILLER. This rescaling was done because random sets of genes can show a small bandwidth without being co-expressed. In each measured condition, the genes were ranked such that genes with the lowest expression values obtain the highest rank. Genes that do not significantly change their expression levels will tend to produce ranks randomly within the bulk of these rank scores, so that the difference between the maximum and minimum values (or the box threshold used by DISTILLER; see additional file 7) between two random genes that do not change their expression can become rather large. We thus take differences between the maximum and minimum "ranks" instead of the differences of the absolute expression values. Our criterion will therefore select genes that are highly over- or under-expressed: although their expression values might be quite different, their rescaled values will be very close in comparison with random genes. The two most over-expressed genes have a rank difference (bandwidth score) equal to one, whereas two silent genes may have a rank difference that could be any number between one and, say, 3000 (assuming 3000 genes are do not respond to the environmental stimulus).

Analysis of the obtained modules indicated that the array composition of the retrieved modules is not biased towards arrays from a specific platform, indicating a correct preprocessing of the microarray compendium: the average percentage of arrays from a particular platform in a module approximates the overall percentage of arrays of that platform in the total compendium. Only 6 arrays (from 5 different experiments) are not included in any module.

Table S1: Overview of the contribution of the different platforms and data sources to the compendium.

		data source			
		**GEO**	**SMD**	**AE**	**Literature**
**single channel**	**Affymetrix**	183	-	-	115
	**P33**	46	-	-	63
**dual channel**	**spotted DNA/cDNA**	278	44	12	3
	**spotted oligonucleotide**	110	-	16	-

Table S2: Overview of the different experiments in the expression compendium

Experiment ID	Experiment	No. of arrays	Article	Data type	Data source
1	Palsson_2005_Genome_Res	99	16204189	affy	Literature
10	Chang_2002_Mol_Biol	63	12123445	P33	Literature
11	GSE2827	6	16549663	P33	GEO
12	GSE533	12	12672900	P33	GEO
13	E-MEXP-222	6	15659689	oligo	AE
14	E-MEXP-244	4	NO	oligo	AE
15	E-MEXP-245	6	NO	oligo	AE
18	E-MEXP-267	12	NO	cDNA	AE
19	Blattner_2005_Genome_Res	3	14597655	cDNA	Literature
2	Gossett_2005_J_of_Bact	16	15659676	affy	Literature
20	GSE33	16	11967071	P33	GEO
21	GSE1421	3	15466047	oligo	GEO
22	GSE1780	9	15817786	oligo	GEO
23	GSE1981	3	15821913	oligo	GEO
24	GSE2095	4	15647275	oligo	GEO
25	GSE3250	81	16141204	cDNA	GEO
26	GSE3437	21	16336047	cDNA	GEO
27	GSE3591	55	16377617	oligo	GEO
28	GSE4112	4	14526013	cDNA	GEO
29	GSE4321	16	16818608	cDNA	GEO
3	GSE1121	43	15129285	affy	GEO
30	GSE4357	6	16626502	cDNA	GEO
31	GSE4358	6	16626502	cDNA	GEO
32	GSE4359	7	16626502	cDNA	GEO
33	GSE4360	4	16626502	cDNA	GEO
34	GSE4361	4	16626502	cDNA	GEO
35	GSE4362	6	16626502	cDNA	GEO
36	GSE4363	11	16626502	cDNA	GEO
37	GSE4364	7	16626502	cDNA	GEO
38	GSE4365	5	16626502	cDNA	GEO
39	GSE4366	5	16626502	cDNA	GEO
4	GSE2037	15	15705577	affy	GEO
40	GSE4367	5	16626502	cDNA	GEO
41	GSE4368	5	16626502	cDNA	GEO
42	GSE4369	5	16626502	cDNA	GEO
43	GSE4370	7	16626502	cDNA	GEO
44	GSE4371	7	16626502	cDNA	GEO
45	GSE4372	4	16626502	cDNA	GEO
46	GSE4373	6	16626502	cDNA	GEO
47	GSE4374	6	16626502	cDNA	GEO
48	GSE4375	6	16626502	cDNA	GEO
49	GSE4376	6	16626502	cDNA	GEO
5	GSE2928	12	16199573	affy	GEO
50	GSE4377	5	16626502	cDNA	GEO
51	GSE4378	4	16626502	cDNA	GEO
52	GSE4379	3	16626502	cDNA	GEO
53	GSE4380	6	16626502	cDNA	GEO
54	GSE4408	16	17009874	cDNA	GEO

55	GSE4417	4	16626502	cDNA	GEO
56	GSE2129	4	15647275	oligo	GEO
57	Courcelle_2001_Genetics	15	11333217	cDNA	SMD
58	Khodursky_2000_24_okt_PNAS	26	11027315	cDNA	SMD
59	Tani_2002_15_okt_PNAS	3	12374860	cDNA	SMD
6	GSE2999	24	NO	affy	GEO
61	GSE5356	6	16952965	oligo	GEO
62	GSE5139	4	17189370	oligo	GEO
63	GSE5137	4	17189370	oligo	GEO
64	GSE5076	4	17189370	oligo	GEO
65	GSE5075	4	17189370	oligo	GEO
66	GSE4706	6	16804185	oligo	GEO
67	GSE5084	4	17189370	oligo	GEO
68	GSE4941	12	16849805	P33	GEO
7	GSE3105	12	16199566	affy	GEO
70	GSE4556	15	17026754	affy	GEO
71	GSE3905	8	17222132	affy	GEO
72	GSE4724	9	17074904	affy	GEO
73	GSE5904	2	NO	affy	GEO
74	GSE5239	24	NO	affy	GEO
8	GSE3937	4	16597943	affy	GEO
9	GSE4511	15	15601715	affy	GEO
