# Supplementary material for: DISTILLER: a data integration framework to reveal condition dependency of complex regulons in Escherichia coli
Source: Genome Biol. 2009 Mar 6;10(3):R27. doi: 10.1186/gb-2009-10-3-r27 (PMC2690998; doi:10.1186/gb-2009-10-3-r27)
Supplement: Additional data file 7 — The DISTILLER algorithm and its parameter settings. [file gb-2009-10-3-r27-S7.pdf]

ADDITIONAL DATA FILE 7: DISTILLER algorithm

In this study we developed ‘DISTILLER’ (Data Integration System To Identify Links in Expression Regulation) a data integration framework that searches for transcriptional modules by combining expression data with information on the direct interaction between a regulator and its corresponding target genes. The inferred modules consist of co-expressed genes, their corresponding regulator(s), and the experimental conditions of the expression data for which the selected genes are co-expressed.

Our methodology consists of three steps (see Figure S1): (1) the identification of seed modules; (2) the reduction of the set of all seed modules to a manageable set of non-redundant (small overlap) and significant (association in the data cannot be explained by chance) seed modules; and (3) the extension of the thus obtained seed modules with additional genes.

Although our approach can be extended to any number of input matrices (data sources), we used two input matrices to generate the results in this paper:

- An appropriately normalized expression data compendium **A** with dimensions $N_G \times N_C$, where N_G indicates the total number of genes in the compendium and N_C the total number of conditions.
- A binary ‘regulatory motif matrix’ **R** (input interaction matrix) with dimensions $N_G \times N_R$, where N_R is the total number of regulators for which motif data is available. Each element r_{ip} of this matrix indicates whether the upstream region of a specific gene i contains a statistically significant motif instance of the known regulatory motif model of that specific regulator p.

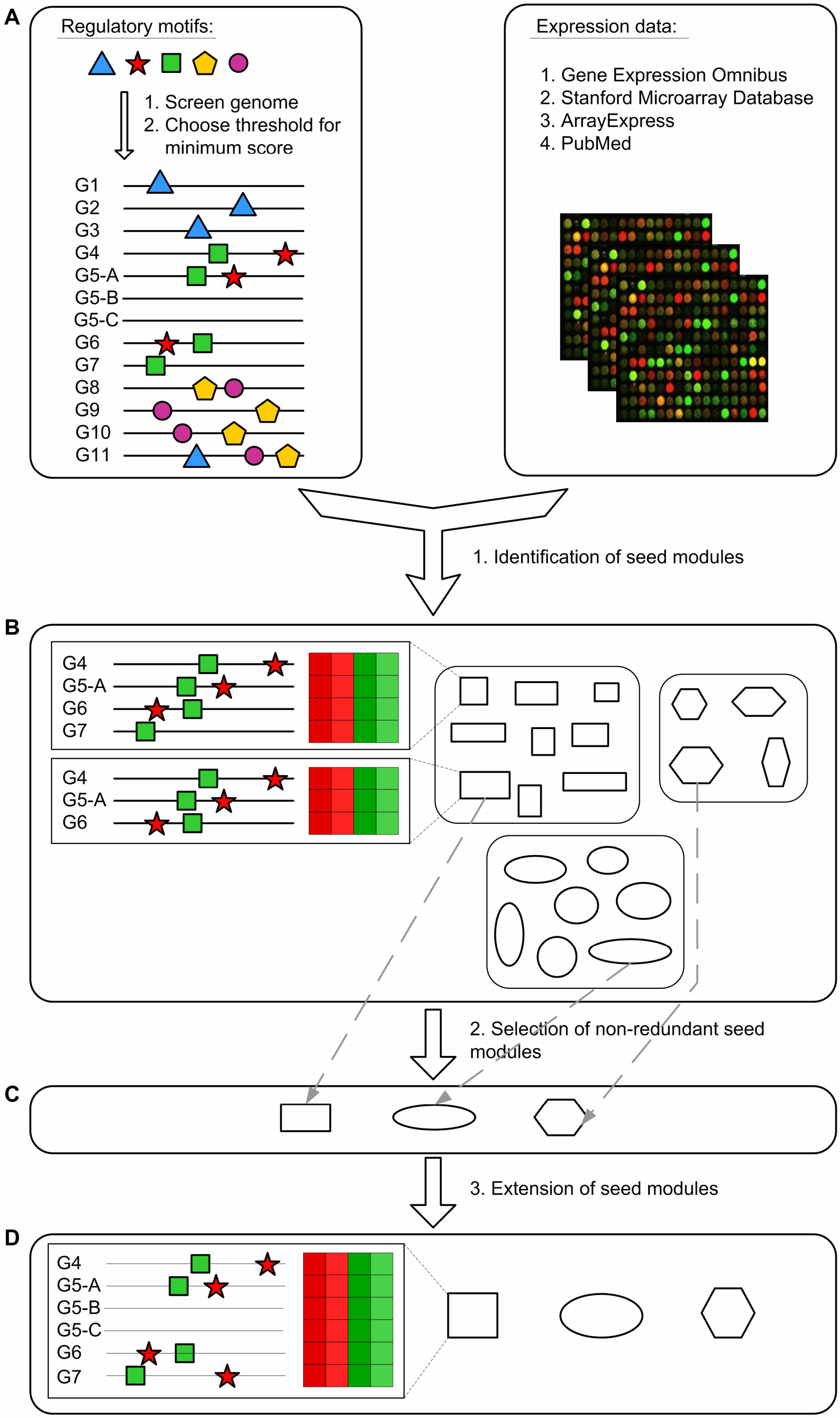

Figure S1: caption see page 3**.**

Figure S1: The different steps of the DISTILLER algorithm.

A) Motif matrices (indicated by a triangle, star, square, pentagon and circle) obtained from RegulonDB were used to screen the genome of E. coli. As such a score was assigned to each motif-gene combination. If this score was larger than a specific threshold, we assume that the motif is present in the promoter region of a gene. Microarray experiments were collected from the three large microarray databases [39-41] and subsequently grouped in a compendium. These two data sources are the input for DISTILLER. **B)** In the first step, DISTILLER will search the seed modules. A seed module is a set of genes that is co-expressed in a subset of conditions and that have minimum one motif in their promoter regions in common. All possible seed modules can be identified very efficiently by using an algorithm based on CHARM [44]. Because of noise in the data, the output may consist of partially redundant modules, for instance, modules differing from each other in a gene only. In our example, three groups of partially redundant modules were identified (indicated by identical symbols of different sizes). Two modules of one such group are shown: the first module contains four genes that have one motif in common (the green square), whereas the second module consists of only three genes that share two motifs (green square and red star). These modules can thus be considered to be partially redundant. **C)** In the module selection step, the most interesting modules are therefore selected one by one depending on their statistical significance and the extent to which they contribute to the covering of the complete solution space (and thus do not overlap with modules that had already been selected). In our example, the most interesting module of each group was selected. For instance, the module containing three genes and two motifs was selected. **D)** In the final module extension step, additional genes are recruited that did not pass the stringent seed discovery step but should be considered part of the module (e.g. downstream operon genes). The module in the example could as such be extended with the operon genes (G5-B and G5-C) that belong to the same operon as gene G5-A. In addition, gene G7 was recruited with the more relaxed criteria, like a lower threshold for the motif score, caused the assignment of the two motifs (green square and red star) to this gene G7.

2.1 Seed modules

Let $\mathbf{g}^{(m)}$ be the set of gene indices that correspond to genes in module *m*. DISTILLER initially identifies seed modules that satisfy three constraints:

(C_G) The module should contain a minimum number *G* of genes (i.e. gene content threshold), or $\left|\mathbf{g}^{(m)}\right| \geq G$.

(C_R) All genes $\mathbf{g}^{(m)}$ in the module should contain motif instances for a sufficient number *R* of common, a priori unspecified regulators. Let **1** represent an all-ones vector of appropriate dimensions. Then, this constraint can be formulated mathematically as $\left|\left\{p \mid \mathbf{1}^{\mathbf{T}}\mathbf{R}_{\mathbf{g}^{(m)},p} = \left|\mathbf{g}^{(m)}\right|\right\}\right| \geq R$.

(C_C) All genes $\mathbf{g}^{(m)}$ in the module should be significantly co-expressed in a sufficiently large, a priori unspecified set of experimental conditions. We indicate this required number of conditions by *C*. In order to calculate the maximal valid condition set $\boldsymbol{c}^{(m)}$ given a certain gene set $\boldsymbol{g}^{(m)}$, we first compute the difference between the largest and smallest expression levels in the gene set for each of the conditions: $BW_j = \max(\mathbf{A}_{\boldsymbol{g}^{(m)},j}) - \min(\boldsymbol{A}_{\boldsymbol{g}^{(m)},j})$. We call BW_j the bandwidth for condition *j*. Subsequently, these bandwidths are sorted in increasing order to obtain a sorted bandwidth sequence $BW_j^{(s)}$ that satisfies $BW_{j-1}^{(s)} \leq BW_j^{(s)} \leq BW_{j+1}^{(s)}$ (see Figure S2). The sequence $BW_j^{(s)}$ is then compared with a pre-specified threshold bandwidth sequence $BW_j^{(th)}$ that is sorted as well: $BW_{j-1}^{(th)} \leq BW_j^{(th)} \leq BW_{j+1}^{(th)}$. Gene set $\mathbf{g}^{(m)}$ is said to be co-expressed in C_{max} conditions if the sorted bandwidth sequence $BW_j^{(s)}$ is completely within the threshold bandwidth sequence $BW_j^{(th)}$ for $1 \leq j \leq C_{\max}$, i.e. if $BW_j^{(s)} \leq BW_j^{(th)}$ for $1 \leq j \leq C_{\max}$. Constraint C_C is satisfied if this property holds for $C_{\max} \geq C$. There are various ways to choose the pre-specified bandwidth threshold sequence. One data-driven approach is described below.

A naive exhaustive search for valid modules as defined above would require checking all possible combinations of genes, motif instances, and experimental conditions. This is unfeasible for data sets of any reasonable size. This issue is solved by using the CHARM algorithm [44].

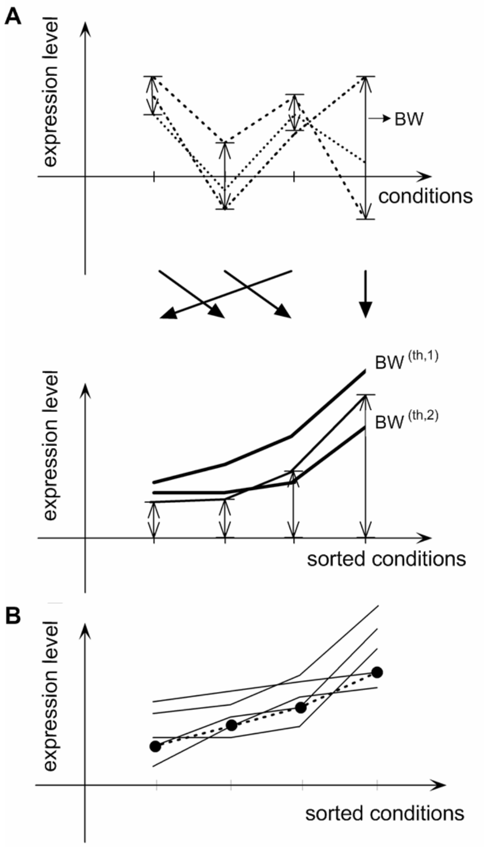

Figure S2: A) Definition of the bandwidth and its use for condition selection
The top half shows hypothetical expression profiles for three different genes. For each of the four conditions, the bandwidth for this set of genes is indicated with a vertical bidirectional arrow. The lower half shows the bandwidth sequence for these expression data, obtained by sorting the bandwidths in increasing order. Two threshold bandwidth sequences ($BW^{(th,1)}$ and $BW^{(th,2)}$) are shown as well (bold lines). If we were to use $BW^{(th,1)}$, this set of three genes would qualify as a module for the expression data, since the bandwidth sequences lies entirely below $BW^{(th,1)}$. However, if we were using $BW^{(th,2)}$, this set of genes would not qualify as a module.
B) Selection of the band width threshold based on random data
To illustrate how a threshold bandwidth sequence is obtained from the data, we show five bandwidth sequences corresponding to randomly sampled sets of genes. The dashed line is a candidate threshold bandwidth sequence, obtained by connecting all second smallest values for all four sorted conditions. The threshold bandwidth sequence that is actually used is the one that qualifies a fixed fraction of randomly sampled gene sets as a module (box p-value).

Determining the threshold bandwidth sequence

To determine the threshold bandwidth sequence $BW_j^{(th)}$, we use the pre-specified probability that a randomly sampled gene set of size G_k passes the module constraint on the expression data ('box p-value', see Running parameters). We randomly select a large number N_S of random gene sets of size G_k, and compute the sorted bandwidth sequence for each set. Subsequently, we determine the *j*'th position of the *b*'th candidate threshold bandwidth sequence as *b*'th largest elements among

all *j*'th positions in those sorted bandwidth sequences. The consequence is that all candidate threshold bandwidth sequences are monotonically increasing, and different candidate threshold bandwidth sequences never intersect. Finally, the threshold bandwidth sequence *b* that achieves our pre-specified probability (or a smaller probability) is selected and used as $BW_j^{(th)}$ in the above procedure. Figure S2b illustrates the threshold bandwidth selection process and the verification of constraint C_C on a hypothetical example.

CHARM based algorithm for closed itemset mining

A naive exhaustive search for valid modules as defined above would require checking all possible combinations of genes, motif instances, and experimental conditions. To drastically restrict the search space without running the risk to skip valid modules, we use the observation that $\mathbf{g}^{(m)}$ can only satisfy the constraints C_G, C_R and C_C if at least all its subsets do so as well. This fact can be restated in terms of the subset relation between gene sets, which represents a partial order relation: 'the module properties vary monotonically over the subset partial order'. A consequence is that we can search for modules by starting with very small gene sets (containing just one gene), gradually expanding them, and stopping (or pruning) the search once a gene set is reached for which one of the module properties is violated. This pruning step results in a massive speed-up, making the method applicable to large data sets.

More formally, we can represent all gene sets and their partial order in a lattice (see Figure S3). This is a directed graph with gene sets as nodes and arrows from any gene sets to its supersets containing one additional gene. The search for modules can then be performed by means of a breadth-first traversal, depth-first traversal, or any other traversal of the lattice. In our previous work we have adopted a breadth-first strategy that resembles the Apriori algorithm [19,65]. In the current work we use a depth-first search more similar to CHARM [44]. The depth-first strategy guarantees a better scalability, especially on non-sparse data sets (such as the expression compendium), which was necessary to obtain the results in the current paper. Even though the exploration of all gene sets has become feasible in this way, the number of modules may still be impractically large. DISTILLER solves this problem by reporting only closed modules. These are modules that cannot be further extended by any other gene without reducing the number of motifs that all of its genes share, or the number of conditions in which its genes are expressed similarly. DISTILLER applies techniques used in CHARM to efficiently ignore non-closed modules while selecting all the closed ones. We report only closed modules with a number of genes larger than or equal to a threshold G (the 'gene content threshold') because only those modules containing a minimum number of genes are interesting.

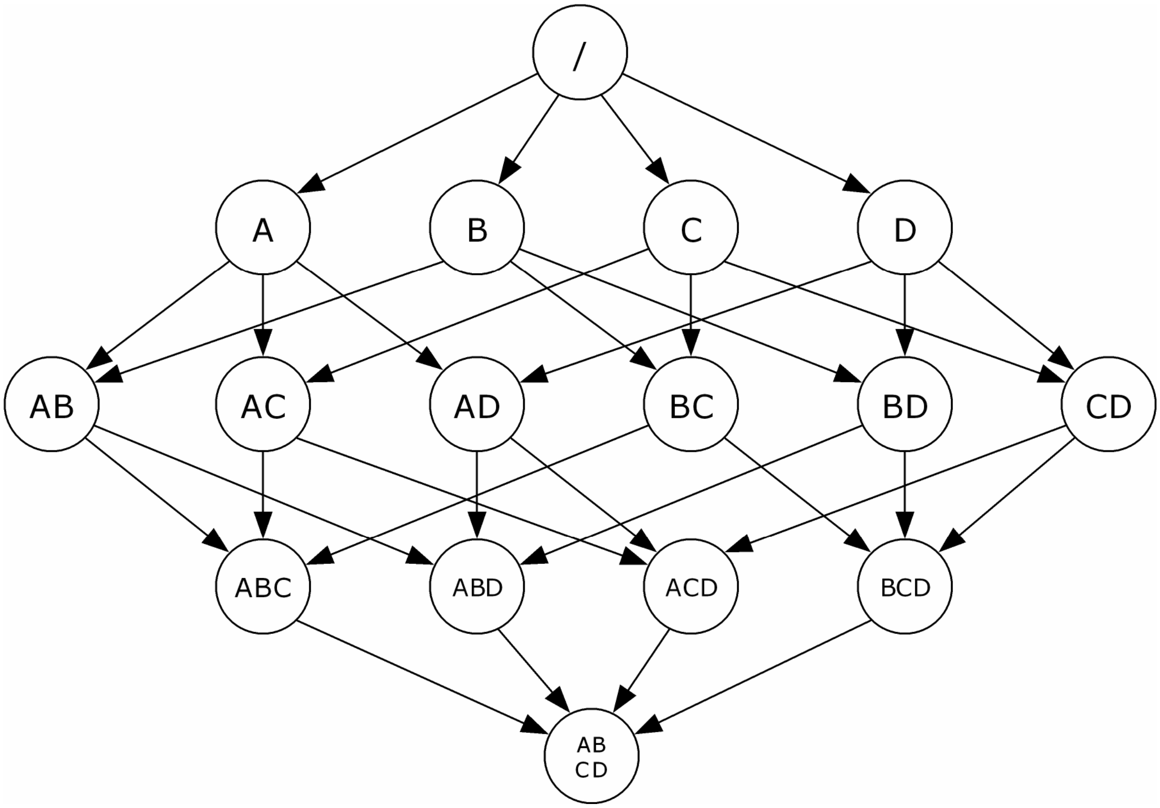

Figure S3: The lattice structure of the space of gene sets.
Four genes are represented by letters A, B, C, and D. Each node corresponds to a gene set (a potential regulatory module). The lattice structure reflects the subset relation: an arrow points from node x to node y if x is a subset of y. The efficiency of our algorithm relies on the fact that if a certain node does not qualify as a module, none of its descendants in the lattice will. This means that we can search for modules by starting at the top of the lattice (with the empty gene set) and exploring it by a depth first traversal. As soon as a gene set is reached that does not qualify as a module, the entire lattice below it can be prune.

2.2 Selecting interesting non-redundant modules

Despite the massive reduction in the number of modules achieved by using CHARM, the output may still be too large to explore: small amounts of noise in the data may cause one module to appear as a large number of separate partially overlapping modules, all very similar in gene, regulator, and condition content. To address this problem we apply an iterative procedure that selects the most interesting modules one by one. It takes into account the significance of individual modules but penalizes at the same time overlap with modules that have already been reported.

We initialize the procedure by attaching an 'interest score' to every module. This interest score is obtained by multiplying the p-value of the module expression pattern with the p-value of the module motif content. For the motif data, the latter p-value is the probability that a module with at least the same number of genes and containing these motifs is found by chance. The corresponding null distribution assumes that each motif is present in all genes with the same probability. We estimate this probability from the data by taking the ratio of the number of genes with the motif at hand to the total number of genes. The p-value of the module motif content can then be computed using the cumulative binomial distribution. For the expression data, the p-value of the pattern is the probability that a module with at least the same number of genes and containing the same conditions is found by chance. The corresponding null distribution is specified as being invariant with respect to permutations of the gene expression values within the same condition. The corresponding p-value of the module expression pattern can then be computed by Monte Carlo sampling. The interest score is obtained by multiplying the p-values for both separate data sources.

The interest score allows ranking all modules according to decreasing significance. However, it is still possible that several mutually redundant (heavily overlapping) modules rank high. In the first step of the iterative procedure the most interesting (top ranking) module is selected. Then, in each of the following steps another module is selected in such a way that it adds as much information as possible to the modules that have already been already selected. To do this, we update the null distribution for the motifs each time a new module has been selected. This is done by changing the probability that a gene has a motif instance to one in the null model, for those entries in the motif matrix that are already covered by previously selected modules. The result is that the interest scores of modules that heavily overlap with previously selected modules increase, such that they move down in the ranking. In every iteration, we select the most interesting module with respect to the corresponding adapted null distribution.

DISTILLER selects conditions in which the genes of a module are significantly co-expressed. However, in some of these conditions the expression ratios of the modules genes will be close to zero, indicating that their expression is not varying (i.e. spurious conditions). Therefore as an additional post-processing step, conditions in a module for which the coefficient of variation (ratio of the gene expression standard deviation over the gene expression mean) exceeded a specified threshold were excluded from the modules.

2.3 Seed module extension

In a subsequent extension step we recruit additional candidate module genes that did not pass the stringent seed discovery step but should be considered part the module (e.g. downstream operon genes that do not contain a motif instance in their promoter regions but are subject to its regulatory influence).

The relaxed criteria for adding additional genes to the module are the following: 1) the gene's expression profile should have a correlation with the module's mean expression profile of at least a fraction α of the module correlation (defined as the lowest correlation value between a seed gene's expression profile and the average expression profile for the modules conditions), and 2) the genes should have a motif instance with p-value below a threshold β. Both requirements have to be fulfilled unless a gene is part of an operon for which the first gene is present in the seed module. In this case only the first criterium has to be satisfied.

2.4 Running parameters

To test the influence of different parameters, we compared results obtained with different settings with known interactions from the RegulonDB network by calculating the recall and precision:

- $Recall = \dfrac{TP}{TP + FN}$
- $Precision = \dfrac{TP}{TP + FP}$

where *TP* = true positives, *FN* = false negatives, *FP* = false positives[*]. In this case, *TP*+*FN* equals the total number of interactions in RegulonDB, i.e. 736 interactions, while *TP*+*FP* is the total number of interactions reported by DISTILLER. The *TP* are confirmed interactions present in RegulonDB that were also identified by DISTILLER. Hence, recall and precision indicate the trade-off between the number of predicted and the percentage of reported interactions that corresponds to known links in the regulatory network. Our goal is to obtain high confident predictions, corresponding to parameter settings that yield a large product of recall and precision. For each parameter setting the 200 best-scoring non-redundant modules were selected (see

[*] Strictly speaking, these interactions could be false positive interactions or novel, yet unknown interactions. By using precision-recall rather than sensitivity-specificity we were able to deal with this uncertainty in a meaningful way.

section 2.2). Subsequently the recall and precision were calculated for the best-scoring module, the two best-scoring modules, the three best-scoring modules, etc. All tested parameter settings are shown in Table S1. We varied the minimum number of genes in a module (i.e. the gene content threshold), the minimum number of conditions in which the genes should be co-expressed, the p-value of the bandwidth threshold and the motif score threshold. The recall for the different parameter settings is shown in Figure S4, the precision in Figure S5 and the product of the precision and recall in Figure S6.

In general, modules obtained with a motif threshold of 0.01 clearly yield a lower recall and precision than modules obtained with motif threshold 0.001. This means that with a lower motif threshold we recover less known RegulonDB interactions (at least, in the top 200 modules) and obtain many more predicted interactions. Since we want to both study the condition dependency of interactions and obtain reliable novel interactions, a motif threshold of 0.001 clearly is the better choice.

Modules obtained with a bandwidth p-value of 0.0001 correspond to a larger recall and a slightly smaller precision than those obtained with a bandwidth p-value that equals 0.00001. From Figure S6 it is clear that the product of recall and precision is clearly better for modules obtained with a lower p-value. These p-values are not as stringent as they may seem at first sight: in a set of 4000 genes, for instance, there are on the order of 1e10 possible sets of three.

Changing the gene content threshold from three to four genes has a positive influence on the recall, while the precision decreases. The product of precision and recall is however better for a gene content threshold of four genes. To verify that condition selection indeed has a beneficial effect, we also tested parameter settings where the minimum number of conditions in a module was set equal to all conditions (870). From Figure S6 it is clear that although a rather high precision can be obtained with all conditions, the recall was low in those runs.

From the above analysis, it was clear that parameter setting 14 was most likely to yield biologically relevant predictions. To choose the number of modules for further biological analysis, we checked how the product of precision and recall changed over the number of modules (see Figure S6). No large changes were observed in the last 50 modules, indicating increasing redundancy of the additionally selected modules. We therefore choose to keep 150 modules in our further analysis.

In summary, parameter settings were selected such that the seed module consists of at least four genes (i.e. gene content threshold) that share at least one motif and 50 conditions. The threshold for the p-value of the motif instances was set to 0.001. In order to choose the box threshold, 100000 randomizations (random sets of four genes) were carried out and the box p-value

threshold was set to 0.0001. In the post-processing steps, a subset of 150 minimally redundant modules was selected, and the threshold for the coefficient of variation was set to 0.6. For the seed module extension step, correlation parameter α was set to 90% and the relaxed p-value threshold β to 0.05.

Table S1: Different parameter settings that were used to assess the parameter sensitivity. **No. conditions**: the minimum number of conditions in which a set of genes should be co-expressed; **Bandwidth p-value**: p-value to determine the threshold on the band width sequence; **Motif threshold**: the minimum p-value a motif instance should have; **No. genes**: the minimum number of genes in a module.

Test	No. conditions	Bandwidth p-value	Motif threshold	No. genes
Parameter setting 1	50	0.00001	0.01	3
Parameter setting 2	50	0.00001	0.01	4
Parameter setting 3	50	0.0001	0.01	3
Parameter setting 4	870	0.00001	0.01	3
Parameter setting 5	870	0.00001	0.01	4
Parameter setting 6	870	0.0001	0.01	3
Parameter setting 7	870	0.00001	0.001	3
Parameter setting 8	870	0.00001	0.001	4
Parameter setting 9	870	0.0001	0.001	3
Parameter setting 10	870	0.0001	0.001	4
Parameter setting 11	50	0.00001	0.001	3
Parameter setting 12	50	0.00001	0.001	4
Parameter setting 13	50	0.0001	0.001	3
Parameter setting 14	50	0.0001	0.001	4

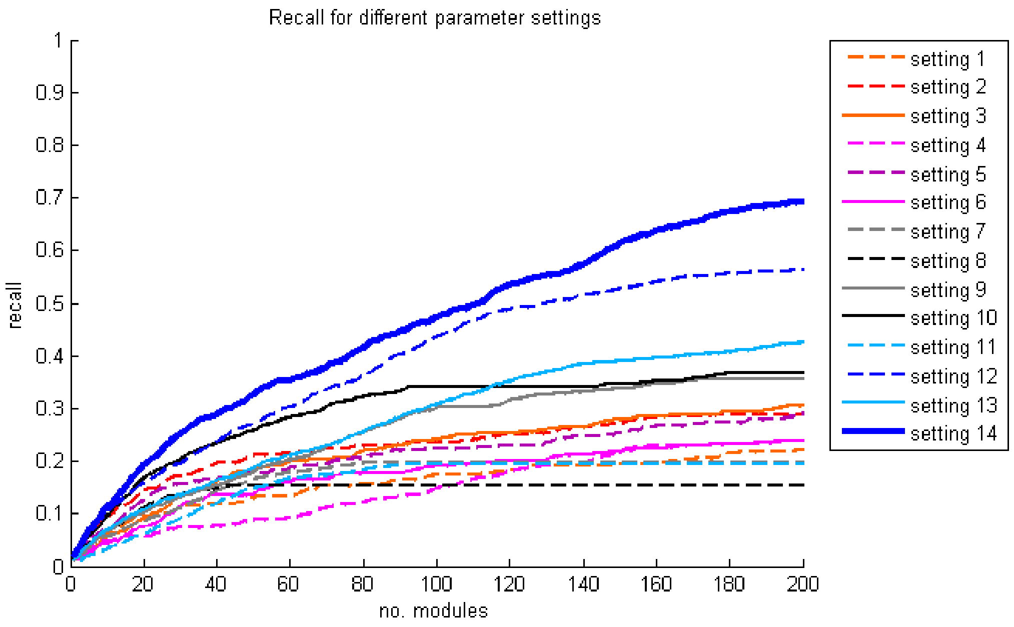

Figure S4: Recall for different parameter settings. For each parameter setting, recall was calculated for a selection of up to 200 best-scoring non-redundant modules. Parameter setting 14 was selected for further use in the paper. Clearly, recall was best for this parameter setting.

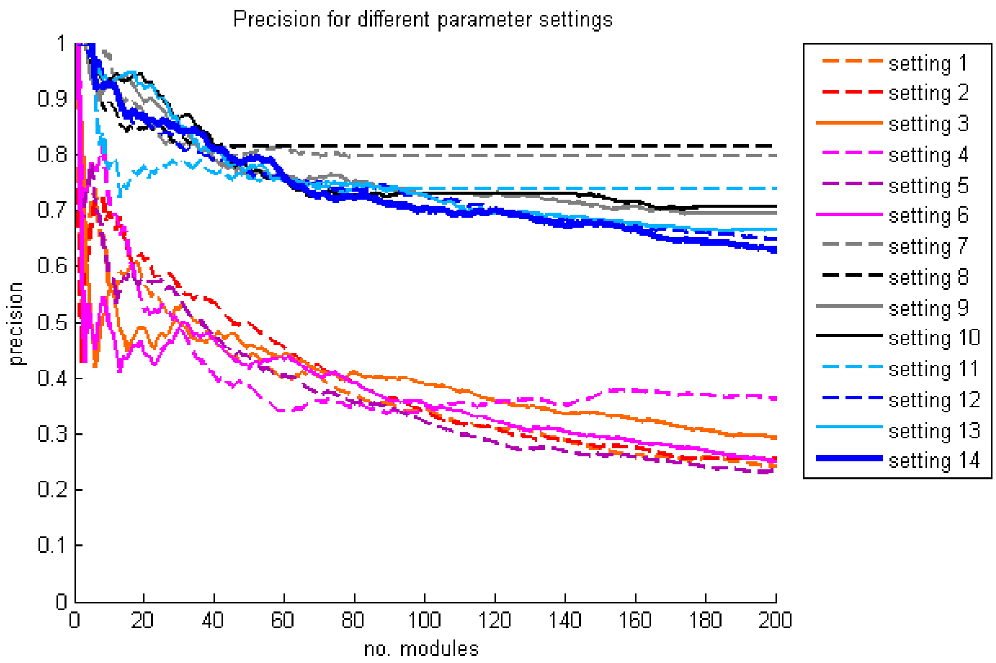

Figure S5: Precision for different parameter settings. For each parameter setting, precision was calculated for a selection of up to 200 best-scoring non-redundant modules. Precision of parameter setting 14 is better than those of parameter setting 1-6, but it is not optimal. Figure S6, however, illustrates how the product of precision and recall still favors setting 14 over the other parameter settings.

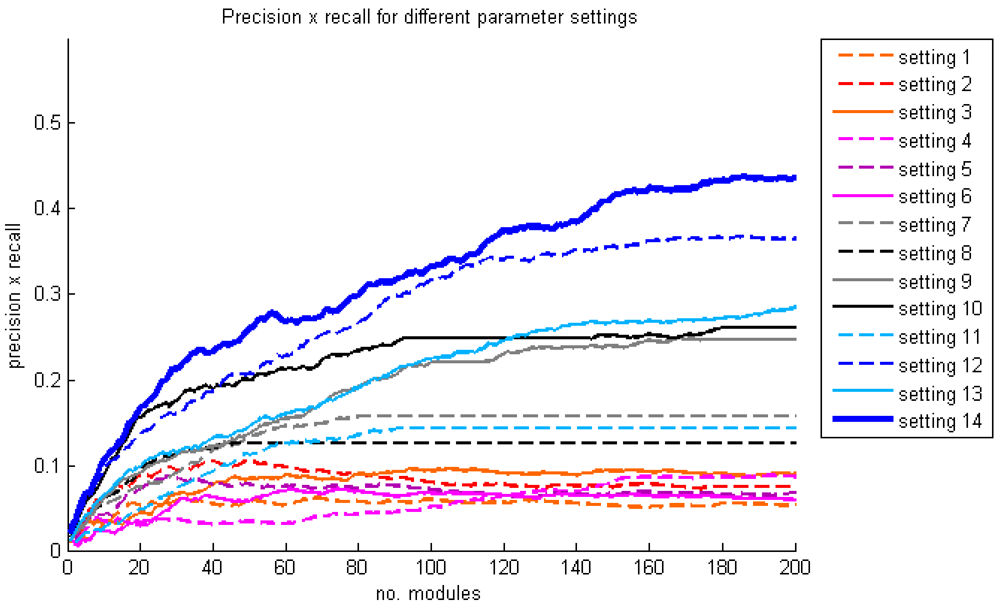

Figure S6: Product of precision and recall for different parameter settings. For each parameter setting, precision and recall were calculated for a selection of up to 200 best-scoring non-redundant modules. This plot allows to check for the trade-off between good precision and good recall. Parameter setting 14 outperforms the other settings and was therefore selected for futher use in the remainder of the paper.
