# Supplementary material for: DISTILLER: a data integration framework to reveal condition dependency of complex regulons in Escherichia coli
Source: Genome Biol. 2009 Mar 6;10(3):R27. doi: 10.1186/gb-2009-10-3-r27 (PMC2690998; doi:10.1186/gb-2009-10-3-r27)
Supplement: Additional data file 8 — How the predicted FNR targets were experimentally validated. [file gb-2009-10-3-r27-S8.pdf]

ADDITIONAL DATA FILE 8: Experimental validation of predicted FNR targets

To demonstrate the reliability of our approach, we used ChIP-qPCR to validate predicted interactions for FNR. We tested 11 predicted FNR targets that were selected based on their difference in prediction confidence. These 11 predictions, consisting of five high confidence predictions (*ydhY*, *yfgG*, *hscC* and *treF*) originating from three modules containing more than 50% known FNR targets genes and eight low confidence predictions (*yjhB*, *ydjX*, *yjtD*, *ydaT*, *yehD*, *yhjA* and *ftnB*) resulting from two modules with only one known target were all shown to bind FNR (see Table S1).

E. coli MG1655 [66] and JCB1101, a MG1655 encoding *fnr*3XFLAG [67] cells were grown under aerobic (250 ml conical flasks containing 50 ml Luria-Bertani (LB) medium supplemented with 0.4% glucose, shaken at 200 r.p.m.) or microaerobic (250 ml conical flasks containing 150 ml LB medium shaken at 100 r.p.m. conditions at 37°C until an OD_{595} of 0.4 was reached. Chromatin immunoprecipitation was performed as described in Thijs *et al.* [46], except that the tagged FNR was immunoprecipitated with anti-FLAG M2 antibodies (Sigma, F1804). Immunoprecipitation was performed for six biological replicates of both MG1655 wild-type (referred to as 'mock') and the *fnr*3XFLAG strain (referred to as 'IP').

Physical interaction between FNR and the selected targets genes was determined by quantitative real time PCR (qRT-PCR). qRT-PCR was performed on a StepOnePlus instrument (Applied Biosystems) in a 20 µl reaction mixture containing 1 µl of the IP- or mock-DNA preparation, 900 nM of each primer and 10 µl Power SYBR Green kit (Applied Biosystems). A standard qRT-PCR run consisted of a holding stage to activate enzyme for 10 minutes at 95°C, followed by 40 cycles of 15 seconds at 95°C and 1 minute at 60°C.

Primers for qRT-PCR, designed with PrimerExpress (Applied Biosystems) are displayed in Table S2. The well known FNR targets *ndh* and *pdhR* [24,68,69] were chosen as positive controls. Negative controls consisted of genes for which no evidence of FNR-mediated transcription regulation exists. Primers were targeted against the coding regions of these genes in order to avoid overlap with potential FNR binding sites in the intergenic

regions. For the predicted targets, the intergenic sequence surrounding the predicted FNR motif was amplified.

To evaluate the enrichment of DNA sequences bound to FNR, the initial concentrations (N_0) were compared between IP and mock for both aerobic and microaerobic growth conditions. For each amplification reaction N_0 was estimated by performing a linear regression on the logarithm of the fluorescence intensities per cycle number [70]. This linear regression was fitted on 4 subsequent measurements, which were selected as the ones showing the highest slope (i.e. 4 measurements in the 'window-of-linearity'). To account for sample variation, N_0 values were rescaled by a sample-specific factor that renders the mean N_0 of 11 negative controls equal for all biological replicates of both aerobic and microaerobic growth conditions. To asses the validity of FNR binding to a specific target, *p*-values were calculated by performing a *t*-test on the mean logratios of IP over mock for both growth conditions. An interaction was deemed significant if its *p*-value was below 0.05.

Table S1: The p-values and logratios (IP versus mock) that were obtained for each gene in both aerobic and micro-aerobic conditions are shown.

gene	description	p-value		logratio	
		Aerobe	LB-Micro-aerobe	Aerobe	LB-Micro-aerobe
ydhY (b1674)	Module 80	0,0212	0,0079	0,8210	0,7399
yfgG (b2504)	Module 80	0,0137	0,0102	0,8896	0,6498
hscC (b0650)	Module 83	0,0611	0,0185	0,5999	0,6292
ydaT (b1358)	Module 84	0,0359	0,0254	0,7421	0,6873
ydjX (b1750)	Module 84	0,0127	0,2657	0,7355	0,2040
yjhB (b4279)	Module 84	0,0012	0,0115	0,9176	0,5977
yjtD (b4403)	Module 84	0,1095	0,0168	0,2186	0,4854
treF (b3519)	Module 86	0,0100	0,0484	0,4816	0,4141
ftnB (b1902)	Module 87	0,0261	0,1399	0,3379	0,1721
yehD (b2111)	Module 87	0,0044	0,4933	0,7689	0,0055
yhjA (b3518)	Module 87	0,0092	0,0408	0,7195	0,5711
endA (b2945)	Neg. control	0,3171	0,1548	0,0680	0,0993
kefB (b3350)	Neg. control	0,7461	0,6144	-0,0417	-0,0092
metC (b3008)	Neg. control	0,0695	0,4546	0,1949	0,0171
php (b3379)	Neg. control	0,9328	0,3735	-0,2913	0,0162
rfaH (b3842)	Neg. control	0,7671	0,6362	-0,2136	-0,0670
secD (b0408)	Neg. control	0,7727	0,6867	-0,1616	-0,0884
tolA (b0739)	Neg. control	0,1174	0,0765	0,1070	0,0739
yafZ (b0252)	Neg. control	0,8678	0,7786	-0,0445	-0,0214
ybhC (b0772)	Neg. control	0,2230	0,6324	0,0581	-0,0080
yeiM (b2164)	Neg. control	0,7793	0,5028	-0,0437	-0,0002
yfcI (b2305)	Neg. control	0,2266	0,4794	0,0771	0,0038
ndh (b1109)	Pos. control	0,0824	0,0001	0,5162	0,6667
pdhR (b0113)	Pos. control	0,0062	0,0616	0,3400	0,3841

Table S2: The set of genes that were tested in the ChIP experiment and their forward and reverse primers are shown. Among the genes are predicted FNR targets, negative controls and positive controls.

Genes	Description	No of known FNR targets in module	forward primer	reverse primer
ydhY (b1674)	Module 80	2	GCAATGAAAGTCACAAATAATTGTCG	TCTGAGCCCTTAACATTGATCGTT
yfgG (b2504)	Module 80	2	ATTCAGACAGGCATCCCACCT	AAAGCGTCAGTGGATACATATTTTAATG
hscC (b0650)	Module 83	3	CATTGATTAAGATCACCACGCG	CCATTGCATTTATATCTTGAAAGAAAA
yjhB (b4279)	Module 84	1	TGGAGGAAAAGATTGTACTGATTAGGT	GGAGCCTCAACAATATCCAGAAA
ydjX (b1750)	Module 84	1	GCTCAAACTGGGTCAGGAGAAT	GGCAGGCAACAAATCATTACTG
yjtD (b4403)	Module 84	1	GGCCGGACACCCAATAAAA	TTTCATCAAACTGTTAACGTGCTACA
ydaT (b1358)	Module 84	1	TCCTCATTTGATCATACCTGAAACA	GATGTGCTCATGCTTGATTTTCAT
treF (b3519)	Module 86	3	CAGGGAGATACGCTTTTTGATAGG	TGCCATTCCTGATCTGGGTAA
yehD (b2111)	Module 87	1	GCATTTAGAAAGCCGAAATCATTTATA	TCTCACATCAAAAAAGTTGCCG
yhjA (b3518)	Module 87	1	TGCCATTCCTGATCTGGGTAA	CAGGGAGATACGCTTTTTGATAGG
ftnB (b1902)	Module 87	1	ACGTATAATGAGAGCCATCTCGC	CGGGTATAAGAAAATGCTTAAATCATC
metC (b3008)	Neg. control		GGCTATTTTCCTTTGTGCTTA	CCCACGAGTAGGCCATGCTGAA
rfaH (b3842)	Neg. control		TCAGCCACTTCGTGCGCTT	TGCCGGATCGACAATGTCTT
yafZ (b0252)	Neg. control		CCCTGCTCGACAGCCTACA	CGGGTCTGACAGGCAAAGAA
kefB (b3350)	Neg. control		TCAGGTGATTGGTCGTTTGC	CCGCTCCAGCACGGTAAT
yfcI (b2305)	Neg. control		CGGCAATGCAAAACCATCTT	TCGGGAGCACCAATGGAA
secD (b0408)	Neg. control		GCGCGTGCGAAAGAGATT	GGTGTTTACCAGACGGAATTCC
endA (b2945)	Neg. control		AAGGCCAGTACGGTCAATGC	TGGTTCGGCAGCTTTTTCTT
php (b3379)	Neg. control		TGGTGGCCTGTACCGGTTAT	GCGGGTCGCCACATGT
yeiM (b2164)	Neg. control		TTGGCGGCTGGTTTGGT	CAGCACATAGCCAAAAATACTTTCC
ybhC (b0772)	Neg. control		ACGATAGCTGCCAGAGCAAAC	AGAAGACCGCAGAGCAGAGAA
tolA (b0739)	Neg. control		TCGACGCTGTCATGGTTGAT	GCTTTGCATGCGTTTGTACTG
ndh (b1109)	Pos. control		CCCGGTAAAGTCGCCTATCTT	GAATGGTGCATTGGTCATACCA
pdhR (b0113)	Pos. control		ATGTGCACAGTTTCATGATTTCAA	TGTGACGCTAAAGTAACAAAGTATTCAC
